# Electroconvulsive therapy for the acute management of severe agitation in dementia (ECT-AD): A modified study protocol

**DOI:** 10.1371/journal.pone.0303894

**Published:** 2024-06-28

**Authors:** Maria I. Lapid, Julia Merrill, Martina Mueller, Adriana P. Hermida, Louis Nykamp, Jason Andrus, Heela Azizi, Paula Bolton, Nana Bonsu, Raphael Braga, Catherine R. Dillon, Donna Ecklesdafer, Darci Evans, David Harper, Hannah Heintz, Sehba Hussain-Krauter, Olivia Holzgen, Daniel Humphrey, Salima Jiwani, Emily K. Johnson, Simran Kang, Janelle Kassien, Jonathan Kim, Rebecca G. Knapp, Simon Kung, Neil Kremen, Kendra Le, Jack Mahdasian, Taylor Marzouk, Jared D. Masrud, Jefferson Mattingly, Dawn Miller, Sandeep R. Pagali, Regan Patrick, Patricio Riva Posse, Cristina Pritchett, Aniqa Rahman, Swapnil Rath, Cara Roczniak, Teresa A. Rummans, Sohag Sanghani, Steve Seiner, LeAnn Smart, Evan Tomaschek, Valeriya Tsygankova, Lori VanderSchuur-White, Monica P. Walton, James Wilkins, April Williams, Sarah M. Williams, George Petrides, Brent P. Forester

**Affiliations:** 1 Department of Psychiatry and Psychology, Mayo Clinic, Rochester, Minnesota, United States of America; 2 McLean Hospital, Belmont, Massachusetts, United States of America; 3 College of Nursing, Medical University of South Carolina, Charleston, South Carolina, United States of America; 4 Department of Public Health Sciences, Medical University of South Carolina, Charleston, South Carolina, United States of America; 5 Department of Psychiatry and Behavioral Sciences, Emory University School of Medicine, Atlanta, Georgia, United States of America; 6 Pine Rest Christian Mental Health Services, Grand Rapids, Michigan, United States of America; 7 Northwell, New Hyde Park, New York, United States of America; 8 Department of Psychiatry at the Zucker Hillside Hospital, Glen Oaks, New York, United States of America; 9 Department of Psychiatry, Donald and Barbara Zucker School of Medicine at Hofstra/Northwell, Hempstead, New York, United States of America; 10 Institute of Behavioral Science, Feinstein Institutes of Medical Research, Manhasset, New York, United States of America; 11 Harvard Medical School, Cambridge, Massachusetts, United States of America; 12 Ican School of Medicine at Mount Sinai, New York, New York, United States of America; 13 Division of Hospital Internal Medicine, Department of Medicine, Mayo Clinic, Rochester, Minnesota, United States of America; 14 Oregon Health & Science University, Portland, Oregon, United States of America; 15 RWJBarnabas Health System, Trinitas Regional Medical Center, Elizabeth, New Jersey, United States of America; 16 Tufts Medical Center, Tufts University School of Medicine, Boston, Massachusetts, United States of America; PLOS: Public Library of Science, UNITED KINGDOM

## Abstract

**Objective:**

This study began as a single-blind randomized controlled trial (RCT) to investigate the efficacy and safety of electroconvulsive therapy (ECT) for severe treatment-refractory agitation in advanced dementia. The aims are to assess agitation reduction using the Cohen-Mansfield Agitation Inventory (CMAI), evaluate tolerability and safety outcomes, and explore the long-term stability of agitation reduction and global functioning. Due to challenges encountered during implementation, including recruitment obstacles and operational difficulties, the study design was modified to an open-label format and other protocol amendments were implemented.

**Methods:**

Initially, the RCT randomized participants 1:1 to either ECT plus usual care or simulated ECT plus usual care (S-ECT) groups. As patients were enrolled, data were collected from both ECT and simulated ECT (S-ECT) patients. The study now continues in an open-label study design where all patients receive actual ECT, reducing the targeted sample size from 200 to 50 participants.

**Results:**

Study is ongoing and open to enrollment.

**Conclusion:**

The transition of the ECT-AD study design from an RCT to open-label design exemplifies adaptive research methodologies in response to real-world challenges. Data from both the RCT and open-label phases of the study will provide a unique perspective on the role of ECT in managing severe treatment-refractory agitation in dementia, potentially influencing future clinical practices and research approaches.

## Introduction

Alzheimer’s disease (AD), the most prevalent neurodegenerative disease of aging, affects approximately 6.2 million individuals in the US; the prevalence is projected to increase to 12.7 million individuals by 2050 [1]. Vascular dementia accounts for about 20% of all dementia cases [2], while dementia with Lewy Bodies and frontotemporal dementia make up approximately 4% [3] and 3% [4] of dementia cases, respectively. Neuropsychiatric symptoms (NPS) of dementia, which include agitation, depression and apathy, significantly worsen morbidity and mortality, magnify public health burden [5, 6] and adversely impact caregivers.

Agitation, a prevalent NPS in various dementia subtypes [7–9] drives substantial health and social care costs, approximately $31 billion annually [1, 10]. This proposal targets agitation in dementia due to its high prevalence, importance to caregivers, and implications for safety and quality of life. Agitation includes both aggressive or non-aggressive behaviors [11, 12]. Aggressive behaviors include fighting, throwing, grabbing, destroying items, verbal outbursts, cursing, and screaming [12]. Non-aggressive agitated behaviors include restlessness, pacing, wandering, repetitive questioning, chatting, inappropriate disrobing, and verbal outbursts [12]. Nearly all individuals with AD develop NPS over the course of the disease [13] with agitation contributing to deterioration of family and professional relationships [14], increased risk of death [15], and heightened caregiver burden, often leading to institutionalization or death [16].

Effective treatments for severe agitation in advanced dementia are urgently needed. Behavioral therapies, the first-line recommendation, often take time and may be less effective in severely agitated patients [17]. Psychotropic medications, particularly antipsychotics, are commonly used but have inconsistent efficacy [18, 19] and serious risks, including increased mortality [20, 21]. Brexpiprazole is an atypical antipsychotic that was recently FDA approved for the treatment of agitation associated with dementia due to AD [22]. Antidepressants like selective serotonin reuptake inhibitors (SSRIs) are safer but may be complicated by cardiac conduction-delaying effects [23]. The multi-center randomized clinical trial of citalopram for agitation in AD (CitAD) demonstrated citalopram reduced agitation, but effects were only evident after 6–9 weeks and were limited to outpatients with less severe symptoms [23].

Given the pressing need for new treatments for severe, treatment-resistant agitation in dementia, we initially designed a single-blind, randomized trial to evaluate electroconvulsive therapy (ECT) for inpatients with severe agitation and moderate to severe dementia, who have not responded to psychotropic medications [24]. This manuscript details the original protocol (ECT-AD) and outlines the significant modifications that were later necessitated. These changes, primarily driven by recruitment challenges and practical consideration in implementing the study, underscore the adaptive nature of clinical research. If ECT is found to be safe and effective for severe agitation in dementia, both the original and modified study designs could provide important public health insights, with potential significant benefits for patients, families, and society at large.

### Justification for ECT in the management of severe agitation in dementia

ECT is the brief application of an electrical pulse to the scalp in order to excite brain cells and cause them to fire in unison, producing a short-lasting seizure under controlled conditions and general anesthesia [25]. ECT has been shown to be effective in psychiatric disorders like depression, schizophrenia, and catatonia. The effects of ECT on multiple neurotransmitter systems [26] (GABA, glutamate, serotonin, norepinephrine, dopamine) and down-regulation of immune activation [27, 28] suggest a role for ECT in the attenuation of agitation in dementia, hypothesized to stem from neurotransmitter dysfunction [11, 23] and neuroinflammation [29]. The safety and efficacy of ECT in geriatric mood and psychotic disorders are well-established, making it a gold standard in psychiatry [25, 30, 31]. It is recommended by the American Psychiatric Association (APA) for urgent cases, treatment-resistant conditions or patient preference [25, 31, 32]. Studies show its effectiveness in older adults, particularly in major depressive disorder (MDD), even beyond age 85. ECT has been found to be more effective in older than younger MDD patients [31, 33–36], with evidence suggesting longer survival and greater clinical improvement [30, 31]. The NIH-funded Prolonging Remission in Depressed Elderly (PRIDE) study also confirmed the safety and efficacy of right unilateral ultra-brief pulse ECT (RUL-UB) for older adults with MDD [37, 38, 39].

### ECT efficacy and safety data for severe agitation in dementia

When behavioral interventions and pharmacotherapies fail, ECT emerges as a safe and effective treatment option for severe agitation in dementia [40–53]. Studies demonstrate significant agitation reduction in dementia patients using ECT, with minimal serious adverse events [41]. A multi-site, prospective case series study showed 18 out of 23 patients with dementia experienced reduced agitation post-ECT as measured by the Cohen Mansfield Agitation Inventory (CMAI), despite three discontinuations due to adverse events [27, 54, 55]. Other studies echo these results; for instance, 9 out of 11 AD patients achieved remission of agitation after ECT with sustained results over a year for most [40, 44, 48]. A retrospective review of 60 individuals with dementia undergoing ECT revealed significant improvements with few complications like transient confusion [56]. ECT was generally well-tolerated, even with variations in ECT techniques and patient demographics [38, 41]. These findings, showing minimal serious side effects and substantial agitation reduction, highlight the potential of ECT as an acute treatment for severe agitation in dementia, warranting further research through randomized-controlled trials in larger cohorts [52]

### Rationale for ECT-AD study

The rationale for exploring ECT in this context is based on its established efficacy in various psychiatric disorders and its potential role in mitigating agitation in dementia. Existing literature provides preliminary evidence that ECT is a viable option for patients with severe, treatment-resistant agitation in dementia. Recognizing these possibilities and the need for more comprehensive data, we formulated specific aims to investigate the efficacy and safety of ECT in this patient population.

## Materials and methods

Our study is designed to address critical gaps in the treatment of severe agitation in dementia, specifically focusing on the application of ECT. The study protocol (S1 and S2 Files) was approved by the Mass General Brigham IRB as the single Institutional Review Board (sIRB) of record on August 28, 2020 under Protocol #2020P002276 (S3–S6 Files). All consent procedures across participating sites adhere to the guidelines of the sIRB. Written consent was obtained by the legally authorized representatives (LAR) for all study participants.

### Specific aims

#### Original aims

1. **Efficacy of ECT in reducing agitation**: The primary aim was to assess the efficacy of up to 9 ECT combined with usual care (ECT+UC) compared to Simulated ECT and usual care (S-ECT+UC) in reducing severe agitation in 200 participants with moderate to severe Alzheimer’s dementia. The hypothesis is that ECT+UC will effectively reduce severe agitation, as measured by the CMAI total score as the primary outcome, and Alzheimer’s Disease Cooperative Study-Clinical Global Impression of Change scale (ADCS-CGIC) [57, 58], Neuropsychiatric Inventory Clinician Scale (NPI-C) [59], and Pittsburgh Agitation Scale (PAS) as secondary outcomes [60].
2. **Tolerability and safety of ECT**: The second aim was to compare the tolerability and safety of ECT+UC versus S-ECT+UC in the same participant group. The hypothesis was that there will be no difference in tolerability and safety outcomes, including cognitive decline (Severe Impairment Battery-8 [SIB-8]), development of delirium (Confusion Assessment Method [CAM]), and serious adverse events, between the two approaches.
3. **Exploratory aim**: An exploratory aim was to explore the long-term stability of agitation reduction (CMAI) and global functioning (Clinical Global Impression-Severity [CGI-S]) with assessments at 1, 3, 6, and 12 months post-ECT.

#### Revised aims (following protocol modifications)

Due to recruitment challenges and practical difficulties in implementing sham procedures, the study design was revised from a randomized controlled trial (RCT) to an open-label approach. Consequently, the specific aims were adjusted as follows:

1. **Effect of ECT on reducing agitation**: The primary aim was revised to determine the effect of up to 9 ECT+UC on severe agitation in up to 50 subjects with moderate to severe dementia including Alzheimer’s disease, vascular dementia, frontotemporal dementia, and dementia with Lewy bodies, expanding beyond Alzheimer’s disease to other types of dementia. The hypothesis remained unchanged.
2. **Tolerability and safety of ECT**: The second aim was revised to determine the tolerability and safety of ECT+UC in up to 50 subjects with moderate to severe dementia including Alzheimer’s disease, vascular dementia, frontotemporal dementia, and dementia with Lewy bodies. The hypothesis is that ECT will be well tolerated and safe as measured by cognitive decline (SIB-8), development of delirium (CAM), and serious adverse events.
3. **Exploratory aim**: There were no changes to the exploratory aim.

### Design

Building on our specific aims, both original and revised, we developed a study design to effectively address these objectives under the evolving conditions of our research environment. The evolution of the study design from an RCT to an open-label study was an adaptive research strategy that was necessary to continue to achieve our objectives and maintain robust methodologies. In the following section, we detail the study design, outlining both original plan and the subsequent modifications, to provide a comprehensive understanding of our methodological approach and response to challenges encountered.

### Original study design

#### Trial design

Originally conceptualized and subsequently implemented as a single-blind RCT, the study aimed to compare the efficacy and safety of ECT against S-ECT in patients with moderate to severe Alzheimer’s dementia (AD) exhibiting severe agitation.

#### Study setting

The original setting for our study was exclusively psychiatric inpatient units where patients are admitted due to their severe agitation in the setting of advanced dementia. These individuals require a level of care and monitoring that only specialized psychiatric care settings could provide.

#### Sample size

In the original design of our study, we anticipated receiving approximately 210 referrals. Our aim was to randomize 200 participants, equally divided into two groups for the study. This target was set accounting for a projected 20% dropout rate, predicting that about 160 participants (80 per group) would complete the full course of up to 9 ECT treatments. This sample size was calculated to detect a standardized effect size of 0.37 standard deviations (SD) with 85% power. This calculation assumes complete data across three post-randomization measurement time points, a level of significance (alpha) set at 0.05 for a two-tailed comparison, and an interclass correlation within participants no larger than 0.50, with an autoregressive (AR(1)) covariance structure. Specifically, for the primary outcome (CMAI), this sample size enables the detection of a raw effect size difference of 3.4 points [41]. Similarly, for the SIB-8 outcome, we can detect a difference of 1.26 points between the ECT and S-ECT groups [61]. To further account for potential attrition and the dilution effect in intent-to-treat (ITT) analyses, the sample size was increased by 20%, leading to 100 subjects in each intervention group (total N = 200). The PASS software (PASS; NCSS statistical software, 2008, Kaysville, UT, USA) was used for the sample size calculation.

#### Participants

Eligibility criteria included hospitalized patients between 55–89 years old who have a clinical diagnosis of dementia due to AD in moderate to severe stages with Folstein Mini-Mental Status Exam (MMSE) scores of 15 or less, severe agitation levels indicated by CMAI scores of 5 or more on at least one aggressive or physically nonaggressive but potentially dangerous behaviors, and treatment-refractoriness defined as failure of at least three different pharmacological treatments from various drug classes (including antidepressants, antipsychotics, anticonvulsants, prazosin, and cannabinoids) at therapeutic doses for at least two weeks. Medical stability for safe administration of ECT is a prerequisite, confirmed by standard medical assessments. Participants must comprehend English and have a legally authorized representative (LAR) who can provide informed consent. Exclusion criteria were based on clinical consideration such as delirium, history of stroke-related vascular dementia, or lifetime or current diagnosis of schizophrenia, bipolar disorder or schizoaffective disorder. Active substance use disorder or prior ECT or other neurostimulation therapies within the past 3 months also disqualify potential participants.

#### Sample selection

Subject enrollment for the study was initially targeted at individuals with dementia due to Alzheimer’s disease who were admitted to inpatient psychiatry units at designated study sites and meet eligibility criteria. The diagnosis of moderate to severe dementia is confirmed by a board-certified psychiatrist or neurologist by using the NIA-AA criteria [62], informant reports, and an MMSE score of 15 or lower. The assessment ensures that symptoms are not due to delirium or a primary psychiatric disorder. Participants undergo a standard medical screening, including physical examination, urinalysis, and serum chemistries. CMAI scores determine eligibility based on agitation levels. Subjects are expected to lack capacity to provide informed consent for ECT and study participation, hence consent will be obtained from the LAR in accordance with local state laws and hospital guidelines.

#### Randomization

In the original RCT, the plan was to randomize 200 participants 1:1 to ECT+UC or S-ECT+UC using a web-based covariate adaptive randomization algorithm that can control for serious imbalances in covariates considered to have an impact on outcome (e.g. site, antidepressants and antipsychotics use) [63]. At the time of randomization, imbalances in these covariates were to be accounted for using a minimal sufficient balance approach [64]. The randomization was carried out centrally at the Data Center (DC) in the Data Coordination Unit (DCU) at the Medical University of South Carolina (MUSC) using an internally developed, ECT-AD study specific, web-based clinical trials management system (CTMS) referred to as WebDCU^™^. Clinical site study coordinators (SC) log into the WebDCU^™^ study database, enter the eligibility and randomization forms and receive the computer-generated randomization assignment. At the clinical centers, the unblinded SC and site Principal Investigator (PI) have passwords that give access to this assignment.

#### Blinding

To ensure adequate blinding of the inpatient treatment team and clinical raters, the S-ECT process was developed to closely mirror the actual ECT procedure [65]. Participants in the S-ECT group were transported to the ECT suite following the same schedule as those receiving ECT, and they spent equivalent time in the suite to prevent unblinding based on treatment duration. Both groups underwent similar pre-treatment preparations, including medical clearance and fasting protocols. During the S-ECT process, an IV was placed, paralleling the ECT procedures and allowed for the administration of PRN medications and fluids as needed. An unblinded study coordinator accompanied each participant and provided ECT staff with essential information such as recent behavior and rating scale scores. A supervising, unblinded clinician provided clinical management of the S-ECT participants in the treatment suite. The same clinical and standard of care approach to agitation and behavioral management were applied to both S-ECT and ECT groups. Detailed records of the S-ECT procedures were kept separately and securely to ensure the integrity of the blinding mechanism.

### Processes, interventions, and comparisons

#### Pre-randomization assessments

Prior to randomization into ECT or S-ECT, participants undergo assessments for suitability for ECT, anesthesia safety, and overall medical condition [66]. Consents for ECT and research are obtained from the LAR.

#### ECT procedure

The ECT procedure involves the use of either the MECTA spECTrum 5000Q (MECTA Corporation, Oregon) or Thymatron System IV (Somatics, LLC, Illinois) devices and standard ECT protocols [25, 67]. The ECT protocol consists of up to 9 sessions, administered 3 times per week or less frequently, with the acute phase ending upon reaching remission criteria defined by a score of 2 or less on all CMAI items or based on physician judgment. Stimulation parameters include RUL-UB (pulse width 0.25–0.37ms). At the first ECT session, seizure threshold (ST) is determined using the empirical dose titration method [68] and subsequent treatments are approximately 6 times the ST, adjusted according to seizure quality and treatment response.

#### Rationale for ECT treatment protocol

This protocol is informed by two previous studies of ECT in dementia, which demonstrated significant remission of behavioral symptoms by the 9th ECT [40, 41, 56]. RUL-UB ECT was chosen for efficacy similar to bilateral (BL) ECT [40, 41, 69, 70] and minimizing cognitive effects [39, 54, 71]. If the response is inadequate after 6 sessions based on ADCS-CGIC score of 4–6 (indicating no change or worse), electrode placement will switch to bitemporal [40, 56]. An ADCS-CGIC score of 7 at any time will trigger discontinuation of ECT or S-ECT sessions.

#### Monitoring and safety measures

Both motor and electroencephalographic (EEG) monitoring will be performed to ensure adequate seizure duration as defined by a motor seizure greater than 15 seconds. Standard anesthesia and muscle relaxation techniques are employed, and all medications are documented. ECT can be halted for safety or ethical reasons. Treatment emergent effects (e.g., nausea, headache, blood pressure changes) are managed according to standard protocols, and PRN medications are available for acute agitation management. Post-ictal agitation, a short-lived reaction post-seizure, is managed through supportive management or pharmacological intervention with PRN medications, applicable to both ECT and S-ECT groups.

#### Comparative approach

In the original design, these processes and protocols were mirrored as closely as possible in the S-ECT group to maintain the integrity of the comparison and blinding within the study.

#### Schedule of procedures

The study commenced with a comprehensive screening phase, spanning a 7-day window to determine subject eligibility. During this initial phase, LARs provide informed consent on behalf of the subjects, who are then assigned unique identification numbers. Assessments to confirm eligibility include diagnosis, medical history, physical examination, urinalysis, serum chemistries, and electrocardiogram. Cognitive and behavioral screening tests include MMSE, CMAI, and Barthel Index of Activities of Daily Living (BI). Reasons for any screen failures are documented. Following screening, eligible subjects were then randomized on Day 0 into either ECT or S-ECT, with certain assessments possibly repeated if the baseline visit was within 72 hours of the screening visit. The ECT treatments are scheduled three times per week, unless clinically indicated otherwise. Post-ECT assessments using the CMAI, ADCS-CGIC, PAS, NPI-C, Cornell Scale for Depression in Dementia (CSDD), Quality of Life in Late-Stage Dementia (QUALID), SIB-8, CAM, BI and the Family Confusion Assessment Method (FAM-CAM) occur at least 24 hours after the 3rd, 6th and 9th ECT. In instances where participants either terminate early or complete the acute phase prior to completing 9 ECT, a thorough early termination visit is conducted using assessments similar to those performed post-ECT. Throughout the study, rigorous safety monitoring is performed daily through chart reviews and delirium assessment. Parameters for safety include daily assessment of delirium using the CAM, adverse events, serious adverse events, and assessment of cognition with the SIB-8 or BI. Finally, the study design incorporates a naturalistic follow up phase, with assessments occurring at 1, 3, 6, and 12 months post-ECT. During these follow-up visits, data are collected using Zarit Caregiver Burden Scale, BI, QUALID, CGI-S, and CMAI. Additionally, any occurrences of serious adverse events are closely monitored and recorded.

#### Outcome measures

In the study, outcome measures are used to evaluate the efficacy and safety of the treatments, regardless of the participant’s ability to complete them. This approach accommodates subjects with dementia who may struggle with the questionnaires. Key measures include:

- CMAI [11, 72]: assesses agitation and aggression in dementia, covering physically and verbally aggressive and non-aggressive behaviors [73, 74].
- ADCS-CGIC [57, 58]: measures clinically relevant change over time in dementia patients.
- Zarit Caregiver Burden Interview [75]: a self-report measure for caregivers to report degrees of burden of providing care.
- QUALID [76]: assesses quality of life in late-stage dementia.
- PAS [60]: measures agitation symptoms over time and response to therapeutic interventions.
- NPI-C [59, 77]: an expanded version of the NPI that includes agitation and aggression domains.
- CSDD [78]: a depression symptom-severity measure specific to dementia, also capturing mood symptoms.
- BI [79]: assesses functional ability in older adults with dementia, focusing on 10 physical domains of basic ADLs [80].
- SIB-8: a cognitive assessment tool [52] for moderate to severe dementia [40, 41, 46, 48, 50, 81, 82].
- MMSE [83]: assesses global cognitive function and dementia severity.
- Bush-Francis catatonia rating scale (BFCRS): screens and rates emergent catatonia or catatonic symptoms [62].
- CAM [55, 84–86] and Family-CAM (FAM-CAM) [87]: used to identify and monitor delirium.

### Statistical analyses

In the original design, we planned to employ various analyses sets including Intent-to-Treat (ITT), Per Protocol (PP), Completer, and Safety samples. The primary focus was on the ITT sample, considering all randomized participants regardless of adherence or dropouts. Separate analyses comparing ITT and PP/Completer samples were also planned to assess the impact of non-adherence and dropouts.

#### Descriptive and preliminary analyses

Baseline characteristics between intervention arms were to be compared using pooled t-tests, or Wilcoxon Rank-Sum tests as nonparametric alternatives, for continuous variables and chi-square tests or Fisher’s Exact Tests for categorical variables. This step also included an examination of the demographics and clinical variables of non-completers.

#### Longitudinal data analysis

For analyzing longitudinal data, methods for allowing missing data under the assumption of data missing at random (MAR) were proposed [88–90]. Multiple imputation [91] would be used for missing covariate data. Sensitivity analysis was planned to explore the impact of missing not at random (MNAR) data.

#### Adjustments for multiple outcomes

In secondary analyses, a Bonferroni correction was to be used to adjust for multiple outcomes; unadjusted and adjusted p-values would be reported. Site pooling rules [89] were planned for implementation if necessary, along with sensitivity analyses for pooled data.

#### Generalized linear models for inferential analyses

A longitudinal generalized linear models (GLM) approach was selected for inferential analyses [88, 89, 92]. This approach accommodates missing data, correlation among repeated measurements, and a wide range of distributional assumptions for outcome variables [88].

### Specific analyses for study aims

#### Aim 1 (efficacy) analyses

The primary efficacy analysis aimed to compare ECT and S-ECT arms with regard to level of severe agitation using the CMAI total score. The primary model would include factors for intervention, time, intervention-by-time interaction, site, and baseline CMAI. Differences in adjusted intervention means (intervention effect size) were to be estimated using 95% confidence intervals. Secondary efficacy analyses were to follow a similar procedure using ADCS-CGIC, NPI-C, and PAS.

#### Aim 2 (tolerability and safety) analyses

Differences in the primary tolerability outcome (SIB-8), the primary safety outcome (CAM) and secondary tolerability/safety outcomes (FAM-CAM, BFCRS) were to be estimated using similar methods as in Aim 1, while dropout rates, and adverse event proportions were to be compared using Logistic regression.

#### Exploratory analyses

Exploratory outcomes, including CSDD, QUALID, and BI will be analyzed as described for Aim 1.

### Revised study design: Transition from RCT to open-label study design

Our study began as an RCT with participants being randomly assigned to either ECT or S-ECT, a design intended to provide a high level of evidence through comparison and blinding. However, as the study progressed, we encountered significant challenges that impacted our ability to continue with the RCT format. These included difficulties with recruiting participants and practical hurdles in implementing the S-ECT component.

#### Revised trial design

In response to these challenges, we made a strategic decision to revise the study design to an open-label format. In this revised design, all participants receive the actual ECT treatment. This change eliminated the need for the S-ECT group and, consequently, the process of randomization.

#### Revised study setting

A significant challenge encountered in the original study design was the limited availability of beds in psychiatric units. This was exacerbated by the COVID-19 pandemic particularly at the peak periods that brought unprecedented challenges to healthcare systems worldwide, including the facilities involved in our study. Our clinical sites went through numerous periods of lockdown, enhanced infection control measures that reduced overall hospital capacity, and re-allocation of healthcare resources to address the surge in COVID 19 patients leading to staffing shortages. As a consequence of these pandemic-related pressures, the overflow of patients with dementia and severe agitation to medical floors became more pronounced. Many individuals who would typically be admitted to psychiatric units were instead being treated on medical floors due to acute psychiatric bed shortages. This situation presented a unique challenge to our recruitment efforts, as the intended study population was dispersed across different hospital settings. In response to these extraordinary circumstances, we revised our study to include patients from medical floors. This adaptation was not only a response to the initial challenge of limited psychiatric bed availability but also a necessary measure to navigate the additional complexities brought on by the pandemic. By extending our recruitment to medical floors, we aimed to ensure a continued, adequate participant enrollment process.

#### Revised sample size

The transition from RCT to open-label design had direct implications on the sample size and eliminated the comparison of two distinct treatment groups. The open-label approach focuses on the direct observation of outcomes within a single ECT treatment group, without the need for the S-ECT group. This change simplified the statistical model and consequently the sample size was substantially decreased. With a sample size ranging from 30 to 50, we aim to detect a standardized effect size of 0.53 SD for the primary outcome, the CMAI. This allows for detection of a raw effect size of approximately 6.1 to 12.1 points in CMAI scores with 80% power. The PASS software was used for sample size calculation [93].

#### Revised participant inclusion

The revised eligibility criteria expanded the age range from 55–89 years to 40 years and older to allow our subject sample to be more representative of the diverse age of onset of various types of dementia. Additionally, the revised design allows for the inclusion of participants with slightly lower CMAI scores to accommodate a broader range of agitation severities. The CMAI is one of the study’s primary outcomes for agitation symptoms, and as such, it was crucial to ensure that the CMAI inclusion criterion accurately reflected the agitation in patients being recruited to participate. The new CMAI inclusion criteria require either one item scored as a 5 or more or two items scored as a 4 or more on aggressive or physically nonaggressive but potentially dangerous behaviors. The study site PIs, along with the Data and Safety Monitoring Board (DSMB) and National Institute of Aging (NIA), found that the patients who met this updated agitation inclusion criterion were the targeted population for the study procedures.

#### Revised sample selection

In the revised study design, the sample selection criteria were expanded to include a broader range of dementia types. While the original study only included participants with dementia due to AD, the revised criteria now include participants with vascular dementia with no current or prior stroke history, frontotemporal dementia, or dementia with Lewy Bodies. Given that patients with other forms of dementia can experience severe agitation, making this change was crucial in executing a more well-rounded trial. The core aspects of the original sample selection remain intact in the revised design.

#### Randomization

With the change to open-label format, our study design was revised to remove randomization.

#### Blinding

With the change to open-label format, our study design was revised to remove blinding.

### Revised processes, interventions, and comparisons

In the revised study design, all processes, interventions, and comparisons related to S-ECT were removed, focusing solely on actual ECT treatments. There were no changes to the ECT protocol.

#### Revised schedule of procedures

**Fig 1:**
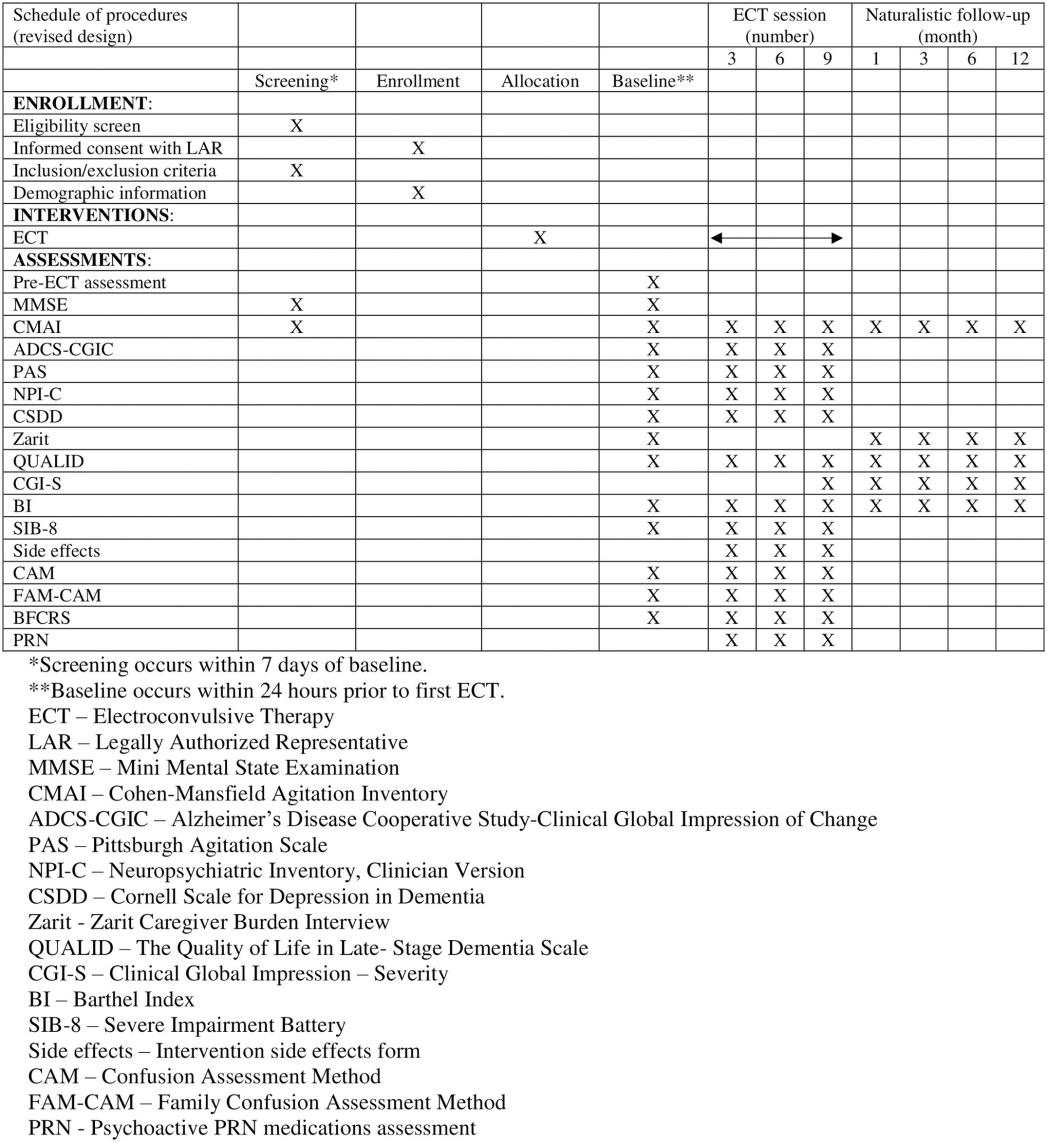
SPIRIT figure.

In the revised study design (Fig 1), the schedule of procedures was simplified by removing all elements related to S-ECT, focusing exclusively on the administration of actual ECT treatments.

#### Outcome measures

Despite the revisions in the study design, the outcome measures remain unchanged.

### Revised statistical analyses

#### Outcome

The primary/secondary outcome variables remain CMAI, ADCS-CGIC, NPI-C, and PAS total scores with assessments made at baseline, after 3rd, 6th, and 9th ECT, or at time of exit prior to 9th ECT. SIB-8 and CAM continue to be the safety/tolerability outcomes. Additional secondary tolerability/safety outcomes are FAM-CAM and BFCRS. In addition, due to the changed design, newly added feasibility outcomes include: recruitment information such as number of patients with dementia referred for ECT, number with LAR, number meeting inclusion/exclusion criteria; number approached; number consented; number of patients providing assent; barriers to patient participation, e.g., reasons why LAR was not approached; LAR’s reasons for refusal to consent for patient participation; and patient retention (e.g., number of dropouts, number of ECT received prior to dropout, reasons for discontinuation).

#### Preliminary descriptive analyses

Univariate descriptive statistics and frequency distributions will be calculated for baseline characteristics to describe the sample. To investigate potential limits on generalizability, we will compare demographic/clinical variables of non-completers with the completer group.

#### Analyses for aim 1 (effect)

The pre to post change scores are determined as the difference between the baseline score and the final score for a given outcome (after either the 9th ECT or at the point of study exit). The magnitude of unadjusted pre to post changes in each outcome will be described using 90% confidence intervals. Paired t-tests (or Wilcoxon Signed Ranks test) will be used to determine if a statistically significant change occurs from pre-treatment to post-treatment. Trajectories of unadjusted changes for each outcome will be described graphically. Post treatment scores and change scores (and corresponding 90% CIs) also will be adjusted for covariables using a longitudinal generalized linear models (GLM) approach with time as the primary independent variable. Adjustment covariables include baseline values for the outcome, site, and diagnostic category. GLM can accommodate correlation among repeated measurements, missing data, and a wide range of distributional assumptions for outcome variables through specification of appropriate link functions.

#### Analyses for aim 2 (tolerability and safety)

SIB-8 is measured daily for continuous safety monitoring; measurement time points for statistical analyses will be the same as for primary/secondary outcomes for consistency. Analyses will follow the procedure described for Aim 1. The primary safety outcome is CAM (presence/absence of confusion). Additional secondary tolerability/safety outcomes are FAM-CAM, BFCRS, adverse events (AE) and serious adverse events (SAE) reported as they occur. Data for CAM is dichotomous and measured daily during the active treatment. Descriptive statistics will be used for primary analyses and will include the number of occurrences of delirium during the ECT treatment course, and the proportion of patients who had to be discontinued from ECT due to delirium. If appropriate, the longitudinal profile of delirium occurrence will be evaluated using GLM, as described for Aim 1.

#### Analyses of feasibility measures

Standard descriptive measures (mean, median, frequency distributions, proportions and accompanying 90% CI) are used for describing feasibility. Examples of feasibility measures include the proportion of patients referred for ECT who have a LAR, proportion of patients with LAR meeting inclusion/exclusion criteria, proportion of LARs approached for study participation, proportion of eligible patients whose LAR consent for the patient to receive ECT, proportion of patients who provide assent for the procedure, proportion of patients who are unable to receive ECT at the first treatment session (proportion who are withdrawn from study prior to receiving first treatment), dropout proportion, number of ECT received prior to dropout, and reasons for discontinuation. If sample size permits, we will explore predictors of dichotomous outcomes (e.g. dropout) using logistic regression.

### Data management and safety monitoring

#### Adverse events reporting

Any AE, SAE or unanticipated problems (UPs) reported by study sites are entered into the WebDCU^™^ database. The data manager reviews these reports for completeness, and upon verification, alerts the Medical Safety Monitors (MSMs). The system also checks whether the boundary of a stopping rule is triggered by an SAE, prompting an immediate review by the DSMB.

#### Data Center safety monitoring responsibilities

The Data Center is responsible for providing statistical safety monitoring reports to the DSMB. These reports include intervention comparisons of safety outcomes, aiding the DSMB in evaluating the balance between patient risk and treatment benefits. The final decision on amending or terminating the study, based on the DSMB recommendation, rests with the NIA.

#### Data and Safety Monitoring Board

The DSMB, approved by the NIA, is tasked with overseeing the safety, data quality, and recruitment aspects of clinical trials, particularly focusing on the use of ECT in our study population. Comprising two psychiatrists specialized in ECT and clinical trials, along with a biostatistician experienced in clinical trial design, the DSMB operates through teleconferences with the NIA program officer to evaluate recruitment processes, data accuracy, and adverse events. Their mandate extends to offering consultative advice to the NIA on trial conduct, with the authority to recommend study termination if adverse effects outweigh potential benefits. Primarily, the DSMB ensures the safety of ECT in treating severe aggression in Alzheimer’s disease patients, reviewing safety outcomes, making recommendations on study procedures, and overseeing protocol and consent form changes. They conduct regular reviews, provide oversight on safety and data flow, and respond to serious and non-serious adverse events, with the ability to directly influence the continuation, amendment, or cessation of the study based on their findings. Additionally, they manage medical monitoring for protocol inquiries and adverse events. DSMB meetings occur quarterly and as needed, structured in open and closed sessions to discuss trial conduct and outcomes comprehensively, culminating in reports that guide the NIA’s final decisions on the future of the study.

#### Single IRB oversight

The single Institutional Review Board (sIRB) reviews events according to Office for Human Research Protections 2007 guidance, focusing on ensuring meaningful reporting of unanticipated problems and adverse events for enhanced protection of human subjects.

#### FDA reporting

All AEs and SAEs are reported to the FDA in accordance with 21CFR812 guidelines. The PIs are responsible for reporting unanticipated adverse device effects to the FDA within 10 business days of discovery. Semi-annual progress and annual reports to the sIRB and FDA include investigator details, patient numbers, safety and efficacy evaluations, summaries of AEs and SAEs, and protocol deviations. A final report of comprehensive study outcomes and risk analysis is submitted within 6 months of study completion or termination.

### Safety considerations

Safety monitoring for subjects undergoing ECT includes physical examinations, screening for any changes to health or ECT side effect, such as delirium (measured by the CAM), headache, nausea, and muscle aches. Cognitive function is closely monitored using the SIB-8 and BI to detect significant cognitive decline.

#### Cognitive safety monitoring

Given the risk of transient cognitive effects associated with ECT, daily cognitive assessments using the SIB-8 are conducted [61, 82, 84]. The CAM is also employed to monitor and manage the risk of delirium [94]. The criteria for an adverse cognitive event vary based on baseline cognitive performance [66]; a decline of >6 points on the SIB-8 [61, 84] or a decline of > 30 points on the BI from baseline [79] is considered significant. Treatment modifications or discontinuation are based on these thresholds, with additional consideration for new-onset delirium and clinical judgment [95, 96].

#### Criteria for discontinuation of acute ECT

Discontinuation of acute ECT is considered in cases of any adverse event of special interest (AESI), including prolonged delirium, significant cognitive decline, serious medical conditions that precludes safe administration of ECT, patient or LAR withdrawal, achievement of remission before completing acute phase, patient objection, and ADCS-CGIC score of 7 (marked global worsening).

#### Overall study safety evaluation

Three AESI are continuously monitored: prolonged delirium, significant decline, and marked global worsening (ADCS-CGIC score of 7). Sequential Probability Ratio Test (SPRT) guidelines inform immediate DSMB reviews to assess risks versus benefits and modifications or termination of the study. Final decisions are made by the NIA based on DSMB recommendations.

The safety considerations in the study were maintained without any changes following the revision of the study design.

### Ethical considerations and declarations

IRB approval and consent procedures: Following NIH policy, we received approval from the Mass General Brigham IRB as the sIRB of record. All consent procedures across participating sites adhere to the guidelines of the sIRB.

#### Informed consent and participant decisional capacity

Given our study participants have advanced dementia and lack the decisional capacity for informed consent, voluntary written informed consent will be obtained from LARs, as per Alzheimer Association guidelines. The LARs, identified according to state law, include healthcare decision-makers, legal guardians, or advance directive holders, or a proxy decision maker by local law such as spouses, adult children, or siblings. Signed and dated copy consent forms are placed in a locked study file kept separate from the subject’s de-identified data.

#### Assessment of assent

**Fig 2:**
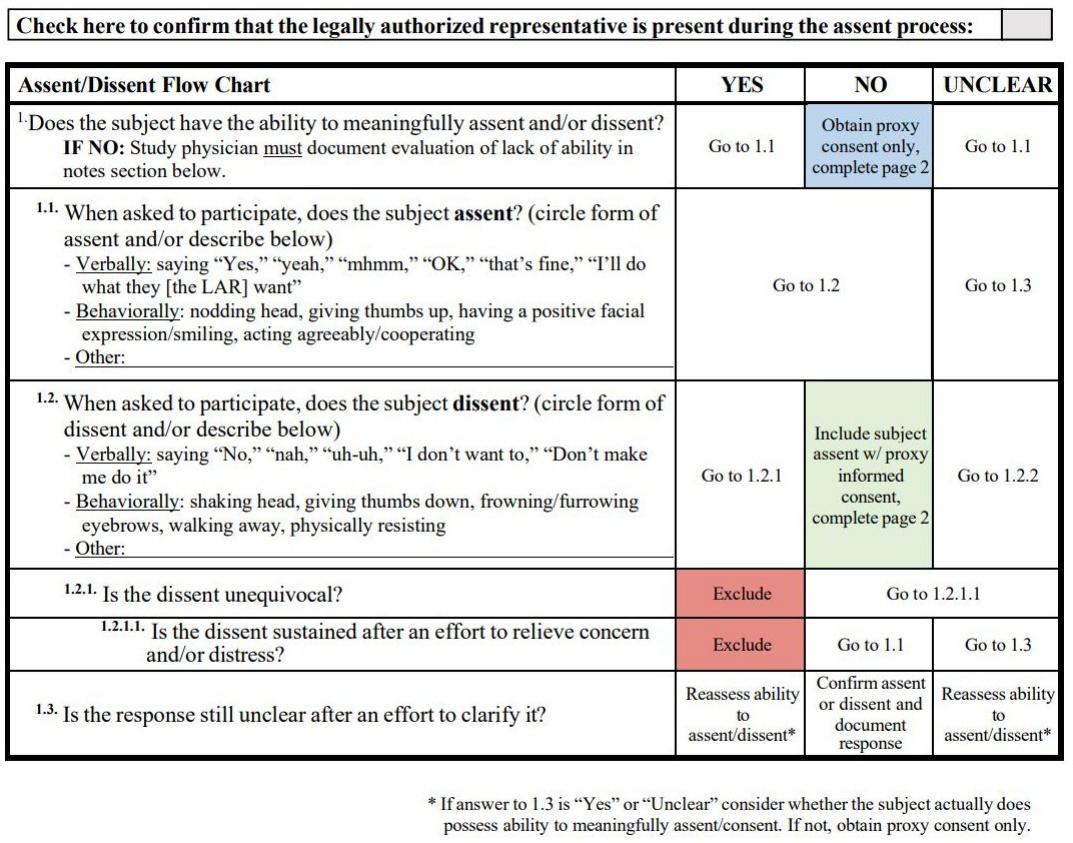
Assent/dissent flow chart.

In instances where participants have the capacity to understand, meaningful assent or dissent will be sought prior to obtaining LAR consent. This assessment will be conducted by the attending physician, who will determine the participant’s ability to provide meaningful assent or dissent. The "Assent/Dissent Flow Chart" (Fig 2) will guide this process, and any dissent will lead to exclusion from the study.

#### Remote consent options during COVID-19

Due to the pandemic, remote informed consent options have been included, utilizing Mass General Brigham IRB 21 CFR Part 11-compliant processes.

#### Consent and assent documentation and withdrawal criteria

All consent and assent processes will be documented according to protocol. Participants showing combative or agitated behavior in the ECT suite will be managed according to standard clinical practice, including calming strategies and PRN medications. Persistent physical resistance or verbal refusal to participate on three consecutive treatment days will be deemed as withdrawal of assent, leading to immediate study withdrawal.

## Results

Enrollment for the single-blind RCT commenced on 01/27/2021, and enrollment under the revised open-label study design started on 07/07/2023. The study is ongoing and open to enrollment. The study is ongoing and open to enrollment.

## Discussion

Despite the major protocol amendments, the core intent of our research has remained the same: to explore the effectiveness and safety of ECT in managing severe treatment-refractory dementia-related agitation. This fundamental goal anchors our study, guiding our revised methodologies while still ensuring that our research continues to address a critical gap in dementia care. The researchers took initiative in developing protocol amendments and implementing study design adjustments, with guidance from the DSMB and the NIA.

Having previously outlined the specific modifications made to our study design, we will review the specific challenges our study faced, delve into the practical difficulties, recruitment barriers, and other unforeseen obstacles we encountered.

### Regulatory challenges

Among the challenges, regulatory hurdles stood out as a significant factor contributing to delays as the study was being launched and later in the study when major protocol amendments were submitted. Our Investigational Device Exemption from the FDA took about 26 months to received approval. During this period, the submission was reviewed three times with iterative changes to the protocol based on feedback from the FDA. Concerns expressed by the FDA included the safety risk of ECT including worsening dementia and the increased risk for delirium related to ECT administration in a vulnerable older population with an existing neurodegenerative illness. Additional inclusion criteria that were required to be added to the protocol were the definition of agitation severity criteria and the number of failed prior pharmacotherapy trials.

Another regulatory challenge we navigated in our multi-site NIA-funded study was the use of sIRB. While designed to streamline ethical review processes for multisite studies, its practical application introduced unique complexities. These included coordinating the harmonization of protocols and consent forms across different study sites, managing diverse local context issues, and ensuring consistent adherence to sIRB decisions. The sIRB model, although beneficial for reducing redundancy in IRB reviews, required significant effort to align the varied administrative, ethical, and operational standards of each participating site.

Understanding these regulatory complexities is crucial, as they highlight the often-overlooked administrative and procedural aspects of clinical research that can have profound effects on study outcomes and timelines.

### Recruitment challenges

Oftentimes, patients have tried multiple treatments before being recommended to receive ECT. In older adults with dementia who have severe agitation, families and caregivers are eager to find an effective treatment to improve the quality of life for their loved one. LARs were reluctant to consent to study participation as randomization to simulated ECT may delay for up to three weeks, potentially clinically effective ECT. Barriers to recruitment also included a limited number of inpatient beds on a geriatric psychiatry unit that are specifically dedicated to individuals with neurocognitive disorders, due to both the financial costs of inpatient dementia care and the pandemic induced barriers to long term care discharge that reduced the overall inpatient turnover rate.

### Operational challenges

A comprehensive review in 2023, examining 19 studies on employing ECT for managing agitation in dementia patients, demonstrated promising outcomes [97]. However, it also underscored several notable deficiencies within the existing body of research, including the lack of randomized controlled trials, ambiguous dementia definitions, reports from single sites, small cohorts, inconsistent ECT methodologies and session counts, the absence of uniform cognitive evaluations, and variability in both the assessment and documentation of adverse reactions. Initially aiming to execute a well-powered randomized clinical trial, the study faced recruitment challenges, leading to a shift towards a single-arm, open-label approach focused on exploring the impact of ECT on severe aggression and agitation among those with dementia. Although transitioning to an open-label study diminishes the robustness of the findings, the multicentric approach of this research, uniform ECT practices, and rigorous tracking of both benefits and side effects considerably bridge the identified research gaps.

## Conclusions

The transition of the ECT-AD study design from an RCT to open-label design exemplifies adaptive research methodologies in response to real-world challenges. Results from both the RCT and open-label single arm trial will provide important insights into the potential efficacy and safety of ECT in managing severe treatment-refractory agitation in those with dementia, potentially influencing future clinical practices and research approaches.

## Supporting information

S1 File Original protocol. (PDF)

S2 File Revised protocol. (PDF)

S3 File IRB approval letter 2020. (PDF)

S4 File IRB approval letter 2021. (PDF)

S5 File IRB approval letter 2022. (PDF)

S6 File IRB approval letter 2023. (PDF)

S7 File Clinical trials registration. (PDF)

S8 File SPIRIT checklist. (PDF)
