# Supplementary material for: Electroconvulsive therapy for the acute management of severe agitation in dementia (ECT-AD): A modified study protocol
Source: PLoS One. 2024 Jun 28;19(6):e0303894. doi: 10.1371/journal.pone.0303894 (PMC11213353; doi:10.1371/journal.pone.0303894)
Supplement: S1 File — (PDF) [file pone.0303894.s001.pdf]

A Randomized Controlled Trial of Electroconvulsive Therapy plus Usual Care versus Simulated-ECT plus Usual Care for the Acute Management of Severe Agitation in Alzheimer's Dementia (ECT-AD).

Version Date: August 24, 2020

DETAILED PROTOCOL

I. BACKGROUND AND SIGNIFICANCE

Definition, Prevalence and Impact of Agitation in Alzheimer's Disease: Alzheimer's disease (AD) is the most prevalent neurodegenerative disease of aging, affecting approximately 5.8 million individuals in the US, and predicted to increase to 13.8 million by 2050[1]. The neuropsychiatric symptoms (NPS) of AD, which include agitation, depression and apathy, increase morbidity and mortality, magnify public health burden of AD[2,3] and adversely impact caregivers. Agitation is the most common NPS[4] and accounts for about 12% of the total health and social care costs (approximately $31 billion/year) for people with dementia[1,5]. This proposal targets agitation in AD due to its high prevalence, importance to caregivers, and implications for safety and quality of life. Agitated behavior is defined as "inappropriate verbal, vocal, or motor activity that is not explained by needs or confusion" and can be characterized as either aggressive or non-aggressive[6,7]. Non-aggressive symptoms of agitation in dementia include restlessness, pacing, wandering, repetitive questioning, chatting, inappropriate disrobing, and verbal outbursts[7]. Aggressive behavioral symptoms of dementia include fighting, throwing, grabbing, destroying items, verbal outbursts, cursing, and screaming[7]. Nearly all individuals with AD develop NPS over the course of the disease[8]. In summary, agitation is implicated in a vicious cycle of negative events including deterioration of family and professional relationships[9], increased risk of death[10], and increased caregiver burden, which is a significant predictor for institutionalization or death of AD patients[11].

Current treatments for severe agitation in AD are lacking: Treatments for severe agitation in people with advanced dementia are urgently needed. Behavioral therapies are recommended as first-line treatments for agitation in AD[12]; however, they require substantial time to take effect and may be less effective for the most severely agitated patients. For these reasons, psychotropic medications, especially antipsychotics, are widely used to treat agitation in AD. However, the efficacy data for antipsychotic medications are inconsistent[13,14] and their use is associated with increased mortality resulting in an FDA black-box warning[15,16]. In comparison, antidepressants (particularly selective serotonin reuptake inhibitors [SSRIs]) have fewer and less severe adverse effects, but treatment may be complicated by cardiac conduction-delaying effects[17]. A multi-center randomized clinical trial of citalopram for agitation in AD (CitAD) demonstrated a reduction in agitation (40% response rate). However, the study was limited to outpatients with less severe dementia and agitation, and the effects were only evident after 6-9 weeks[17]. In light of the limited efficacy and potential hazards of current treatment approaches, new treatments for severe agitation in AD refractory to standard interventions are timely and warranted. We will address this critical need for a new intervention to treat severe agitation in dementia by conducting a single blind, randomized, Simulated-ECT (S-ECT) controlled trial of electroconvulsive therapy (ECT). We will study only inpatients with severe

agitation and moderate to severe dementia, associated with high care costs and poor quality of life, who have already failed prior trials of psychotropic medications[18]. If ECT is found to be safe and effective for severe agitation in AD, the study results could have important public health implications with immediate significant benefits for both patients and families, as well as society at large.

Justification for ECT in management of severe agitation in AD:

ECT Procedure: ECT is the brief application of an electrical pulse to the scalp in order to excite brain cells and cause them to fire in unison, producing a short-lasting seizure under control conditions and general anesthesia[19]**.** ECT has been shown to be very effective across both the lifespan and a range of psychiatric disorders, including depression, schizophrenia, Parkinson's disease, and catatonia. The effects of ECT on multiple neurotransmitter systems[20] (GABA, glutamate, serotonin, norepinephrine, dopamine) and down- regulation of immune activation[21,22] suggest a role for ECT in the attenuation of agitated symptoms in AD, hypothesized to stem from neurotransmitter dysfunction[6,17] and neuroinflammation[23].

ECT safety and efficacy for geriatric mood and psychotic disorders: ECT is the gold standard of neuromodulation procedures in psychiatry with unsurpassed efficacy and remarkable safety in severe mental illness[19]. Randomized controlled trials have demonstrated the safety and efficacy of ECT for the treatment of severe psychiatric disorders of late life, including depression, mania and psychosis[24,25]. The American Psychiatric Association (APA) Task Force for ECT recommends ECT under various clinical situations including the need for a rapid therapeutic response (e.g., life-threatening depression and catatonia), when risks of other therapies outweigh risk of ECT, failure of prior treatments, or patient preference[19,25,26]. Significant evidence supports the efficacy and safety of ECT for older adults with major depressive disorder (MDD) even beyond age 85, with some studies demonstrating greater efficacy of ECT for MDD in older versus younger cohorts[25,27–30]. Furthermore, results from a survival analysis noted that older adults receiving ECT for MDD lived longer and with greater clinical improvement than older adults treated with pharmacotherapy alone[24,25]. Finally, the NIH-funded Prolonging Remission in Depressed Elderly (PRIDE) study demonstrated the safety and efficacy of ultra-brief pulse Right Unilateral ECT (UB-RUL) for older adults with MDD[31–33].

ECT efficacy and safety data for severe agitation in AD: When behavioral interventions and pharmacotherapies have failed, case reports and series have supported the safety and efficacy of ECT for the acute treatment of severe agitation and behavioral symptoms in dementia[34–47]. Our group, including 3 of the 5 sites (McLean, Mayo Clinic, Pine Rest) participating in this proposal, published a multi-site, prospective case series of 23 consecutive inpatients with dementia complicated by severe agitation who did not benefit from behavioral interventions and pharmacotherapy, and were referred for ECT[35]. Eighteen of the 23 subjects with dementia and severe agitation experienced significantly reduced agitation from baseline to discharge as measured by the Cohen Mansfield Agitation Inventory (CMAI)[21,48,49]. Treatment was generally well tolerated by most subjects, but 3 discontinued treatment due to adverse events, and 2 due to recurrence of agitation. Other ECT studies of agitation and aggression in AD also showed positive results. Nine of 11 subjects diagnosed with agitation in AD achieved remission of agitation after ECT[42]. Six of the 9 subjects remained agitation-free with continued ECT treatment for the next year. Further, treatment of agitation with ECT was associated with fewer hospital admissions in the subsequent year. Two other subjects with dementia, who had not responded to pharmacotherapy, also experienced a remission of agitation after treatment

with ECT[38]. In a retrospective chart review study of 16 patients, only two experienced more than transient confusion post-ECT that required treatment, and no other clinically significant adverse events were noted in this population[34]. A recent large retrospective chart review assessed ECT treatment on 60 patients (45 female, 15 male) with agitation and dementia (mean age = 77.5 $\pm$ 8.0 years)[50]. Patients were selected based the following criteria: 1) on admission, the primary psychiatric diagnosis included dementia (any type); 2) the presenting symptoms on admission included behavioral disturbance or agitation associated with dementia; 3) availability of Pittsburgh Agitation Scales (PAS) scores (4 were missing the PAS score but met the first two criteria, and were therefore included). Patients with MDD were excluded and all patients had severe dementia; 28 had unspecified dementia, 22 had AD, 2 had vascular dementia, 2 had frontotemporal dementia, and 6 had mixed dementia. All but three patients were started with ultrabrief right unilateral (UB-RUL) treatment. Eight patients were switched to bifrontal placement due to poor response to suprathreshold ultrabrief right unilateral. The number of treatments in the acute phase ranged from 2 to 10 (median = 6). Seven patients (2 men and 5 women) received more than 12 treatments (maintenance therapy). The Emory protocol was followed, which recommends treating at 6 times initial seizure threshold (IST), with an increase to 8 X IST if no response after the fifth or sixth treatment and 10 X IST if there is no response thereafter with 10 X IST being the maximal dose increment. Patients who were treated with bilateral lead placement were provided with brief pulse width treatments at 1.5 X IST. The baseline PAS total was 9.3 $\pm$ 3.7 and it decreased significantly after three (2.5 $\pm$ 2.8) and six (1.5 $\pm$ 2.3) ECT treatments. No significant ECT related medical complications were observed except transient confusion. A decrease in the number of psychotropics prescribed along with an increase in the GAF score was observed after the ECT treatment course.
Furthermore, the ECT approach described herein is considered more conservative than earlier documented studies, and is well tolerated by this population[32,35]. Across these prior case series, the minimal occurence of serious adverse events is remarkable. Findings from these preliminary studies substantiated the need to study the safety and efficacy of ECT for the acute treatment of severe agitation in dementia using a randomized-control design in a larger, multi-site cohort[46].
Significance of proposal: We propose a single-blinded, randomized, Simulated-ECT (S-ECT) controlled trial to determine the efficacy and safety of ECT for severe agitation in moderate to severe stage AD, while also examining the durability of the acute treatment effect in an exploratory maintenance naturalistic design. This innovative study will fill a gap in the current clinical practice of treating severe agitation in AD using a rigorous methodological approach, and will provide evidence for a new therapeutic application (severe agitation in AD) of a well-studied, established, and safe treatment (ECT). Study findings may demonstrate support for a new therapeutic use of ECT for severe agitation in AD that reduces long-term care placement, decreases the risk of mortality, and enhances patient and caregiver quality-of-life. Such an approach has the potential to offer enormous relief to the substantial socioeconomic burden of behavioral disturbances of dementia.

II. SPECIFIC AIMS

Aim 1: To compare the relative efficacy of up to 9 ECT treatments plus usual care (ECT+UC) versus Simulated ECT plus Usual Care (S-ECT+UC) in reducing severe agitation in 200 participants with moderate to severe AD.

Hypothesis 1: ECT+UC will be more efficacious in reducing severe agitation in AD subjects than S-ECT+UC, as measured by the CMAI total score (primary outcome), and secondary outcomes ADCS-CGIC (Alzheimer's Disease Cooperative Study-Clinical Global Impression of Change Scale)[51,52], NPI-C (Neuropsychiatric Inventory Clinician Scale)[53], and PAS (Pittsburgh Agitation Scale)[54].

Aim 2: To compare the relative tolerability/safety outcomes of ECT+UC versus S-ECT+UC in 200 participants with moderate to severe AD.

*Hypothesis 2: There will be no difference in tolerability/safety outcomes for ECT+UC and S-ECT+UC as measured by cognitive decline (SIB-8), development of delirium (CAM), and serious adverse events***.**

Exploratory Aim: To explore the stability of agitation reduction (CMAI) and global functioning (Clinical Global Impression-Severity [CGI-S]) with assessments at 1, 3 and 6 months following the randomized phase, and then for a fourth visit 12 months after the randomized phase.

Significance: This proposed study represents a novel treatment approach for severe agitation in moderate to severe stage AD and uses a single-blind, Simulated-ECT controlled, randomized design. Establishing safety and efficacy of ECT for severe agitation in AD provides an opportunity to decrease long-term care placement, decrease the risk of mortality, decrease caregiver burden, and enhance quality of life for patients and their caregivers.

III. SUBJECT SELECTION

Subject enrollment is limited to individuals with AD admitted to an inpatient Geriatric Psychiatry Care Unit due to severe agitation that, in the inpatient clinician's judgment, is not responding to pharmacological and behavioral treatment, as defined by at least three failed pharmacological trials to manage behavioral symptoms. A board-certified psychiatrist or neurologist will establish a diagnosis of moderate to severe dementia by using the National Institute of Aging-Alzheimer's Association criteria[55], informant report, and objective testing (Mini Mental Status Exam [MMSE] ≤15). This assessment will further ensure that cognitive and behavioral symptoms are not due to delirium or primary psychiatric disorder. Standard physical examination, urinalysis, and serum chemistries will be collected during the medical screening process. CMAI cut-off scores will be used as the agitation and aggression standard for inclusion (see inclusion criteria below). Finally, all subjects are expected to lack capacity to provide informed consent for ECT and study participation. Therefore, a legally authorized representative will provide informed consent on the subject's behalf in accordance with local state law and hospital guidelines. A detailed description of how assent and consent procedures will be performed can be found below under the "Informed Consent" section.

Inclusion Criteria

1. Diagnosis of Alzheimer's Dementia according to NIA-AA Criteria for dementia[55]
2. MMSE ≤ 15
3. Cohen-Mansfield Agitation Inventory Nursing Home Version (CMAI) score of ≥5 on at least one item of aggression or a physical nonaggressive item that holds potentially

dangerous consequences including hitting (including self), kicking, grabbing onto people, pushing, throwing things, biting, scratching, spitting, hurting self or other, tearing things or destroying property, making physical sexual advances, trying to get to a different place, intentional falling, screaming, making verbal sexual advances, and cursing or verbal aggression (items 1-11, 14, 15, 22-24).

4. At least three failed pharmacological interventions from different drug classes (including antidepressants, antipsychotics, anticonvulsants, prazosin, and cannabinoids) at therapeutic doses (to be determined by clinical judgment) and duration of at least two weeks each to manage behavioral symptoms. These interventions may also include medications discontinued after 1 week due to tolerability concerns. Furthermore, medication trials that occur prior to admission to the hospital may count towards the three failed trials. The trials can be inpatient and/or outpatient. These trials can also be concurrent, such as using two medications from different classes for at least one week at the same time (i.e. polypharmacy).
5. Medically stable for safe administration of ECT verified by standard physical examination, urinalysis and serum chemistries
6. Comprehension of English language
7. Authorized legal representative able and willing to give informed consent
8. Age 55 - 89 years old (inclusive)

Exclusion Criteria

1. Current diagnosis of co-morbid delirium, measured by the Confusion Assessment Method (CAM) and by clinical diagnosis
2. Diagnosis of Non-AD Dementia
3. Lifetime or current diagnosis of Schizophrenia, Bipolar Disorder or Schizoaffective Disorder
4. Active substance use disorder within past 6 months
5. Treatment with ECT or other neurostimulation therapies (e.g., TMS or vagal nerve stimulation) within the past 3 months

Source of Subjects and Recruitment Methods:
Subjects will be recruited from an inpatient Geriatric Psychiatry Care Unit.

IV. SUBJECT ENROLLMENT

Recruitment and Retention Plan
Subjects will be recruited from the inpatient Geriatric Psychiatry Care Unit at McLean Hospital. The unit follows local hospital, university, and state policies with regards to confidentiality and IRB and HIPAA compliance. The decision to refer the patient for ECT through the research study will be made by the inpatient treatment team based on clinical need. A licensed study

physician will obtain informed consent for study participation. Additionally, informed consent for ECT will be collected as part of the standard consenting process for clinical ECT treatment following all hospital policies and state regulations. Recruitment materials and informed consent materials will be used only after approval by the single Institutional Review Board (Mass General Brigham).

Retention of study subjects will be a focus of research study personnel (research assistants, study physicians) in collaboration with the blinded inpatient treatment team. All subjects will be monitored for the development of medical illness and ECT complications (AEs and SAEs) during the entirety of the study. We will make all efforts to retain subjects in the protocol for the entirety of the acute treatment phase including minimizing adverse effects of ECT, specfically reducing frequency of ECT treatments if there are concerns about tolerability, specfically delirium and decline in cognitive status. We expect a total of 210 subjects will be referred by the clinical providers to the study, a total of 200 participants will be randomized and a conservative drop-out rate of 20% will allow for a total of 160 study participants (80 in each arm) to complete the 9 ECT treatments.

Informed Consent. In alignment with updated NIH policy, we have obtained approval from the Mass General Brigham IRB (McLean Hospital) to serve as the single IRB of record for this site with any additional sites delegating authority for review to this single IRB site. No consent procedures at any sites will occur that have not been approved by this single IRB. We will adhere to the crucial ethical principle that research procedures cannot and will not be forced on any participant, and point out that ECT or simulated ECT will not be administered to an unwilling participant.

It is expected that subjects who have the capacity to consent for the study will be outliers of the expected study population given the target severity of Alzheimer's dementia (defined in our inclusion criteria as an MMSE score of $\leq$15 at screening). If the subject has a score greater than 15 on the MMSE, we will assume they retain the capacity to consent and thus exclude them from the study. Voluntary written informed consent is required for participation in this study and will be obtained from study subjects' legally authorized representatives (LARs) by licensed physician investigators with expertise in ECT who are study staff, following the Alzheimer Association guidelines to Investigators[56]. The designation of a LAR is governed by state law. In general, this person will be: a legal guardian, or someone who holds a research advance directive for the patient, or a healthcare agent by advance directive, or a healthcare decision maker by local law such as a spouse, adult child, or sibling. We recognize that the definition of LAR will vary from state to state. At McLean, we will define an LAR as, and seek surrogate consent from, the patient's health care proxy (HCP). The PHRC preferred order of surrogates will be followed, and the Principal Investigator and/or clinician conducting the assent/consent process will clearly document the relationship of the LAR to the subject in the research record. Any assent- and/or consent-related discussions and interviews will take place in the presence of this LAR.

In accordance with local laws, the physician obtaining consent will seek meaningful assent or dissent from the potential participant prior to seeking informed consent from their LAR. In all cases, ability to provide assent or dissent will be assessed in clinical interviews of participants by the attending physician on the inpatient unit who is associated with the research study. The

attending physician will be most familiar with the subject and LAR and therefore will be most able to make a determination on the subject's ability to provide meaningful assent or dissent. In the course of this assessment, the physician will assess the ability of participants to willingly participate in research and go along with the decisions of the LAR (Note: The attending physician may or may not be the same physician who will ultimately obtain consent from the LAR).

Physicians obtaining consent will strictly follow the outline of the "Assent/Dissent Flow Chart[1]" as part of the Documentation of Assent and Consent Process form with input from the attending and will provide detailed and thorough documentation of if and how assent or dissent was obtained on this form. This form will then be filed in the subject chart with the consent form signed by their LAR. If the study physician deems the potential participant unable to provide meaningful assent or dissent, proxy informed consent only will be sought from the participant's LAR. While it is customary to include an assent signed by the participant when the participant lacks capacity to consent, in this study we will make that optional because many participants will be too impaired to give meaningful assent; i.e., they can "sign on the line" but their language skills are too impaired to meaningfully assess their comprehension, even to the limited degree required for assent. Assent should be an affirmative expression or indication of willingness. A person's statement indicating he or she would "go along with" whatever the LAR consents to should be considered an affirmative assent to participate. If the subject can provide assent, but cannot sign the consent form, the study clinician and the LAR will indicate that on the consent form and in the Documentation of Assent and Consent Process Form. Any potential participant who provides clear verbal or behavioral dissent (as described on the Assent/Dissent Flow Chart) will be excluded from the study.

The Documentation of Assent and Consent Process Form confirms the following:
(1) Informed consent form used is most recent IRB-approved iteration.
(2) Participant meets all eligibility requirements.
(3) Participant either:
    a) does not have the ability to meaningfully assent or dissent, OR
    b) has the ability to assent and dissent, AND
        i) has provided either verbal or behavioral confirmation of assent AND
        ii) has not provided any verbal or behavioral indication of dissent to study procedures.
(4) Procedures under item 3 were performed prior to seeking informed consent from the LAR.
(5) The LAR provided informed consent prior to any study specific procedures.
(6) The participant and LAR were given time to review informed consent form (ICF).
(7) ICF was verbally explained by study staff.
(8) All of participant's/LAR's questions were answered to participant's/LAR's satisfaction.
(9) Signed and dated copy of ICF was given to participant and/or LAR.
(10) Signed and dated copy of ICF was placed in a locked study file kept separate from the stubject's de-identified data.

---

[1] **The Assent/Dissent Flow Chart was adapted from "Decision Tree for Respecting Dissent and Seeking Assent for Dementia Research," Black BS, Rabins PV, Sugarman J, Karlawish JH (2010).** ***Am J Geriatric Psychiatry, 18*****(1): 77-85.**

No study procedures will be undertaken or study medications administered until assent from the participant (if able) and informed consent from the LAR is obtained.

If subsequent study sites are added, the protocol will be amended to include an Appendix to describe assent and consent procedures at each site, according to local and federal laws. All subsequent sites will provide written documentation of their local guidelines on consent for adults with impaired decision-making capacity, including (1) a standard for enrollment of individuals with impaired decision-making capacity in research trials involving greater than minimal risk and (2) a standard for consenting for routine clinical ECT. Of note, both standards would need to be met for the research study. This information will be documented in the Appendix.

Given the intended study population, we expect that subjects may be combative and agitated in the ECT suite. ECT staff will treat a combative or agitated patient as they would in usual clinical practice. This includes calming the patient down by sitting with him or her and/or administering an approved study PRN medication if called for by the ECT clinician or nurse. If a subject still resists treatment after using both of these methods, the subject will not receive study treatment that day. Study treatment will be postponed until the next scheduled treatment date. Three consecutive instances of physical interference by the participant and/or verbalizations that the patient wants to discontinue the research study on three consecutive treatment days will be considered withdrawal of assent and the participant will be immediately withdrawn from the study. Physical interference is defined as physical resistance from the patient that makes the ECT team unable to place an IV or administer ECT. This includes inability to place an IV (due to severity of agitation) or use of physical restraint to hold down the patient. This objective termination criteria applies to patients in both the ECT+UC and S-ECT+UC group.

Study Randomization: 200 participants will be randomized 1:1 to ECT+UC or Simulated-ECT+UC using a web-based covariate adaptive randomization algorithm that can control for serious imbalances in covariates considered to have an impact on outcome (e.g. site, antidepressants and antipsychotics use). At the time of randomization, imbalances in these covariates will be accounted for using a minimal sufficient balance approach[57]. The randomization will be carried out centrally at the Data Center (DC) at Medical University of South Carolina (MUSC) using an internally developed, ECT-AD study specific, web-based clinical trials management system (CTMS) referred to as WebDCU™. Clinical site Study Coordinators (SC) will log into the WebDCU™ study database, enter the eligibility and randomization forms and receive the computer-generated randomization assignment. At the clinical centers, the unblinded SC and site-PI will have passwords that give access to this assignment.

V. STUDY PROCEDURES

Interventions

ECT Methods and Rationale: Prior to randomization into ECT+UC or S-ECT+UC, the following assessments will be conducted for all potential study participants in line with standard care: 1) An ECT-credentialed clinician will assess for clinical suitability for ECT; 2) an anesthesiologist will determine safety for anesthesia and a general internist will perform a thorough medical assessment prior to treatment and further pertinent physical examinations as needed during subsequent treatments[58]. Clinical consent for ECT (in addition to consent for participation in research) will be acquired from the LAR. The ECT devices will be either a MECTA spECTrum 5000Q (MECTA Corporation, Oregon) or Thymatron System IV (Somatics, LLC, Illinois). Standard ECT treatment and anesthesia care will be provided to all subjects receiving ECT[19,59].

Clinical personnel (either inpatient staff or ECT staff) will transport subjects back and forth to the ECT suite. In addition, an unblinded study coordinator will accompany the patient and stay with the patient during the ECT session along with an ECT nurse. The unblinded coordinator will give the unblinded ECT staff a Patient Report Form, containing pertinent rating scale information including most recent CAM, SIB-8, BI, ADCS-CGIC, and CMAI scores, in addition to notes on patient's recent behavior.

A more detailed description of the ECT treatment protocol can be found in the ECT-AD Manual of Operating Procedures. The ECT treatment course will consist of up to 9 ECT sessions, administered 3 times per week or less frequently as directed by the protocol. End of acute treatment phase is defined by 9 treatments, or if the participant achieves remission as defined by a CMAI score of $\leq$2 on aggressive and/or potentially dangerous behaviors (items 1-11, 14, 15, 22-24, as listed in the inclusion criteria), or no longer requires inpatient hospitalization per attending physician judgement (see page 12 for all discontinuation criteria of acute ECT treatment). Stimulus method of delivery will be RUL electrode placement, and ultra-brief (UB) pulse width (0.25-0.37ms). At the first ECT session, seizure threshold (ST) will be determined by titration with the empirical dose titration method[60]and subsequent treatments will be approximately 6 times the ST. Following other NIMH sponsored multicenter ECT studies (PRIDE, U01 MH05549533), stimulus settings will be adjusted as directed by the protocol during the ECT course based on seizure quality and treatment efficacy.

Rationale for ECT treatment protocol: The rationale for choosing a course of 9 treatments for our study is informed by two previous studies of ECT in dementia, which demonstrated significant remission of behavioral symptoms by the 9th ECT treatment[34,35]. In addition, 9 treatments fall within the range of ECT treatments administered in 60 patients included in a retrospective chart review of ECT for agitation in dementia[50].
RUL ECT was selected as the method of ECT administration based on pilot data findings[34,35] and because it approaches the efficacy of bilateral (BL; also referred to as bi-temporal or bi-frontal) ECT (based on analysis from the CORE electrode placement study)[61,62]. Cognitive effects of ECT are of concern in our population despite the fact that these effects are transient[48,63]. We will begin treatment with RUL-UBP ECT to minimize risk of adverse cognitive side effects while maintaining treatment efficacy[32].

If after 6 ECT/S-ECT treatment sessions, there is inadequate clinical response as determined by an ADCS-CGIC score of 4-6 (indicating no change or worse), then the treating ECT psychiatrist will switch to bitemporal electrode placements with brief pulse width (0.5-1.5ms). The literature cited above also indicates that BL is a safe and efficacious approach to treating agitation and/or aggression when there is an inadequate response to RUL ECT[34,50]. This approach prevents the continuation of administering treatments that have been ineffective (including the associated risk of anesthesia) and affords the patients the chance to respond to a potentially more effective form of ECT. An ADCS-CGIC of 7 at any time will trigger discontinuation of ECT/S-ECT sessions for that subject.

Both motor and electroencephalographic (EEG) monitoring will be performed to ensure adequate seizure duration as defined by a motor seizure greater than 15 seconds or as clinically indicated in a geriatric population. Methohexital or other commonly used anesthetic medications will be used for anesthesia induction and succinylcholine or other commonly used muscle relaxants for muscle relaxation. All medications used for ECT and anesthesia procedures will be documented in an IRB approved list of possible ancillary medications. For safety and ethical reasons, ECT may be suspended at any time should the participant exhibit difficulty tolerating or accepting ECT, exhibit physical interference, be objectively terminated, or withdraw consent.

Concomitant medications will be administered for treatment emergent effects (e.g., nausea, headache, blood pressure change) as needed according to standard ECT protocols. The study will document the use of all sedative agents, which will be used as covariates in data analyses. Other medications deemed necessary for safe and effective delivery of ECT or to mitigate treatment emergent effects may be used and will be documented on a paper chart. Copies of this documentation will be kept both in a locked file cabinet in the ECT suite and in the nursing supervisor's office for access when the ECT suite is closed.

PRN medications will also be allowed for participants in both groups to manage acute agitation prior to ECT treatment. These medications are: diazepam (5mg-15mg), gabapentin (100 mg-1200 mg/day), midazolam (2 mg-8 mg/day), lorazepam (0.25 mg – 1 mg/day), trazodone (25 mg/day to 300 mg/day), risperidone (0.25-1 mg/day), quetiapine (12.5-200 mg/day), olanzapine (2.5-10 mg/day), or haloperidol (1mg-5mg). These PRN medications are suggested for participants in either group prior to transport to ECT suite, and may be administered at any point during study enrollment. PRN medication used to manage acute agitation will be recorded in the patient's electronic medical record. If a patient requires medication for behavioral management at the beginning of the ECT session prior to IV placement, the patient will be transported back to the inpatient treatment team to be medicated, as per standard practice. If a patient requires medication for behavioral management after IV placement, the patient will remain in the ECT suite and be treated there, as per standard practice.

The patient's family will be allowed to accompany the patient to the ECT suite and remain in the waiting room and prep room but not the ECT treatment room or recovery room. This will help with acute anxiety and agitation prior to ECT and preserve the blinded status of the patient's ECT treatment.

Post-ictal agitation is a phenomenon that is self-limited in time and may last from a few minutes to an hour following seizure elicitation. Post-ictal agitation occurs in a small proportion of patients, and is usually mild in nature (e.g., psychomotor restlessness). In the more severe cases, patients will attempt to pull off monitoring equipment and intravenous lines and try to get off of the stretcher. In the most extreme instances, the patient may become physically aggressive.

The immediate goal in the management of post-ictal agitation is to keep the patient safely on the stretcher. In patients with mild agitation, supportive management, with reduction of external stimuli and quiet reassurance, should suffice to achieve this goal. ECT staff should remain at the patient's side to assure that the intravenous line is not dislodged.

In more severe cases of post-ictal agitation, pharmacologic intervention will be initiated, based on the list of study PRN medications. Of note, these PRN medications may be administered by a study clinician to either group during enrollment.

Safety Monitoring

Safety monitoring of study subjects receiving ECT will include the following: The ECT treatment team will: 1) conduct pertinent physical examinations as needed during the ECT course; 2) screen subjects at each treatment for any changes to health or emergence of ECT side effects including delirium (as measured by the CAM), headache, nausea, and muscle aches; 3) monitor blood pressure, pulse, pulse oximetry, ECG, and EEG throughout and after the procedure; 4) monitor significant decline in cognition based on a >6 point decline from baseline in the Severe Impairment Battery – 8 (SIB-8) total score with three consecutive treatment attempts as communicated in the Patient Report Form. For participants with a total SIB-8 score of ≤5 at baseline, a significant decline in cognition is based on a decline of ≥30 points on the Barthel Index (BI) of Activities of Daily Living total score compared to baseline. The SIB-8 and BI scales are further detailed in the Assessment Measures section (see pages 18-19).

Cognitive Safety Monitoring at Individual Subject Level

ECT carries a known risk for adverse, albeit transient, effects on cognition. To mitigate these risks, we will closely monitor subjects' cognitive status throughout study participantion using daily assessments. The primary measure we will use to monitor cognitive status is the Severe Impairment Battery-8 (SIB-8). The SIB-8 is a brief, clinician-administered, cognitive assessment measure validated in subjects with advanced dementia[64–66]. It will be administered at baseline and then daily thereafter as part of the Confusion Assessment Method (CAM). The Confusion Assessment Method (CAM) is used to determine the presence of delirium. In a study tracking the 1-year mortality outcomes of older adult patients hospitalized in post-acute care, the groups that had the highest to lowest mortality were those with hypoactive, followed by mixed, and then hyperactive delirium[67]. To mitigate the risks of delirium, we are using the CAM as a safety measure for our study. Specifically, the CAM will be used to screen out patients with active delirium as well as determine whether ECT treatment will be held or altogether discontinued.

In determining cognitive safety monitoring rules, there are two important caveats that must be considered: (1) The extant ECT literature does *not* offer specific or consensus guidelines for

defining what level of cognitive decline constitutes an adverse cognitive event, and (2) a floor effect on cognitive testing is expected in a portion of our study sample, which will make it difficult, if not impossible, to reliably measure ECT-related cognitive decline using the SIB-8. To account for these two issues, we propose an empirically-derived, conditional cognitive safety monitoring approach that will vary depending on whether subjects exhibit baseline floor effects on the SIB-8. This approach will allow us to identify and respond to adverse effects on cognition irrespective of a subject's baseline.

Floor effects on the SIB-8 will be defined as a total score of $\leq$5/16 at the baseline assessment. This value was chosen because it is approximately two standard deviations below the modal SIB-8 score, as noted in a large study sample (N > 1300) of subjects with moderate to severe dementia[58]. Moreover, $\leq$5/16 is the value at which we can no longer detect a reliable change on the SIB-8 using the procedure outlined below.

For subjects who ***do not*** exhibit floor effects on the SIB-8 at baseline, a decline of $\geq$6 points, measured on the day of (but prior to) the next scheduled ECT treatment, will be considered an adverse cognitive event. The 6-point threshold empirically derived from two main sources. First, available longitudinal data on the SIB-8 indicates an expected decline of 0.83 and 2.27 points over 6 and 12 months, respectively, in a comparable patient sample[65,66]. Thus, our definition of an adverse cognitive event should unambiguously exceed this expected decline. Second, we reviewed the literature on evidence-based approaches to measuring reliable change on cognitive testing in individual subjects. A review by Duff (2012) highlights one such approach, the Standard Deviation Index (SDI), that is widely used in clinical neuropsychology settings[68]. The SDI is computed using the following formula:

$$SDI = (TX - T1)/SD1,$$

where *T*1 is a subject's SIB-8 score at baseline, *T*X is any subsequent SIB-8 score, and *SD*1 is the standard deviation of baseline SIB-8 scores in a normative sample[68]. We obtained the normative SD1 value from Schmitt et al (2013), which found an overall SD of 3.4 on baseline SIB-8 scores in patients with moderate-to-severe AD (N=1371)[65]. This equation gives an SDI in the form of a z-score, and Duff (2012) suggests any z-score falling beyond ±1.645 can be considered a "reliable or meaningful change based on normal distribution – i.e., only 5% of cases would fall above or below that point[68]. Using this approach, a decline of $\geq$5.593 points would be needed to obtain an z-score of at least -1.645. Thus, a decline from baseline on $\geq$6 points on the SIB-8 was chosen as our adverse cognitive event criterion, as it corresponds to a reliable or statistically meaningful change, per the SDI approach outlined by Duff[68].

As noted above, the 6-point decline threshold also helped inform how we defined baseline floor effects on the SIB-8, which we have set at $\leq$5/16. Specifically, if a subjects obtains a score of $\leq$5/16 at baseline, it would be impossible to detect a reliable change using the SDI framework described above, as a decline of 6 points would mean they obtained a negative raw score on the SIB-8, which is not possible.

To further mitigate cognitive risk in subjects who exceed the 6-point decline threshold, we will use the following procedures:

1. **Treatment modification**: If the subject exhibits a decline of $\geq$ 6 points from baseline on the SIB-8 on the morning of their next scheduled ECT treatment, then treatment will be held off until the next treatment day. ECT treatment days are held on Monday, Wednesday, and Friday. Therefore, the delay in treatment will last 2-3 days to

allow cognitive side effects to resolve as recommended in the FDA Final Order. Additionally, cognitive decline will be documented as an adverse event.

2. **Treatment Discontinuation**: if the subject exhibits *persistent* decline of ≥6 points from baseline on 3 consecutive ECT treatment days (measured prior to the scheduled treatment), then treatment will be discontinued. We have chosen to use 3 consecutive treatment days as the discontinuation criteria, as opposed to a single time point, to reduce the likelihood of excluding subjects who may be exhibiting normal fluctuations in cognition, which are relatively common in later stage Alzheimer's[69,70].

For subjects who ***do*** exhibit floor effects on the SIB-8 at baseline, we will be unable to reliably measure ECT-related cognitive decline using the SIB-8. Instead, we will use the Barthel Index (BI) as a proxy measure of cognitive function[71]. The BI is a widely-used, informant-rated scale that assesses performance in basic activities of daily living (e.g., feeding, bathing). It will be administered daily with unit nursing staff as informants. The BI is an appropriate alternative to cognitive testing given the robust correlation between cognition and activities of daily living in Alzheimer's disease - a correlation that only becomes stronger with disease progression[72]. Using the BI, which has a scoring range of 0-100, an adverse cognitive event will be defined as a decline of ≥30 points from baseline, measured on the day of (but prior to) the next scheduled ECT treatment. This value was also derived from the SDI method outlined above using a normative SD value of 18.8, which is the SD on the BI in moderate-to-severe AD patients (N=341) with an MMSE score of ≤17/30[71]. To further mitigate cognitive adverse effects in patients who exceed this threshold, the same treatment modification and discontinuation procedures outlined above will be employed.

Criteria for discontinuation of acute ECT treatment:

1) Development of any adverse event of special interest (AESI), including but not limited to:
    a. Prolonged delirium (defined as a positive CAM on three consecutive ECT treatment days). During the randomized phase, a positive CAM is defined as meeting the CAM's sensitivity-based criteria for delirium;
    b. Adverse cognitive event (cognitive decline defined as a ≥6 point reduction compared to baseline on the SIB-8 with 3 consecutive ECT treatment attempts. For subjects who score ≤5/16 on the SIB-8 at baseline, cognitive decline is defined as a ≥30 point reduction on the BI with 3 consecutive ECT treatment attempts.
    c. Serious medical condition that precludes safe administration of ECT treatment based on the judgment of the attending physician, ECT clinician and/or anesthesiologist.
2) Subject or legally authorized representative decision to withdraw from the study;
3) Subject achieves remission prior to acute-phase completion (up to 9 ECT); in this case, the subject will continue with weekly ECT or S-ECT (to correspond with the standard clinical practice of treatment tapering) and will continue to be evaluated at established study time points. Remission is defined as a CMAI score of ≤2 on all individual items of the CMAI inclusion criteria for two consecutive assessment periods.

4) Subject is objectively terminated following three consecutive physical interferences and/or verbalization that the patient wants to discontinue the research study.
5) ADCS-CGIC score of 7 (marked global worsening).

Overall study safety evaluation:
For this trial in a population of patients with moderate to severe AD, we define three adverse events of special interest (AESI) to be continuously monitored using a Sequential Probability Ratio Test (SPRT): (1) presence of prolonged delirium based on positive CAM assessments with three consecutive treatment attempts; (2) significant decline in cognition based on a ≥6 point decline from baseline in SIB-8 total score with three consecutive treatment attempts. For participants with a total SIB-8 score of ≤5 at baseline, a significant decline in cognition is based on a decline of ≥30 points on the BI total score compared to baseline; and (3) global worsening of symptomatology defined as a score of 7 (marked global worsening) on the ADCS-CGIC.
The nonbinding SPRT-generated monitoring rules provide statistical guidelines that trigger an immediate DSMB review (beyond the regularly scheduled meetings). The information is used by the DSMB in conjunction with other relevant data and careful judgment in deciding whether the risk to patients outweighs the benefit of treatment and, hence, whether to recommend pausing and amending or stopping the study prior to the planned total sample size. The final decision to amend or terminate the study is made by the National Institute of Aging, based on the DSMB recommendation. The Monitoring and Quality Assurance of the protocol provides further details on the continuous safety monitoring procedures (see X.G).

Simulated ECT (S-ECT, Comparison Group) Methods and Rationale: Simulated ECT was developed to ensure adequate blinding of the inpatient treatment team and clinical raters. The S-ECT process will include the transfer to the ECT suite of those subjects randomized to the S-ECT+UC group at the same intervals as the ECT+UC group. The S-ECT+UC subjects will remain in the ECT suite for the same average length of time as it would take a particular study site to administer ECT to the ECT treatment participants, so as not to unblind the inpatient staff based on length of time of treatment. Clinical personnel will transport subjects back and forth to the ECT suite. In addition, an unblinded study coordinator will accompany the patient and stay with the patient during the S-ECT session. The unblinded coordinator will give the unblinded ECT staff a Patient Report Form, containing pertinent rating scale information including most recent CAM, SIB-8, BI, ADCS-CGIC, and CMAI scores, in addition to notes on patient's recent behavior prior to treatment. Pre-ECT orders and procedures including medical clearance and fasting procedures will be identical for all subjects. While in the ECT suite, conducting gel will be placed on the scalp of the S-ECT subjects to parallel the ECT procedures in the ECT active group. An IV will be placed to provide PRN medication and fluids as needed. IV placement will also provide a method for study staff to assess physical interference and subject resistance to treatment. Records and notes of the ECT and S-ECT procedures will be kept separately in research charts in a locked cabinet in the ECT suite, while in the medical chart a note will be filed indicating that study procedures were followed. A supervising, unblinded clinician will oversee clinical management of the S-ECT randomized study subject at the ECT suite, including administration of PRN medications per protocol guidelines. An unblinded clinician and/or an unblinded study coordinator will sit with the patient in the ECT suite for 2 hours, or as long as

the standard ECT course takes. Television, magazines, or other entertainment may be provided if available. A separate room may be used if available. If there is not another room available, the staff will sit with the patient in the recovery suite.

Agitation/ behavioral management will be the same for both the ECT and S-ECT groups. PRN medications for behavioral management will be prescribed to both groups by the inpatient staff and will be administered to both groups as needed prior to going to the ECT suite. If PRN medication for behavioral management is administered in the ECT suite, it will be recorded in the patient's electronic medical. If a patient requires medication for behavioral management at the beginning of the S-ECT session, prior to IV placement, the patient will be transported back to the inpatient unit to be treated. If a patient requires medication for behavioral management after IV placement, the patient will remain in the ECT suite to be treated. The patient's family will be allowed to accompany the patient to the ECT suite and remain in the waiting room and prep room but not the ECT treatment room or recovery room. This will help with acute anxiety and agitation prior and preserve the blinded status of the patient's S-ECT+UC status.

Usual Care (UC) Methods and Rationale: Both the ECT-UC and the S-ECT-UC groups will be provided with evidenced-based standard treatment (Usual Care [UC]) for individuals with dementia complicated by behavioral disturbances. We developed a checklist of evidence-based non-pharmacological interventions that standardizes UC for both groups based on the DICE algorithm[12]. Usual care is an individualized approach and includes a combination of the following elements provided in a locked, secure inpatient treatment setting: 1) evaluation and clinical management of all medical co-morbidities that may be associated with behavioral disturbances (e.g., infection, sensory problems); 2) reduction or elimination of all medications with anticholinergic effects that may adversely impact cognitive functioning and exacerbate behavioral symptoms; 3) maximization of environmental supports and non-pharmacological treatments to reduce agitation (including but not limited to 1:1 staffing for activities of daily living (ADL) care in quiet setting and assessing environmental antecedents to behavioral agitation); 4) monitoring of all concomitant psychotropic medications; 5) use of "as needed" medications for acute agitation in both treatment groups; 6) holding administration of medications that interfere with ECT treatment for 12 hours prior to ECT treatment, consistent with standard clinical practice.

Communication of subject safety data

An unblinded study coordinator will be responsible for communication of subject safety data prior to each ECT or S-ECT session. The unblinded coordinator will complete a Patient Report Form to give to the unblinded ECT staff that has rating scale information including most recent CAM, SIB-8 or BI, ADCS-CGIC, and CMAI scores, in addition to notes on patient's recent behavior.

12 Month Follow-Up: Methods and Rationale: To address the issue of sustainability of treatment effect, we included an exploratory aim to assess the stability of agitation reduction (CMAI) and global functioning (CGI-S), as well as caregiver burden (Zarit), quality of life (QUALID), and daily functioning (Barthel Index [BI]). Participants in both study arms will be

asked to return to the enrolling site for study visits at 1, 3, 6, and 12 months after the randomized phase. The end of the randomized phase is defined as after completion of Session 9 treatment and scales, or after early discontinuation due to adverse event or remission. Subjects in both study arms may be eligible to receive active ECT treatment during the naturalistic follow-up phase if clinically indicated – this will be solely determined by the subject's clinical treatment team as part of Usual Care. Follow-up assessments will be collected regardless of whether or not the subject is receiving active ECT in the naturalistic follow-up phase.
The study staff will not participate in visits to the subject's residence for naturalistic follow up visits. If the subject is unable to come to the site to complete naturalistic follow up visits, the staff will try to gather as much information as possible over the phone or secure videoconferencing to complete the scales required at naturalistic follow up visits.

Assessment Measures:
Subject's abillity to complete assessment measures is not a requirement to participate in the study. Some subjects with severe dementia may be unable to answer study questionnaires and may be uncooperative or non-verbal. Questionnaires will be completed to the best of the subject's ability.
Cohen-Mansfield Agitation Inventory (CMAI)[73,74] The CMAI is a widely used measure for the assessment of agitation and aggression in dementia-related clinical trials. The CMAI short-form measure, which was used in our pilot study[35], includes 14 agitated behaviors that cover 4 behavioral domains. The 4 domains include physically aggressive (e.g., hitting), physically non-aggressive (e.g., restlessness), verbally aggressive (e.g., screaming) and verbally non-aggressive (e.g., negativism) behaviors. For the present study, the nursing 29-item CMAI will be used. Inter-rater reliability and psychometric properties of the CMAI are well-established[75,76]. The CMAI will be completed by a rater with two different informants at different time periods. At screening and naturalistic follow up visits, CMAI will be collected with a caregiver informantor inpatient nurse. At baseline and treatments 3, 6, and 9, the CMAI will be collected with an inpatient nurse informant. We will refer to this dual collection method as the "split track" method for other rating scales with a similar two-part informant scheme.
Alzheimer's Disease Cooperative Study-Clinical Global Impression of Change Scale (ADCS-CGIC)[51,52] The ADCS-CGIC was designed to measure clinically relevant change over time. The instrument's reliability and validity were assessed in a prospective trial of AD patients and healthy subjects over a 12-month period, demonstrating good reliability at 1 and 2 months, with 90 and 94% of AD subjects, respectively, rated as having changed not at all or only minimally. Face validity was demonstrated by ratings of untreated AD subjects at both 6 months (56% rated as worsened) and 12 months (81% rated as worsened), whereas only 2% of control subjects showed minimal worsening. As a measure of predictive validity, ADCS-CGIC ratings at 12 months were significantly associated with change on four severity scales[51]. The ADCS-CGIC will be completed at baseline, and treatments 3, 6, and 9 by the attending physician with an inpatient nurse informant.
Zarit Caregiver Burden Interview[77] This 22-item questionnaire is designed as a self-report measure for caregivers to report degrees of burden experienced as a result of providing care to a relative. Scores have been found to significantly positively correlate with behavioral problems in older patients and depression scores in caregivers as measured by the Cornell Scale for Depression in Dementia, while being unrelated to the caregiver's age, gender, marital status,

living situation, and employment status. The Zarit will be completed at screening and all naturalistic follow up visits by a rater with a caregiver informant. The informant for this scale must be a family member. If the caregiver for the patient is nursing staff, this scale cannot be completed at naturalistic follow up visits.

Quality of Life in Late-Stage Dementia Scale (QUALID). This 11-item scale is designed to assess the quality of life of a patient with late-stage dementia. A 5-point scale captures the frequency of each item, with low scores reflecting a higher quality of life. This measure has shown high internal consistency and test-retest reliability, and is not significantly correlated with MMSE score[78]. The QUALID will be completed by a rater with a "split track" method. The QUALID will be completed at screening and naturalistic follow up visits with a caregiver informant. The naturalistic follow up visits can be performed with either a family member or nurse informant. The QUALID will also be completed at baseline and treatments 3, 6, and 9 with an inpatient nurse informant.

Pittsburgh Agitation Scale (PAS)[54] The PAS was developed for both clinical and research settings to measure agitation symptoms over time and to assess the response to therapeutic interventions. The PAS rates agitation severity in four behavioral domains: Aberrant Vocalization, Motor Agitation, Aggressiveness, and Resisting Care. The PAS has been demonstrated to be a reliable and valid instrument[54]. The PAS will be completed at baseline and daily Monday-Friday by a rater with an inpatient nurse informant. The rater will ask the nurse to answer the questions "since his/her shift started."

Neuropsychiatric Inventory, Clinician Version (NPI-C)[53] The NPI-C is an improved version of the original Neuropsychiatric Inventory (NPI)[79]. The NPI-C includes expanded domains and clarifies "Agitation" as including both "Agitation" and "Aggression." The method of data collection allows for use of a "LEAD" standard (longitudinal data, expert rater, all data) to make severity ratings for individual items. The validation study found excellent inter-rater reliability and convergent validity with other agitation instruments (e.g., CMAI)[53]. We will use the NPI-C Agitation, Aggression, and their sum as secondary outcomes. The remaining domains of the assessment will also be administered using the traditional NPI format. The NPI/NPI-C will be completed at baseline and treatments 3, 6, and 9 by a rater with an inpatient nurse informant.

Cornell Scale for Depression in Dementia (CSDD)[80] The CSDD, planned as an exploratory measure, is a depression symptom-severity measure specific to dementia. The CSDD is a psychometrically sound measure to assess depressive symptoms in participants with dementia incorporating input from both the participant and informant. We chose to assess depression in the study as mood symptoms often co-occur with dementia related agitation. The CSDD will be completed at baseline and treatments 3, 6, and 9 by a rater with an inpatient nurse informant.

If during the course of the trial, a patient demonstrates suicidal behavior or dangerous behavior with serious safety risk or risk of physical harm to self or others, the patient will be withdrawn from the protocol. In the case of reported suicidality, the inpatient clinician or nurse will be contacted to assess the patient's risk and ensure appropriate action is taken. Information on suicidal behavior may arise during interview for the CSDD rating.

Barthel Index of Activities of Daily Living (BI)[71] The BI is an assessment of functional ability for older adults. It focuses on physical disability in 10 domains specifically in people with dementia. BI takes 5 minutes to administer and has been widely validated as a tool to assess

basic ADLs in clinical trials targeting moderate to severe dementia[81]. The BI is a planned exploratory outcome measure. The BI will be completed by a rater with a "split track" method. The BI will be completed at screening and naturalistic follow up visits with a caregiver informant. The naturalistic follow up visits can be performed with either a family member or nurse informant. The BI will be completed at baseline and Monday-Friday during the 3 weeks of acute treatment with an inpatient nurse informant.

Cognition: Multiple converging lines of evidence support the use of the Severe Impairment Battery-8 (SIB-8) as our primary cognitive outcome including a literature review[46] of clinical trials in moderate to severe dementia, expert neuropsychological consultation, and pilot data[34,35,40,42,44] from our own team. To ensure adequate completion rates of the SIB-8 (approximately 85%), raters highly trained in the assessment of agitation in severe dementia will be employed and will make a total of 3 attempts to assess cognition at a given time point (within a 48 hour window) if a subject is unable to be assessed at the first pass. **SIB-8:** The SIB is an assessment tool designed for individuals with moderate to severe dementia and is sensitive to change in cognition in drug trials for severe dementia[82]. Given the 20-minute length of the SIB, a number of subjects in our pilot study were unable to complete the assessment measure. Thus, we will administer the SIB-8, an abbreviated, reliable, and valid version of the original SIB[82]. The SIB-8 requires 3 minutes to administer, was designed to optimize the detection of change in cognition over time, and has excellent correlation with SIB and MMSE in individuals with severe dementia[64]. The SIB-8 will be completed at screening, baseline, and daily Monday-Friday by a rater with the patient. Additionally, the SIB-8 will be used to monitor cognitive adverse events and modify treatment as described in the "Safety Monitoring" section above.

MMSE: The MMSE[83] is a widely used instrument to assess global cognitive function and stage severity of dementia and will be used at baseline to quantify stage of dementia. The MMSE will be completed at screening by a rater with the patient.

Catatonia Assessment: The Bush-Francis catatonia rating scale (BFCRS) will be used to screen and rate emergent catatonia or catatonic symptoms over the course of the study at baseline and at each treatment-based rating period. This is a standardized, quantifiable exam for catatonia based on descriptors and motor signs as described in the literature and DSM-IV criteria[84]. The BFCRS will be completed at baseline and treatments 3, 6, and 9 by an attending physician with the patient.

Delirium Assessment: Delirium is a syndrome of acute cognitive and behavioral change caused by a medical illness or medication. Delirium is an important confound for studies of agitation in AD as it is common in dementia with an estimated lifetime prevalence of 56%[85]. Dementia is also the strongest risk factor for post-operative delirium[86]. Standard medical practice is to first exclude delirium as the etiology of agitation in AD. The proper treatment for delirium is to address the underlying medical etiology. We will implement 3 procedures to address this issue. First, all subjects will be admitted to an inpatient Geriatric Psychiatry Care Unit where internists consult daily. As part of routine clinical care, all subjects referred for ECT will have a thorough medical evaluation. This will include a physical exam, laboratory tests, ECG and management of non-psychotropic medications. Given the range of potential medical conditions that one might encounter, it is impossible to specify these medical treatments in advance. Rather, we specify that patients will be stabilized medically (as judged by the internist in collaboration with the attending psychiatrist) prior to randomization. Second, we will exclude patients diagnosed at baseline as meeting criteria for delirium using both the structured

Confusion Assessment Method (CAM) and clinical diagnostic criteria[49]. Third, we will monitor emergent delirium as a safety outcome using the CAM daily. A positive CAM post-ECT will require holding the next ECT treatment.

Hypoactive Delirium: Patients with hypoactive delirium present with lethargy and sedation, respond slowly to questioning, and show little spontaneous movement[87]. The CAM assesses hypoactive delirium with two observational items:

1. Altered level of consciousness: A patient who developed new lethargy or stupor as assessed with the CAM will be considered as having hypoactive delirium.
2. Psychomotor retardation: Any new observed psychomotor retardation as assessed with the CAM will be considered as hypoactive delirium.

If a CAM is positive the morning of a treatment, it will be held that day. The patient will receive treatment at the next possible ECT day provided that the CAM is negative that morning.

CAM[66]: The CAM was developed for both clinical and research settings to identify delirium and has been validated in populations that include dementia[66,85,88]. The CAM, along with a cognitive screening test, the SIB-8, will be administered to all patients by trained research staff at baseline and daily throughout the 3-week study. The CAM rates 9 DSM-5[49] delirium criteria including acute onset and fluctuating course, inattention, disorganized thinking, altered level of consciousness, disorientation, memory impairment, perceptual disturbances, increased or decreased psychomotor activity, and disturbance of the sleep-wake cycle. Only the first 4 of the listed criteria are used for the CAM diagnostic algorithm. The CAM has high reliability (including inter-rater reliability) and validity[66]. The Family-CAM (FAM-CAM[89]) is an informant-based screening instrument designed to be used in conjunction with or confirmed by expert clinicians or trained delirium assessors using the CAM. The FAM-CAM will be completed by family members at the screening visit and by nurse's aides at the baseline visit and study visit treatments 3, 6, and 9 to optimize detection of delirium features by informants. This process will be used to maximize sensitivity for detection of this important adverse effect, which can be fluctuating and easily missed on single assessments. Dr. Inouye (developer of the CAM) and close collaborator Dr. Schmitt will participate as study co-investigators and will be integrally involved in the training and standardization of the delirium raters using training materials, videos, and approaches that have been used in >3000 patients and 15,000 assessments to date. The raters will undergo intensive training at baseline, initial standardization, and then reassessment of inter-rater reliability on a quarterly basis throughout the study. Coding sessions will be held with all raters to answer any coding questions during the study twice weekly. In addition, we are including CAM as an outcome measure of safety (in addition to the SIB-8). The Cognitive Safety Monitoring plan (described on page 12) will ensure that after a positive CAM on treatment day, ECT will be held and after three consecutive positive CAM's, ECT treatment will be discontinued. The CAM will be completed daily Monday-Friday during the randomized phase by a rater with the patient. The FAM-CAM will be completed at screening by a rater with a caregiver informant and at baseline, treatments 3, 6, and 9 by a rater with an inpatient nurse informant. The CAM will be collected in the morning, prior to ECT treatment.

Randomization Questionnaire Given the level of advanced cognitive impairment of our study population, we do not expect subjects to meaningfully understand which treatment group

he or she was randomized to. However, we will ask subjects if they understand their treatment group using a Randomization Questionnaire. This is an exploratory outcome measure of the study. The Randomization Questionnaire will be completed by a rater after treatment 9.

<u>Schedule of Procedures:</u>

Screening Visit

Legally authorized representatives must provide informed consent on behalf of the subject before any screening tests are performed. Subjects will receive a unique identification number that will be used on study-related documents pertaining to the subject. Subject eligibility will be determined during up to 2 screening visits in the 7-day screening window. Screening assessments, detailed in Table 1, will include confirmation of diagnosis, medical history, physical examination, urinalysis (UA), serum chemistries and ECG. Serum chemistries include a Comprehensive Metabolic Panel (CMP), a Complete Blood Count (CBC), and Thyroid Stimulating Hormone (TSH) panel to assess thyroid function. A total of about 20 mL of blood will be drawn. Safety labs and ECG will be performed at screening unless already done within 30 days. In addition, subjects will receive cognitive and behavioral testing including the MMSE, CMAI, BI, Zarit Caregiver Burden Interview, QUALID, SIB-8, CAM and FAM-CAM.

Screen failures are defined as subjects who provide informed consent but are not subsequently randomized. If a subject is considered a screen failure, the reasons for exclusion must be documented in the subject's source documents and on the Screen Failure Log.

Baseline Study Visit and Randomization; Week 0 Visit

Subjects will be randomized on Day 0 after all screening and baseline assessments have been completed and after the Investigator has verified that the subject is eligible based on the Inclusion/Exclusion criteria for the study. Subjects will be randomized to either ECT+UC or S-ECT+UC in a 1:1 ratio. Subjects will have all baseline measurements fulfilled before beginning the study intervention (ECT+UC or S-ECT+UC).

ECT treatments will be delievered at a rate of three times per week, unless otherwise clinically indicated. Between 24 and 36 hours after the third, sixth and ninth ECT treatment, participants will be assessed using the CMAI, ADCS-CGIC, PAS, NPIC, CSDD, QUALID, SIB-8, CAM, BI and the FAM-CAM via patient and inpatient nursing staff interview. Study interviews will be conducted by blinded research study staff using input from inpatient nursing staff as an informant.

Post Treatment 3 (at least 24 hours after the 3rd ECT Treatment)

Data will be collected using the following scales; CMAI, ADCS-CGIC, PAS, NPIC, CSDD, Bush Francis, SIB-8, CAM, BI, and the FAM-CAM via patient and inpatient nurse interview. Data on the use of prn medications and symptom monitoring will be collected at each study visit.

Post Treatment 6 (at least 24 hours after the 6th ECT)

Data will be collected using the following scales; CMAI, ADCS-CGIC, PAS, NPIC, CSDD, Bush Francis, SIB-8, CAM, BI, and the FAM-CAM via patient and inpatient nurse interview. Data on the use of prn medications and symptom monitoring will be collected at each study visit.

Post Treatment 9 (at least 24 hours after the 9th ECT Treatment)

Data will be collected using the following scales; CMAI, ADCS-CGIC, PAS, NPIC, CSDD, Bush Francis, SIB-8, CAM, BI, Randomization Questionnaire, and the FAM-CAM via patient and inpatient nurse interview.

Early Termination Visit (for participants who are terminated from the study but have no completed 9 ECT treatments. Will occur at least 24 hours after final ECT treatment).

Data will be collected using the following scales; CMAI, ADCS-CGIC, PAS, NPIC, CSDD, Bush Francis, SIB-8, CAM, BI, Randomization Questionnaire, and the FAM-CAM via patient and inpatient nurse interview. Data on the use of prn medications and symptom monitoring will be collected at each study visit.

Note, for participants who have Treatment #3, #6, or #9 on a Friday, the study visit will occur on the following Monday.

Safety Monitoring (Ongoing)

Safety monitoring will be performed daily throughout the course of the study via patient chart review and delirium assessment.

Safety parameters will include daily assessment of delirium (Confusion Assessment Method [CAM]), adverse events, serious adverse events, and assessment of cognition with the SIB-8 daily.

1 Month Naturalistic Follow Up

Data will be collected using the following scales; Zarit Caregiver Burden Scale, BI, QUALID, CGI-S, CMAI. Subjects will be evaluated for the occurrence of serious adverse events (SAE).

3 Month Naturalistic Follow Up

Data will be collected using the following scales; Zarit Caregiver Burden Scale, BI, QUALID, CGI-S, CMAI. Subjects will be evaluated for the occurrence of serious adverse events (SAE).

6 Month Naturalistic Follow Up

Data will be collected using the following scales; Zarit Caregiver Burden Scale, BI, QUALID, CGI-S, CMAI. Subjects will be evaluated for the occurrence of serious adverse events (SAE).

12 Month Naturalistic Follow Up

Data will be collected using the following scales; Zarit Caregiver Burden Scale, BI, QUALID, CGI-S, CMAI. Subjects will be evaluated for the occurrence of serious adverse events (SAE).

Table 1. Schedule of Procedures: All baseline safety and efficacy measures will be collected within 24 hours prior to the first baseline ECT. Post-ECT measures will be collected between 24 and 36 hours after ECT treatment. The protocol and consent form will require that subjects remain in the hospital for a minimum of 24 hours after the final ECT treatment to ensure consistent collection of safety and efficacy measures.

**Table 1. Schedule of Procedures**				**ECT/S-ECT Treatment Session**			**Naturalistic Follow-up Phase**			
**ECT+UC versus S-ECT+UC for Agitation in Dementia**		**Screening**	**Baseline**	**3**	**6**	**9**	**1 mo**	**3 mo**	**6 mo**	**12 mo**
Study Schedule	Assessments	7 days maximum between Screening and Day 0	Day 0: Within 24 hours prior to 1st ECT/S-ECT							
Evaluate Ability to Meaningfully Assent/Dissent, Conduct Informed Consent Procedures with LAR		√								
Inclusion/Exclusion Criteria		√								
Demographic Information		√								
Medical Assessment for safe administration of ECT (Physical examination, safety labs)		√								
Assessment of Dementia Severity	Mini Mental State Examination (MMSE)	√								
Primary Efficacy Outcome Measure (Aim 1)	Cohen-Mansfield Agitation Inventory (29 Items)[1]	√	√	√	√	√	√	√	√	√
Secondary Outcome Measures	ADCS-CGIC (16 items)		√	√	√	√				
	Pittsburgh Agitation Scale (PAS) (4 Items)[2]		√	√	√	√				
	Neuropsychiatric Inventory, Clinician Version (NPI-C) (14 Items)		√	√	√	√				
Exploratory Outcome Measures	Cornell Scale for Depression in Dementia (CSDD) (19 Items)		√	√	√	√				
	Zarit Caregiver Burden Interview	√					√	√	√	√
	The Quality of Life in Late-Stage Dementia Scale (QUALID)[1]	√	√	√	√	√	√	√	√	√
	Clinical Global Impression – Severity (CGI-S)					√	√	√	√	√
	Blindedness Assessment					√				
Exploratory Functional Outcome	Barthel Index (BI)[1]	√	√	√	√	√	√	√	√	√
Safety Outcomes (Aim 2)	Severe Impairment Battery (SIB-8) (8 Item)[2]	√	√	√	√	√				

	Intervention Side Effects Form[3]	√	√	√	√	√					
	Confusion Assessment Method (CAM)[2] (4 Item)	√	√	√	√	√					
	FAM-CAM by informant family member/caregiver at screening and by inpatient nurse at baseline, treatments 3, 6, and 9 (11 items)	√	√	√	√	√					
	Bush-Francis Catatonia Rating Scale		√	√	√	√					
	Psychoactive prn Assessment		√	√	√	√					
Safety Labs	CMP, CBC, TSH, UA, ECG	√									

[1] Rating will use a "split track" approach; ratings will be collected with the inpatient nurse as the primary informant at Baseline and after treatments 3, 6, and 9; ratings will be collected with the caregiver as a primary informant at Screening and 1, 3, 6, and 12-month Naturalistic Follow Up Visits.
[2] Rating will be collected daily Monday-Friday at the beginning of the day during acute treatment study period. If the patient is expected to have ECT or S-ECT treatment that day, the rating will be collected prior to treatment.
[3] Form will be completed after every ECT/S-ECT Session.
Safety labs and EKG will be performed at screening unless done within 30 days of screening.

VI. SAMPLE SIZE/POWER ANALYSIS

Eighty participants per group are needed to detect, with 85% power, a *standardized* effect size for continuous outcomes of 0.37 standard deviations (sd), assuming complete data, 3 post-randomization measurement time points, level of significance alpha=0.05, two-tailed comparison, correlation between pairs of measurements within participants (interclass correlation) no larger than 0.50 and autoregressive (AR(1)) covariance structure. The standardized effect size of 0.37 sd applies to the primary and most secondary continuous outcomes for both Aim 1 and Aim 2 (efficacy, Aim 1; tolerability/safety, Aim 2). For the primary outcome (CMAI), the corresponding *raw* effect size that can be detected is 3.4 points, assuming sd=9.1 based on pilot studies conducted by the study PI (Dr. Forester) and Pine Rest site PI (Dr. Nykamp)[35]. For the tolerability outcome, SIB-8 (Aim 2), assuming sd=3.4, the raw effect size that can be detected between the ECT and S-ECT groups is 1.26 points[65].

To account for missing information due to attrition of randomized subjects and the dilution effect of intent-to-treat (ITT) analyses, the sample size is increased by 20% to achieve a final ITT sample size of 100 subjects randomized to each intervention group (N-200). The PASS software (PASS; NCSS statistical software, 2008, Kaysville, UT, USA) was used for the sample size calculation.

The method used for sample size determination is appropriate for a design that delivers interventions to individuals (i.e. not clusters or group intervention modalities) with correlation among repeated measurements on subjects. To achieve this target sample size within the project timeline, each of the 5 clinical sites (McLean Hospital, Zucker Hillside Hospital/Northwell Heath, Mayo Clinic, Pine Rest, and Emory University) is expected to randomize 11 subjects/year over 3.5 years of recruitment. These sites will be added in subsequent amendments.

VII. BIOSTATISTICAL ANALYSIS

Analytic methods:

Analysis sets: The Intent-to-Treat (ITT) sample comprises all randomized patients. The ITT sample will be used for the primary analyses. The Per Protocol (PP) sample comprises patients, as originally allocated, who complete the active intervention protocol, and for whom all required measurements on the primary outcomes are made and for whom there are no major protocol violations. The Completer sample comprises all randomized patients for whom all required measurements on the primary outcomes are made. The Safety sample comprises all randomized patients who received at least one ECT or S-ECT session. Analyses will be carried out separately for ITT and PP/Completer samples to test sensitivity of conclusions to non-adherence/dropouts. If differences are present between the PP/Completer and ITT analysis sets, the characteristics of the analysis populations will be examined to aid in explaining any discrepancies.

Preliminary descriptive analyses: Univariate descriptive statistics and frequency distributions will be calculated for baseline characteristics to describe the sample population and assess similarity of the intervention arms at baseline. Continuous measures will be compared between intervention arms using pooled t-test (or Wilcoxon Rank-Sum test as nonparametric alternative); frequency distributions of categorical variables will be compared using the chi-square test or Fisher's Exact Test. To investigate potential limits on generalizability, we will compare demographic/clinical variables of non-completers with the completer group. These comparisons will be carried out for the total group and within intervention groups using methods described above.

Premature exits (drop-outs) and missing data: For primary analyses of longitudinal data, we will employ longitudinal data methods which allow for missing data under the assumption of data missing at random (MAR)[90–92]. Missing covariate data will be imputed using the multiple imputation method of Little and Rubin, if needed[93]. In addition, we will model the dichotomous outcome, missing/not missing end-of-study score (dropout), using logistic regression, to describe and compare the characteristics of subjects missing and not missing primary outcome endpoints. While the MAR missing data mechanism can be justified in many situations, it is possible that the missing data will be missing not at random (MNAR) for some of the outcome measures (i.e., the probability of missingness is dependent on the missing data even after conditioning on the observed data). While there are several general methods for dealing with MNAR data currently in use (including selection models, pattern mixture models, and shared parameter models), there is no current standard recommended method[91]. We will conduct a sensitivity analysis to explore the impact of different strategies and different distributional

assumptions for MNAR on study conclusions. If one MNAR method has become the accepted standard at the time of final data analyses, this method will be the procedure to be used.

Adjustment for multiple outcomes: For secondary analysis using multiple outcome variables, we will adjust p-values using a Bonferroni correction for multiple outcomes and will report both unadjusted and adjusted p-values. Secondary outcomes and exploratory analyses will be evaluated qualitatively in terms of consistency with primary results and cautiously in terms of claiming statistical significance of results. The sensitivity of the study results to adjustment for multiplicity of outcomes will be evaluated.

Site pooling: Site pooling will be minimized to the extent possible to preserve within-site randomization. If a single site (or small number of sites) underperforms with regard to site recruitment, resulting in extremely small site sample sizes that create problems with model convergence, the following pooling rules will apply: (1) a single small site (less than 5 patients in treatment arm) will be pooled with its nearest geographic neighbor having similar patient demographic characteristics (age, sex, race): (2) a few (>1) small sites will be combined based on similar site demographics such that the pooled sites are at least as large as the smallest stand-alone site and not larger than approximately 2x the largest stand-alone site[91]. In addition, we will conduct sensitivity analyses to compare outcome of pooled analyses and analyses that eliminate the small underperforming site (non stand-alone site creating convergence problems).

General analytic framework for inferential analyses: A longitudinal generalized linear models (GLM) approach will be used as the general analytic framework for inferential analyses for the primary and secondary outcome variables[90,91,94]. GLM accommodates missing data under the assumption of missing at random (MAR), correlation among repeated measurements, and a wide range of distributional assumptions for outcome variables through specification of appropriate link functions. Specifically, the basic baseline adjusted GLM model is (subscripts omitted for simplicity):

$$g^{95} = \beta_0 + \beta_1 \text{ (Intervention)} + \beta_2 \text{ (Time)} + \beta_3 \text{ (InterventionXTime)} + \beta_4 \text{ (Baseline Y)} \qquad (1)$$

where g is a monotone link function (identity for Gaussian, logit for binary and log for count outcomes). The distributions of the outcome variables will be assessed prior to breaking the partial blind to determine the appropriate link functions, dsitributional family (e.g., Gaussian, binomial, Poisson), and possible additional transformations that may be needed. The correlation among repeated measures within individuals will be taken into account through specification of residual (R-side) variance-covariance matrix. The variance-covariance of repeated observations within subjects is taken into account by fitting an unstructured (UN) covariance pattern to the correlated (R-side) errors. If the UN covariance fails to converge, other covariance structures (e.g., heterogenous Toeplitz, AR(1)) will be evaluated with the choice among covariance pattern models based on comparison of maximized likelihoods as described by Fitzmaurice, Laird, and Ware[90].

Statistical Analyses for Aim 1 (Efficacy):

Primary Efficacy Analyses: The primary aim is to compare, over the active (randomized) intervention period, the ECT and S-ECT arms with regard to level of severe agitation in inpatients with moderate to severe AD. The primary efficacy outcome variable is CMAI total score. Assessments are made at baseline, after 3rd ECT/S-ECT (Time 1), after 6th ECT/S-ECT (Time 2), after 9th ECT/S-ECT (Time 3); or at time of exit prior to 9th ECT/S-ECT (Time 1, 2, or 3).

The primary (basic) model will use longitudinal GLM (as described above) with the identity link function to model the longitudinal measurements of CMAI at post-baseline time points. The primary time point for comparing intervention means is at the completion of the randomized phase, i.e. after the 9th ET/S-ECT (Time 3) *or* at the point of study exit. The primary model includes fixed effects with categorical factors for intervention, time, intervention-by-time interaction terms as primary independent variables with site (5 sites, fixed effect) and baseline CMAI (continuous) as adjustment covariables. The interaction term (intervention-by-time) will remain in the model regardless of significance. Specifically, the primary GLM model (subscripts omitted for simplicity) for **Y**=CMAI is:

$$E[\mathbf{Y}] = \beta_0 + \beta_1 \text{(Intervention)} + \beta_2 \text{(Time)} + \beta_3 \text{(InterventionXTime)} + \beta_4 \text{(Baseline Y)} + \beta_5 \text{(Site)} \quad (2)$$

The contrast using the appropriate interaction term from the primary model will be used to compare covariate-adjusted intervention means (least squares means, LSM) at the end of the active intervention period between the ECT and S-ECT groups. The difference in covariate-adjusted intervention means (intervention effect size) at the end of the active intervention period ($\Delta = LSM_{ECT} - LSM_{S\text{-}ECT}$ at final time point) will be estimated using 95% confidence intervals. The test of ($H_{0:}$ $\Delta=0$) will use a two-tailed level of significance alpha=0.05, and corresponding p-values ≤ 0.05 will indicate statistical significance of the hypothesis test. We will also estimate with within intervention and between intervention change from baseline for CMAI using 95% CI.

In additional analyses of the primary outcome variable (CMAI), the dichotomous stratification variable (psychiatric medication use: 0/1) will be added to the basic model [Model (1) above] as an adjustment covariable to evaluate the effect of the adjustment on results of primary analyses. If results and corresponding qualitative conclusions regarding relationship between CMAI and intervention are not appreciably different than those using model (1), the term will not be retained. In further analyses, evaluation of: (1) homogeneity of intervention effects across clinical sites (i.e. poolability analyses) and (2) effect modification by use of psychiatric medication will be carried out as described in section "Additional Efficacy Analyses" below.

Secondary Efficacy Analyses for Aim 1: Secondary efficacy outcome varibales for Aim 1 are ADCS-CGIC, NPI-C, and PAS, assessed at baseline and after 3rd, 6th, and 9th ECT/S-ECT *or* at time of study exit. The distributions of the secondary outcome variables will be assessed prior to breaking the blind to determine the appropriate link functions and possible additional transformations that may be needed. Each of the secondary efficacy variables will first be used

separately as the dependent (outcome) variable (**Y**) in the GLM following the procedure described for the primary outcome (CMAI). The primary hypotheses for comparing baseline adjusted post intervention least squares means between intervention groups for each secondary outcome variable will be tested using appropriate model contrasts as described for the primary outcome (CMAI); estimation of effect size (Δ) will follow that described for CMAI.

We will take the multiplicity of secondary outcomes into account using a Bonferroni adjustment with both the unadjusted and multiplicity-adjusted p-values reported.

Additional Efficacy Analyses:

Effect modification of site: In separate secondary poolability analyses, we will evaluate homogeneity of intervention effect across sites by: (1) considering the graphical display of the outcome means over time stratified by clinical site[96]; (2) adding a site-by-intervention interaction term to the basic model as:

$E[Y] = \beta_0 + \beta_1 (Intervention + \beta_2 (Time) + \beta_3 (InterventionXTime) + \beta_4 (Baseline\ Y) + \beta_5 (Site) + \beta_6 (InterventionXSite)$ *(3)*

If site effects are indicated (e.g., InterventionXSite interaction term significant at $p<0.10$[97]), we will: (1) attempt to find explanations based on other features such as trial conduct and characteristics of site-specific study populations; (2) retain site-by-intervention interaction in the analysis model (as recommended by ICH E9[98]) and report results with/without site interaction term; and (3) investigate presence of clinical site outliers and conduct sensitivity analysis with/without inclusion of this outlier. In addition, if sample size permits, descriptive results stratified by site will be presented.

Effect modification of psychiatric medication use: An intervention-by-medication use interaction term will be added to the basic models to explore whether the dichotomous stratification variable, psychiatric medication use, modifies the effect of the intervention on the primary outcome.

$$E[Y] = \beta_0 + \beta_1 \text{(Intervention)} + \beta_2 \text{(Time)} + \beta_3 \text{(InterventionXTime)} + \beta_4 \text{(Baseline Y)} + \beta_5 \text{(Site)} + \beta_6 \text{(Psych med use)} + \beta_6 \text{(InterventionXPsych med use)} \quad (4)$$

The significance of interaction term (InteractionXPsych med use) will be tested using a level of significance of 0.10. These results will be considered exploratory and will be reported in addition to original models in a series of the sensitivity analyses. In addition, if a strong interaction effect is found and if sample size permits, descriptive stratified results will be presented.

Sex-specific differences: Possible moderation of intervention effects by sex in analyses will be explored through inclusion of sex-by-intervention interaction terms in the GLM. The study is not powered for confirming hypotheses regarding moderating effects of sex on intervention, therefore these analyses will be considered hypothesis generating and descriptive. We will report descriptive statistics separately by sex, per new NIH guidelines, if sample size permits.

ECT group-specific analyses: Descriptive statistics (mean, median, frequency distributions, 95% confidence intervals) will be used to describe ECT group-specific outcomes of interest, e.g., (1) average number of ECT received among subgroups, i.e., those for whom agitation/aggression resolves within $\leq$9 ECT; those for whole agitation/aggression has not resolved after receiving 9 ECT, and among those who discontinue prior to receiving 9 ECT and whose agitation/aggresstion has not resolved (i.e., dropouts); (2) frequency of delayed treatment (i.e., treatment held temporarily based on occurrence of adverse events.

Analyses for Aim 2 (Tolerability and Safety)

The safety analyses described in this section are *not intended for continuous AE/SAE monitoring for safety* but rather describe methods to test tolerability/safety hypotheses at the end of the trials. A continuous safety monitoring plan for *adverse events of special interest* (AESI) is described in a separate section below.

Primary Tolerability Analyses (Aim 2): The primary tolerability outcome is the Severe Impairment Battery (SIB-8) measured during the active intervention phase at screening, baseline, and after the 3rd, 6th, and 9th ECT/S-ECT or at time of study exit (SIB-8 is measured daily for continuous safety monitoring; measurement time points for hypothesis testing will be as listed above for consistency with other outcomes). The primary time point is at the end of the randomized phase. Analyses for SIB-8 will follow the procedure described for the efficacy outcomes (Aim 1). An unstructured covariance pattern will be used to estimate the R-side random effect (variance-covariance of within-subject repeated measures). If the model does not converge, different covariance patterns will be assessed beginning with a heterogenous Toeplitz[91].

Primary Safety Analyses (Aim 2): The primary safety outcome is the *Confusion Assessment Method* (CAM). Data for CAM is dichotomous (presence/absence of confusion) and measured daily during the active intervention phase; for hypothesis testing, data obtained at baseline, and after the 3rd, 6th, and 9th ECT/S-ECT or at time of study exit will be used. GLM analyses will use the logit link function and the binomial distribution. An unstructured covariance pattern will be used to estimate the R-side ransom effect (variance-covariance of within-subject repeated measures). If the model does not converge, different covariance patterns will be assessed beginning with a heterogenous Toeplitz[91].

Secondary Tolerability/Safety Analyses (Aim 2): Additional secondary tolerability/safety outcomes are; (1) FAM-CAM and Bush-Francis Catatonia Rating Scale measured at baseline, and after the 3rd, 6th, and 9th ECT/S-ECT or at time of study exit during active randomized phase; (2) discontinuation proportion due to delirium and dropout proportion reported at the end of the randomized phase; and (3) adverse events (AE, SAE) reported as the occur. The GLM modeling approach as described above for the longitudinal outcomes (FAM-CAM [dichotomous], Bush-Francis Catatonia Rating Scale [continuous]) will be used to evaluate the relationship between the secondary tolerability/safety outcomes and intervention status. Each of the secondary tolerability/safety variables will be used separately as the dependent variable in the GLM. For the single time point dichotomous outcomes (dropout, occurrence of AE/SAE),

logistic regression analysis (special case of GLM) will be used to compare proportions between intervention groups. 95% CI for single proportions will be used to estimate the within group proportion; the magnitude of the difference in proportions between the ECT and S-ECT groups (effect size) will be estimated using 95% CI for differences in two proportions. We will explore predictors of dropout using a logistic regression model and evaluate whether dropout predictors are different for ECT and S-ECT through inclusion of a predictor by intervention interaction term.

For adverse events (AEs) and serious adverse events (SAEs) reporting, Medical Dictionary for Regulatory Activities Terminology (MedDRA) will be used to code and classify AE/SAEs appropriately[99]. Frequency distributions of AE/SAEs will be summarized by the AE/SAEs code (as provided on the AE case report form) in terms of frequency of the event, number (proportion) of subjects having the event, severity, and relatedness to the study intervention. Continuous safety monitoring procedures for AE/SAE will be described in a later section.

Analyses for Exploratory Aim:

Randomized Phase: Exploratory outcomes assessed during the randomized phase are: Cornell Scale for Depression in Dementia (CSDD), Quality of Life in Late-Stage Dementia Scale (QUALID), and Barthel Index of Activities of Daily Living (BI) assessed at screening, baseline, and after the 3rd, 6th, and 9th ECT/S-ECT or at time of study exit. Exploratory outcomes assessed during the randomized phase (CSDD, QUALID, BI) will be analyzed as described as described for Aim 1.

Naturalistic (Follow-Up) Phase: Because the study does not attempt to control any clinical behavior during the follow-up phase and patients can move back and forth between original intervention arms or receive other clinically indicated interventions, the follow-up phase is observational and all analyses are considered exploratory (not hypothesis confirming). Outcomes for the follow-up phase are: Cohen-Mansfield Agitation Inventory (CMAI), Clinical Global Impression-Severity (CGI-S), Zarit Caregiver Burden Interview (ZARIT), Quality of Life in Late-Stage Dementia Scale (QUALID), Barthel Index (BI), all measured at baseline-2 and months 1, 3, 6, and 12 during the 12-month naturalistic (follow-up) phase. The baseline for the follow-up phase (baseline-2) for CMAI is the final score for the randomized phase; baseline-2 for QUALID is assessed at the beginning of the follow-up by the caregiver; baseline-2 for CGI-S is obtained at the beginning of the follow-up and baseline-2 for ZARIT is the caregiver assessment at screening (prior to randomization). Each outcome will be described using standard descriptive and graphical and tabular method (where appropriate). The magnitude of the change from baseline to each time point in the follow-up will be described using 95% CI. Frequency distributions will describe clinical events, including changes in intervention, re-hospitalizations, care facility admissions, death.

Analyses of Feasibility Outcomes: Feasibility outcomes include the proportion of eligible patients whose LAR consent for the patient to receive ECT, proportion of patients who provide assent for the procedure, proportion of patients who are unable to receive ECT at the first treatment session (proportion who are withdrawn from study prior to receiving first treatment),

number of ECT received prior to dropout, intervention dropout proportion and reasons for discontinuation. Standard descriptive measures (mean, median, frequency distributions, proportions and accompanying 95% CI) for describing feasibility will be used.

VIII. RISKS AND DISCOMFORTS

A. Vulnerable Population. The vulnerable population in this study includes cognitively impaired individuals. This population must be included because the proposal's aim is to study the efficacy and safety of ECT for severe agitation that occurs in the context of Alzheimer's Dementia.

B. Potential Risks of Adverse Events and Serious Adverse Events. An adverse event (AE) is defined as any change whether undesired, noxious, or pathological in a subject illustrated by signs or symptoms that occur during study participation whether or not it is related to study procedures. A serious adverse event (SAE) is defined as when the event is life threatening or causes hospitalization, disability, or death. The potential risks of AEs and SAEs specifically associated with this study protocol may be related to the clinical diagnostic interview, behavioral assessments, blood collection, neurocognitive evaluation and ECT.

C. Risks associated with clinical diagnostic interview and behavioral measures. The risks include psychological distress, fatigue, inconvenience, and loss of confidentiality. These risks are not beyond what is expected for routine clinical psychiatric evaluation.

D. Risks associated with neurocognitive evaluation.
The risks include performance anxiety, distress, fatigue, inconvenience, and loss of confidentiality. These risks are not beyond what is expected for a routine neurocognitive evaluation.

E. Risks of ECT. ECT is a routine, standard neurotherapeutic intervention that is widely used for the treatment of depression and other psychiatric disorders in younger and older adults. Our use of ECT in a population with dementia and severe agitation is unique and promising. Our highly experienced ECT staff at all five study sites follows treatment guidelines as set forth by the American Psychiatric Association[19]. The potential risks of ECT include the risks of anesthesia (including the very remote possibility of mortality), the possibility of headache (45%), nausea (25%) and general systemic complications, such as dizziness, appetite loss and muscle aches[19]. Post-ictal agitation can occur in up to 7-10% of patients receiving ECT[100]. These risks are temporary and will be managed by the medical staff on the inpatient unit. Serious medical complications are very rare and include death, prolonged seizure, heart attack, stroke , trouble breathing, bone fractures, and mouth problems such as loosening of teeth or other dental fixtures or a cut lip or tongue. The most common adverse effect of ECT is neurocognitive (e.g., memory impairment), which tends to be transient. Subjects and authorized HCPs providing consent will be provided a detailed description of the risks associated with ECT. In addition to the consent form, we have prepared a subject information pamphlet outlining the benefits and risks of ECT and also documenting and explaining the clinical data collected thus far that supports the safety and efficacy of ECT for agitation in patients with dementia. These risks are not beyond what is expected for routine, clinical ECT. Right unilateral electrode (RUL) placement and ultra-brief pulse width are used in order to decrease the extent of adverse effects, particularly neurocognitive adverse effects, while maximizing therapeutic efficacy.

F. Emergent Delirium During ECT. Delirium is common in older adults with dementia. It is a syndrome of acute cognitive and behavioral change and can be caused by a medical illness, medication, or treatment. The development of delirium during ECT was noted during our pilot studies. The CAM (Confusion Assessment Method) will be used to detect delirium, including hypoactive delirium, and will be collected daily. The Family-CAM (FAM-CAM) will also be used to heighten the sensitivity of detecting delirium. Routine clinical assessment will also be used to monitor for emergent delirium in this study. The SIB-8 will be collected at baseline and daily during the 3 weeks of acute treatment.
The CAM was developed for both clinical and research settings to identify delirium and has been validated in populations that include dementia[66,85,88]. A positive CAM on the day of ECT treatment will require holding ECT. After 3 consecutive positive CAMs on ECT treatment days, ECT will be discontinued.
The Family-CAM (FAM-CAM)[89], an informant-based screening instrument designed to be used in conjunction with or confirmed by expert clinicians or trained delirium assessors using the CAM, will be completed by family members at the screening visit and by nurses' aides at the baseline visit and weeks 1, 2, and 3 to heighten detection of delirium features by informants. This process will be used to heighten sensitivity for this important adverse effect, which can be fluctuating and easily missed on single assessments. Dr. Inouye (CAM developer) and close collaborator Dr. Schmitt will participate as study co-investigators and will be integrally involved in the training and standardization of the delirium raters using training materials, videos, and approaches that have been used in >3000 patients and 15,000 assessments to date. Regular coding sessions will be held with all raters to answer any coding questions during the study.

All participants will have routine medical monitoring on an inpatient psychiatry unit including observation of mental and neurological status changes by nursing staff, and vital signs at least twice daily. Significant changes in mental and neurologic status and/or vital signs will be clinically managed by the clinical staff of the units. Additionally, the study staff will be informed.

All adverse events occurring after randomization during the acute treatment phase, will be recorded. During the maintenance phase, only SAEs will be recorded. A list of common side effects of ECT will be used to monitor for adverse events. At scheduled visits, participants and the inpatient clinical staff will be interviewed about whether the participant experienced any symptoms or side effects on the list since the prior visit. If adverse events are noted, they will be rated on a graded scale (Grade 1- Grade 5) based on their clinical severity and frequency.

Grade 1 - Mild; asymptomatic or mild symptoms; clinical or diagnostic observations only; intervention not indicated.
Grade 2 - Moderate; minimal, local or noninvasive intervention indicated; limiting age-appropriate instrumental Activities of Daily Living.
Grade 3 - Severe or medically significant but not immediately life-threatening; hospitalization or prolongation of hospitalization indicated;disabling; limiting self care Activities of Daily Living.
Grade 4 - Life-threatening consequences; urgent intervention indicated.
Grade 5 - Death related to AE.

Medical transfers to outside general hospitals will be monitored. The PIs and designated study physicians will be responsible for monitoring the safety of participants. They will be responsible for appropriate medical care of participants during the study in connection with study procedures. Safety assessments will include physical exams, vital signs, ECGs, monitoring of adverse events, and monitoring and maintenance of concurrent medication records. AEs will be reported to the single IRB per IRB policy. Serious adverse events (SAEs) will be defined per FDA guidelines and reported in a timely manner as is standard in clinical trials (the initial report within 48 hours of becoming aware of the SAE).
The major function of assessing cognition in this proposal is as a safety evaluation, to determine whether ECT adversely affects cognition. We will use the Severe Impairment Battery-8 item Form (SIB-8) (targeted for advanced dementia) and the Confusion Assessment Method (CAM). We assess moderate to severe dementia at baseline based on the NIA-AA criteria[55] (using the Mini Mental Status Examination [MMSE]). Our pilot data and a literature review of clinical trials in moderate to severe dementia[46] support the use of the Severe Impairment Battery-8 (SIB-8) as our primary cognitive outcome[66]. To further ensure adequate completion rates of objective cognitive testing, we will have raters make a total of 3 attempts to assess cognition at a given time point if a subject is unable to be assessed at first pass.
G. Risk of receiving Simulated-ECT. The risk of receiving simulated-ECT is that patients would not benefit from the potential mood stabilizing and agitation relieving effects of ECT, and may continue to experience agitation on the inpatient unit and the ECT suite. If a patient is randomized to S-ECT but experiences marked global worsening as defined by a score of 7 on the ADCS-CGIC, the subject will be automatically terminated from study participation.

Attribution of Adverse Events.

**Unrelated**	The temporal relationship between treatment exposure and the adverse event is unreasonable or incompatible and/or adverse event is clearly due to extraneous causes (e.g., underlying disease, environment)
**Unlikely**	**Must have both of the following** 2 conditions, but may have reasonable or only tenuous temporal relationship to intervention.
	1. Could readily have been produced by the subject's clinical state, or environmental or other interventions.<br>2. Does not follow known pattern of response to intervention.
**Reasonable Possibility**	**Must have at least 2** of the following 3 conditions
	1. Has a reasonable temporal relationship to intervention.<br>2. Could not readily have been produced by the subject's clinical state or environmental or other interventions.<br>3. Follows a known pattern of response to intervention.
	**Must have all 3** of the following conditions

**Definitely**	1. Has a reasonable temporal relationship to intervention.<br>2. Could not possibly have been produced by the subject's clinical state or have been due to environmental or other interventions.<br>3. Follows a known pattern of response to intervention.

Plan for Grading Adverse Events.

Adverse Event (AE). An adverse event (AE) is any adverse change from the participant's baseline condition, regardless of relationship to study intervention, including clinical or laboratory tests, or abnormalities which occur after informed consent is signed and up to 30 days after the study drug/intervention has been discontinued. Clinically significant adverse changes in clinical status, ECGs, and physical examinations are considered AEs. Any participant complaint associated with such an abnormal finding will also be reported as an AE. AEs/SAEs will be collected for all untoward occurrences after randomization.

Adverse events include but are not limited to: (1) worsening or change in nature, severity, or frequency of conditions or symptoms present at the start of the study; (2) participant deterioration due to primary illness; (3) inter-current medical illness; and (4) drug interaction. An abnormal laboratory value will only be reported as an AE it if requires therapeutic medical intervention, if the investigator considers it to be an AE, or if it leads to the participant being withdrawn from the study.

The investigator should attempt to establish a diagnosis of the event based on signs, symptoms, and/or other clinical information. In such cases, the diagnosis should be documented as the AE and not the individual signs/symptoms. Symptoms and conditions present at the beginning of the study will be characterized, so that AEs can be defined as any new symptom, or any increase in frequency or severity of an existing symptom.

Following questioning and evaluation, all AEs, whether determined to be related or unrelated to the study drug by the site Principal Investigator, must be documented in the participant's medical records, in accordance with the investigator's normal clinical practice. Each AE is evaluated for duration, severity, seriousness, and causal relationship to the study drug. All AEs will be noted by inpatient staff in the subject's medical record. Inpatient staff will alert an unblinded clinician in the event of all AEs to determine the severity of the event. Communication between inpatient staff and the study team will occur daily in day to day interactions, phone calls, emails, and through the patient's medical record.

Serious Adverse Event (SAE). Any untoward medical occurrence that: results in death, is life-threatening, requires inpatient hospitalization or prolongation of existing hospitalization, results in persistent or significant disability/incapacity, or is a congenital anomaly/birth defect (NIH Guide-6/11/99).

Note 1: Medical and scientific judgment should be exercised in deciding whether expedited reporting is appropriate in other situations, such as important medical events that may not be immediately life-threatening or result in death or hospitalization but may jeopardize the subject or may require intervention to prevent one of the outcomes listed in the definition above.

Note 2: Hospitalizations that fulfill one of the following conditions will not have to be reported as SAE:

- Hospitalizations for social reasons (such as family or participant request) and thus unrelated to a deterioration of the subject's condition or adverse event (e.g., respite care for the caregiver).
- Hospitalizations for elective surgical interventions for which the date had already been determined prior to the study participation.

Plans for Reporting Adverse Events and Serious Adverse Events.

Study PIs (Drs. Forester and Petrides), any additional site PIs, and their research staffs will monitor the study procedures for overall safety and scientific relevance on an ongoing basis. Drs. Forester and Petrides and the site PIs (in conjunction with the DSMB as necessary) will evaluate every SAE for safety and causality and will determine whether the SAE affects the Risk/Benefit ratio of the study and whether modifications to the protocol or consent form are required.

These adverse events or unanticipated problems involving risks to subjects or others will be reported to the Mass General Brigham Human Research Committee (serving as the single IRB for this study) within 5 business days or 7 calendar days and to the DSMB within 48 hours of it becoming known to the PI or site PIs, using the appropriate forms found on the website. AEs will be reported and discussed at each regularly scheduled DSMB teleconference meetings (approximately every 3 months).

H. Risks of ancillary medications

Ancillary medications will be administered for treatment emergent effects (e.g., nausea, headache, blood pressure change) as needed according to standard ECT protocols at each institution. PRN medications to manage acute agitation include diazepam, gabapentin, midazolam, lorazepam, trazodone, risperidone, quetiapine, olanzapine, and haloperidol. These psychotropic medications are often used in regular clinical practice and common side effects include: drowsiness, dizziness, sleep problems, muscle weakness, headache, and blurred vision.

Ancillary medications will be administered as a part of routine ECT practice. Along with the necessary effects, medications may cause some unwarranted effects. Common anesthetic agents for induction include Methohexital, Etomidate, Propofol, and Remifentanil. Common side effects for anesthetic agents include drowsiness, tiredness, weakness, shivering or trembling. The paralytic agent used is Succinylcholine. Side effects for paralytic agents include rapid heartbeat, rapid breathing, dizziness, headache, chest pain, and increased body temperature. Common agents for blood pressure control include Glycopyrrolate, Atropine, Esmolol, Labetalol, and Nicardepine. Side effects of blood pressure medications include body aches, chills, constipation, cough, decrease in urination, difficulty breathing, ear congestion, warmth, fever, headache, loss of voice, nasal congestion, painful urination, facial redness, runny nose, sneezing, and sore throat. Common agents for nausea include Dexamethasone, Ondansetron, and Promethazine. Common side effects of dexamethasone include aggression, agitation, anxiety, blurred vision, decreased urine, dizziness, irregular heartbeat, headache, mood changes, numbness, shortness of breath, swelling, and weight gain. Common side effects of Ondansetron include headache, fatigue, constipation or diarrhea, dizziness, rash, hiccups, and flushing. Rare side effects of promethazine include seizures, rapid breathing, rapid heartbeat, fever, irregular blood pressure, sweating, loss of bladder control, muscle stiffness, pale skin, and weakness. Common agent for pain includes ketorolac, acetaminophen, and ibuprofen. Common side effects for ketorolac includes headache, drowsiness, indigestion, nausea, diarrhea, dizziness, itching,

swelling, constipation, and high blood pressure. Common side effects for acetaminophen include nausea, stomach pain, loss of appetite, itching, rash, and headache. Common side effects of ibuprofen include nausea, heartburn, vomiting, dizziness, headache, itching, rash, and tinnitus.

IX. POTENTIAL BENEFITS

Potential benefits of proposed research to subjects and others, and importance of knowledge to be gained

Potential benefits. This study will further our understanding of the efficacy and safety of electroconvulsive therapy to treat severe agitation in patients with probable Alzheimer's dementia. Although this treatment may help participants, there is no guarantee of any direct benefit to participants. Therefore, benefits may be indirect in that they will be helping us understand how to best treat severe agitation in dementia and the specific role of ECT. All subjects will have a thorough medical, psychiatric, and cognitive evaluation. An indirect benefit includes the opportunity to contribute to a scientific investigation that may benefit others with similar illnesses in the future. If requested by any study subject or health care agent providing consent, and following sIRB guidelines, information obtained in this study will be available to them and their primary clinician.

Reimbursement. Subjects will be reimbursed a total of $300 ($50 at randomization, $50 at the completion of the 3 week protocol, and $50 at each naturalistic follow up visit at months 1, 3, 6, and 12).

Risks vs. Benefits. The risks of study procedures (including phlebotomy and administration of clinical rating scales and cognitive assessment) are small, and the subjects will be informed of the extent of those risks in an IRB-approved consent form. The study has potential benefit with greater than minimal risk subjects who receive active ECT. The risk of ECT includes the risk of anesthesia and the potential cognitive adverse effects of ECT treatment. Further details regarding ECT risks are described above (See above Section VIII.E). The potential benefit of ECT for the treatment of agitation in dementia includes a substantial improvement in severe agitation that itself may be associated with increased morbidity and mortality for individuals with dementia. For those who receive simulated ECT, there is expected to be no direct benefit but no greater than minimal risk. In summary, the risks associated with study procedures are no more than what would be expected in routine clinical and cognitive evaluations, and routine clinical ECT practice. Thus, there is a strong benefit to risk ratio.

Importance of Knowledge to be Gained. The data to be collected may lead to significantly improved treatment of severe agitation in patients with probable Alzheimer's dementia. Current behavioral and pharmacological treatments are only modestly effective for agitation in dementia and have more limited impact in those individuals with dementia and severe agitation. Thus, there is a critical need to find more effective therapies for these patients, particularly given that severe agitation is associated with increased morbidity and mortality for individuals with dementia.

X. STUDY-LEVEL SAFETY DATA MONITORING

A. List of Abbreviations

AE	Adverse Event
CI	Confidence Interval
CRF	Case Report Form
DC	ECT-AD Data Management and Statistical Center
DSMB	Data and Safety Monitoring Board
NIA	National Institute of Aging
PI	Principal Investigator
PM	Project Manager
SAE	Serious Adverse Event
sIRB	Single Institutional Review Board
UP	Unanticipated or Unexpected Problems

B. Personnel Responsible for the Safety Monitoring

OVERVIEW

Monitoring study subject safety in the ECT-AD trial is the responsibility of the following groups:
Study site investigators
Study site study coordinators
Medical Safety Monitor
Study Data Management and Statistical Center (Data Center)
Data and Safety Monitoring Board (DSMB Members
sIRB
The Clinical Site PIs, the ECT-AD DATA CENTER, the Medical Safety Monitor, and the sIRB are responsible for the timely dissemination of the safety data to the parties responsible for safety monitoring. A detailed discussion of the monitoring process and responsibilities of each entity is presented in Section G.

C. Definitions

C.1 ADVERSE EVENT
An AE is defined as any change whether undesired, noxious, or pathological in a subject illustrated by signs or symptoms that occur during study participation whether or not it is related to study procedures.

C.2 SERIOUS ADVERSE EVENT
AEs are classified as either serious or non-serious. An SAE is any adverse event that results in any of the following outcomes or actions:

- Death due to any cause;
- A life-threatening adverse experience (i.e., the subject was at immediate risk of death from the event as it occurred);

- Inpatient hospitalization or prolongation of existing hospitalization. (Hospitalizations scheduled before enrollment for an elective procedure or treatment of a pre-existing condition that has not worsened during participation in the study is not considered a serious adverse event);
- A persistent or significant disability/incapacity (i.e., a substantial disruption of one's ability to conduct normal life functions);
- A congenital anomaly/birth defect; and,
- An important medical event that may not result in death, be life-threatening, or require hospitalization, but may jeopardize the subject and may require medical or surgical intervention to prevent one of the outcomes listed in this definition (e.g., a new diagnosis of cancer made after study enrollment is considered an important medical event)

All SAEs must be followed for the duration of the study follow-up or until resolution, whichever comes first.

An adverse event that does not meet any of the criteria for seriousness listed above should be regarded as a non-serious AE.

C.3 UNANTICIPATED OR UNEXPECTED PROBLEMS (UPS)

OHRP considers *unanticipated problems*, in general, to include any incident, experience, or outcome that meets **all** of the following criteria:

1. unexpected (in terms of nature, severity, or frequency) given (a) the research procedures that are described in the protocol-related documents, such as the IRB-approved research protocol and informed consent document; and (b) the characteristics of the subject population being studied;
2. related or possibly related to participation in the research (in this guidance document, *possibly related* means there is a reasonable possibility that the incident, experience, or outcome may have been caused by the procedures involved in the research); ***and***
3. suggests that the research places subjects or others at a greater risk of harm (including physical, psychological, economic, or social harm) than was previously known or recognized.

Unanticipated Problems must be reported to the sIRB per sIRB reporting timelines (See Section G, Table 3 below).

C.4 SEVERITY OF AN ADVERSE EVENT

The severity of each AE is recorded based on the Common Terminology Criteria for Adverse Events Version 4.0. The grading scale follows these general guidelines:

Grade 1 - Mild; asymptomatic or mild symptoms; clinical or diagnostic observations only; intervention not indicated.

Grade 2 - Moderate; minimal, local or noninvasive intervention indicated; limiting age-appropriate instrumental Activities of Daily Living.
Grade 3 - Severe or medically significant but not immediately life-threatening; hospitalization or prolongation of hospitalization indicated; disabling; limiting self-care Activities of Daily Living.
Grade 4 - Life-threatening consequences; urgent intervention indicated.
Grade 5 - Death related to AE

C.5 RELATIONSHIP OF AN ADVERSE EVENT TO THE STUDY INTERVENTION

The investigator, on the basis of his or her clinical judgment and the following definitions, determines the relationship of the AE to the protocol intervention as one of the following:

Unrelated
The temporal relationship between treatment exposure and the adverse event is unreasonable or incompatible and/or adverse event is clearly due to extraneous causes (e.g., underlying disease, environment)
Unlikely (must have 2)
May have reasonable or only tenuous temporal relationship to intervention.
1. Could readily have been produced by the subject's clinical state, or environmental or other interventions.
2. Does not follow known pattern of response to intervention.
3. Does not reappear or worsen with reintroduction of intervention.

Reasonable possibility (must have 2)
1. Has a reasonable temporal relationship to intervention.
2. Could not readily have been produced by the subject's clinical state or environmental or other interventions.
3. Follows a known pattern of response to intervention.

Definite (must have all 4)
1. Has a reasonable temporal relationship to intervention.
2. Could not readily have been produced by the subject's clinical state or have been due to environmental or other interventions.
3. Follows a known pattern of response to intervention.
4. Disappears or decreases with reduction in dose or cessation of intervention and recurs with re-exposure.

D. Anticipated Adverse Events

ECT is a routine, standard neurotherapeutic intervention that is widely used for the treatment of depression and other psychiatric disorders in younger and older adults. Our use of ECT in a population with dementia and severe agitation is unique and promising. Our highly experienced ECT staff at all five study sites follows treatment guidelines as set forth by the American Psychiatric Association[19]. The potential risks of ECT include the risks of anesthesia (including the very remote possibility of mortality), the possibility of headache (45%), nausea (25%) and general systemic complications, such as dizziness, appetite loss and muscle aches[19]. Post-ictal agitation can occur in up to 7-10% of patients receiving ECT[99]. These risks are temporary and

will be managed by the medical staff on the inpatient unit. Serious medical complications are very rare and include death, prolonged seizure, heart attack, stroke[100], trouble breathing, bone fractures, and mouth problems such as loosening of teeth or other dental fixtures or a cut lip or tongue. The most common adverse effect of ECT is neurocognitive (e.g., memory impairment), which tends to be transient. Subjects and authorized HCPs providing consent will be provided a detailed description of the risks associated with ECT. In addition to the consent form, we have prepared a subject information pamphlet outlining the benefits and risks of ECT and also documenting and explaining the clinical data collected thus far that supports the safety and efficacy of ECT for agitation in patients with dementia. These risks are not beyond what is expected for routine, clinical ECT. Right unilateral electrode (RUL) placement and ultra-brief pulse width are used in order to decrease the extent of adverse effects, particularly neurocognitive adverse effects, while maximizing therapeutic efficacy.

E. Safety Outcomes

1. Prolonged delirium: defined as presence of prolonged delirium based on positive CAM assessments with three consecutive treatment attempts.
2. Cognitive worsening: significant decline in cognition based on a $\geq$ 6 point decline from baseline in SIB-8 total score with three consecutive treatment attempts. For participants with a total SIB-8 score of $\leq$ 5 at baseline, a significant decline in cognition is based on a decline of $\geq$30 points of the Barthel Index total score compared to baseline.
3. Global worsening: global worsening of symptomatology defined as a score of 7 (marked worsening) on the ADCS-CGIC collected after treatments 3, 6 and 9.
4. Spontaneous Seizure
5. Status Epilepticus
6. Aspiration Pneumonia
7. Fractures
8. Severe or Medically Significant Cardiovascular Event (eg. Myocardial infarction)
9. Stroke
10. Death

Detailed information on safety outcomes is provided in table 2 in section F and in section G, including monitoring rules, incidence rates and clinical justifications.

F. SAFETY MONITORING RULES AND RATIONALE FOR SERIOUS ADVERSE EVENTS OF SPECIAL INTEREST

The DSMB will monitor the following specific SAEs potentially related to the disease or study interventions (adverse event of special interest, AESI) in addition to other unanticipated adverse events potentially affecting safety of study participants. Table 2 provides the boundary values for each "cluster" of patients in each intervention group that trigger an *immediate* DSMB safety

review (beyond the regularly scheduled meetings). The events are monitored by WebDCU; when a boundary for a given event is crossed, the automated system instantly sends an alert to Study PIs and Study Manager who immediately call for DSMB review. Table 2 also contains observed and reported incidences for each event type. The boundary values were developed based on clinical rationale (column 5) taking these incidences into account.

Table 2. Investigator-identified stopping guidelines

Adverse event	# To halt study and trigger a DSMB	Incidence of AE in mod-sev AD with behavioral symptoms population	Incidence of AE in ECT for depression	Clinical rationale for # to halt study and trigger DSMB review
Prolonged Delirium<br><br>Definition: positive Confusion Assessment Method (CAM) score on three consecutive ECT treatment attempts	3 events out of every 5 subjects	- 82% of patients with mod-dementia and 86% of patients with sev-dementia experienced delirium, compared to 50% of those with mild dementia (Voyer, Cole, McCusker, & Belzile, 2006)[102]<br>- 44% of patients were delirious for short periods OR during the whole week (Sandberg, Gustafson, Brannstrom, & Bucht, 1998)[103]<br>- CATIE-AD: "Confusion or mental status change" 4.9% (7/142)	PRIDE: 0.4% (1/240)	We will recruit a sample of older adults with moderate to advanced dementia. Delirium due to multiple medical factors, including UTIs, pneumonia, and dehydration, are common in this population. Our number to trigger a review is within the range of the wide-ranging incidence of delirium in AD as suggested by the literature (between 5-80%). The incidence of prolonged delirium is rare (0.4%) in a population of older adults treated with ECT for Major Depression.
Cognitive Worsening<br><br>Definition: ≥ 6-point reduction compared to baseline on the Severe Impairment	3 events out of every 5 subjects	N/A	PRIDE: 1.7% (4/240)	Cognitive worsening is defined by the daily administered SIB-8 and its change from baseline. The expected decline in cognition as measured by an instrument such as the SIB-8 is expected to be very gradual. Therefore, we would not

Battery-8 (SIB-8) on three consecutive ECT treatments attempts				expect an acute decline over the course of three weeks in the absence of a superimposed delirium. We chose the same number of subjects to trigger a DSMB review for cognitive worsening as we did for prolonged delirium due to challenges in the clinical differentiation of cognitive worsening in the setting of delirium over a short period of three weeks. In the absence of prolonged delirium, the occurrence of cognitive worsening should be unlikely.
Global Worsening<br><br>Definition: a score of 7 (marked worsening) on the Alzheimer's Disease Cooperative Study-Clinical Global Impression of Change (ADCS-CGIC)	3 events out of every 5 subjects	- CATIE-AD: placebo group had a greater change in CGI-C and a smaller change in NPI from baseline to week 12 compared to any of the experimental arms of atypical antipsychotics	N/A	Global worsening for the purposes of this study refers to the worsening of behavioral symptoms as measured by the ADCS-CGIC and does not include clinical worsening in the context of delirium or cognition.<br><br>In a sample of 10 outpatients in an unpublished 12 week RCT of a psychotropic medication vs placebo with mild to moderate AD, 30% of subjects experienced global worsening regardless of treatment group allocation. Therefore, global worsening that occurred in 3 out of every 5 study subjects after

				three weeks would be of a significant concern to trigger a DSMB review.
Spontaneous Seizure	One study subject	-10-22% of patients with AD had an unexpected seizure (usually in the later stage—as defined as 6 or more years into the course of the disease)[104]<br>- 7/44 patients (15.9%) had an unexpected seizure, and all cases had progressed to severe by the time that the seizure occurred (Romanelli, Morris, Ashkin, & Coben, 1990)[105]<br>- CATIE-AD: “Seizures” 0	PRIDE: 0.4% (1/240)	Spontaneous seizures are rare in a population of older adults with moderate to severe dementia. Therefore, in this patient population receiving ECT for agitation, we will halt the study for a DSMB review for each event of a spontaneous seizure.
Status Epilepticus	One study subject	N/A	PRIDE: 0	Status epilepticus is rare in a population of older adults with moderate to severe dementia. Therefore, we would halt the study and request a DSMB review for each case of status epilepticus.
Aspiration Pneumonia	2 events out of every 5 subjects	- More common in severe AD (Kalia, 2003)[106]<br>- 30% of patients had prior hospitalization for aspiration pneumonia at study start; 26.8% of survivors @ 6 mo had hospitalization for AP and 29.1% of those who had passed @ 6 mo had been hospitalized for AP (Bosch et al., 2012)[107]	PRIDE: 0	Aspiration pneumonia is more prevalent in patients with AD than in healthy older adults with a rate of 20-30% in severe AD. Therefore, we propose a halt to the study for every two of five study subjects with AD and agitation who develop aspiration pneumonia.
Fractures	2 events out of	- Fall common among AD, fracture uncommon—2.8% of falls resulted in	PRIDE: 0.4% (1/240)	Although falling is common in this population, fractures that

	every 10 subjects	fracture (Doorn et al., 2003)[108]<br>- 20.2% of patients had a fall, and of all of the falls only 7.3% resulted in fracture; when taking into consideration just the likelihood of fracture (8 fractures per 537 patients) the rate of fracture is 1.5% ***behavioral disturbance presence in these patients—determined by a score of 8 or more on the Behavioral Pathology in Alzheimer's Disease Rating Scale (BEHAVE-AD)*****<br>(Katz, Rupnow, Kozma, & Schneider, 2004)[109]<br>- CATIE-AD: "Falls/ fractures/ injuries" 14.8% (21/142)		result from these falls are less common. Given the 10-20% fall rate in moderate to severe dementia that may result in fractures, we will halt the study and trigger a DSMB review if there is more than one fracture out of every ten subjects.
Cardiovascular (eg. MI and arrythmia)	First event, then 2 events out of every 10 subjects	N/A	PRIDE: 1.7% (4/240)	The first cardiovascular event Grade 3+[2] will trigger a study halt and DSMB review since it is uncommon, especially if new onset in individuals with no pre-existing cardiovascular condition where this would be clinically meaningful. After the first event, 2 events out of every 10 subjects would trigger a DSMB review.
Stroke	Every study subject	- Incidence rate for stroke in patients with AD with no previous stroke history was 15.9 for every 1000 patients (approximately	PRIDE: 0	Stroke from ECT is rare, so we will halt the study and trigger a DSMB review with every study

		1.6%), however, patients with early-onset AD were more likely to experience stroke than age-matched individuals without AD but this discrepancy decreases as age increases (seems to be a consistent finding across the literature) (Cook et al., 2015)[110]<br>- Not prevalence data, but one recent article found that the *risk* of hemorrhagic stroke in individuals with AD is significantly higher than those without AD (Tolppanen et al., 2013)[111]<br>-Antipsychotic use was associated with a 1.6-fold increased risk of stroke, and higher stroke risk was observed in second-generation anti-psychotics and antipsychotics with higher binding affinity for M1 muscarinic and $\alpha 2$ adrenergic receptors (Wu, Wang, Gau, Tsai, & Cheng, 2013)[112]<br>- Swedish primary care study found that of the 28.4% of patients diagnosed with Alzheimer's from 2007-2014 who died, 8.4% had ischemic stroke in their death certificate (Subic, 2018)[113]<br>- CATIE-AD: "Cerebrovascular accident/ transient		subject who experiences a CVA.

		ischemic attack" 0.7% (1/142)		
Death	Every study subject	- According to the Center for Disease Control, there were an estimated 5 million American's living with AD in 2017 and 93,500 deaths from Alzheimer's—a mortality rate of approximately 1.9% for individuals living with Alzheimer's from the disease. (CDC, 2017)[114]<br>-According to the 2019 Alzheimer's Facts and Figures, 121,404 out of 5.5 million diagnosed with AD died in 2017 (2.2% of the AD population in 2017).<br>- approximately 1.6- to 1.7-fold increase in mortality for patients with AD treated with atypical antipsychotic compared with placebo (Hui, 2016)[115]<br>- During a 180-day observation period, 14.5% of patients on antipsychotics (haloperidol, olanzapine, quetiapine, risperidone, and valproic acid) died compared to 8.4% of non-users—although data is provided regarding death rate associated with individual antipsychotics in Table 2 of the manuscript (haloperidol being the highest), we took the average (Maust et al., 2015)[116]	PRIDE: 0.4% (1/240)	Death from ECT is rare and moreover, death is the severity of an adverse event. As such, each case will halt the study for DSMB review.

		- CATIE-AD: 2.1% (3/142)		

Note: The CATIE-AD study (Schneider et al. 2006) investigated the effectiveness of atypical antipsychotics in patients with AD experiencing psychosis, aggression, or agitation.
Note: The "Prolonging remission in depressed elderly (PRIDE) study compared, in a randomized clinical trial of patients with late-life depression, the relative efficacy, functional outcomes, and tolerability of combination pharmacotherapy with venlafaxine and lithium vs the same combination of pharmacotherapy plus symptom-titrated ECT.

G. Safety Reporting Components, Requirements and Process

G.1 OVERVIEW OF REPORTING PROCESS

When sites learn of the occurrence of an AE, SAE or unanticipated problems (UPs), a report is submitted to the WebDCU™ database, which alerts the data manager to check the submitted data for completeness. Once the CRF is considered complete, the system automatically alerts the Medical Safety Monitor (MSM) to assess and verify the submitted information. PIs Forester and Petrides will act as the MSMs during the study. Concurrently the WebDCU™ automatically checks whether the boundary of a stopping rule as detailed in section F, table 2 has been crossed with this submitted SAE. If the boundary is crossed, an immediate review by the DSMB is triggered: the system alerts the project manager and study PIs to the crossing of the stopping rule boundary, who in turn alert the DSMB for the need of an immediate review (Figure 1).

Figure 1. SAE reporting process triggering _immediate_ DSMB review

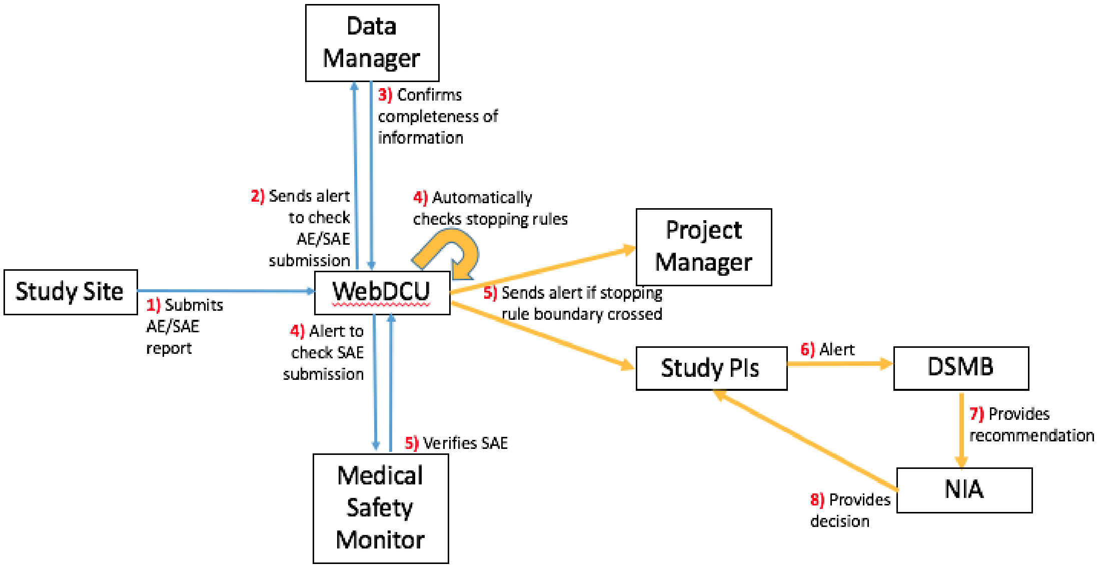

G.2 SITE RESPONSIBILITIES

All study treatment related adverse events (AEs), all serious AEs (SAEs), and all safety outcomes will be reported from randomization through Post-treatment 9 assessment or Discharge, whichever comes first. After Post-treatment 9 /Discharge, all SAEs and all safety outcomes will be reported until completion of the follow-up phase or Discharge, whichever comes first. Sites will report non-serious events in WebDCU within 5 days of the sites' awareness of the event. SAEs must be reported within 24 hours.

For each reportable AE, an AE form will be submitted capturing the details of the event including date of onset, severity, duration, and relationship to the treatment. For serious adverse events, additional information, including narrative summaries will be submitted. The site PIs are responsible for ensuring reportable AEs are submitted via WebDCU within the required timelines and updated with new information as it becomes available (e.g., date of resolution, action taken). Upon completion of the study protocol by the subject, premature withdrawal from the study by the subject, or the subject's death, all information regarding each AE must be completed, if not done earlier. In the event of a subject death during study, that should be immediately reported and all possible efforts should be made by the site to obtain relevant records from the hospital or the subject's primary care provider to determine the cause of death.

In addition to submission of AEs and Unanticipated Problems to the WebDCU™, all events must be reported to the sIRB in a timely fashion as outlined below (Table 3).

Table 3: Timeline for Investigator reporting of UP to sIRB

	sIRB reporting (from site PI awareness)	Site reporting in WebDCU (calendar days)
Unanticipated problem that is also an SAE and places subjects or others at a greater risk of harm	5 business days or 7 calendar days	24 hours from when the site becomes aware of the event
Unanticipated problem that is not an SAE but places subjects or others at a greater risk of harm	5 business days or 7 calendar days	5 days
SAEs (not UP)	At Continuing Review	24 hours
Non-serious AEs	At Continuing Review	5 days

G.3 MONITORING OF SERIOUS AEs BY THE MEDICAL SAFETY MONITOR (MSM)

The trial will have a designated Medical Safety Monitor (MSM) who will be responsible for monitoring safety data. This designated physician will have expertise in Alzheimer's dementia, ECT, clinical trials participation and clinical trial safety monitoring.

The MSM will be responsible for ongoing monitoring of reports of SAEs submitted in real time by the clinical centers to ensure good clinical practice and to identify safety concerns quickly. The MSM may suggest protocol modifications to prevent the occurrence of particular AEs or to improve expeditious identification of SAEs. To minimize bias, he/she will evaluate SAEs blinded to treatment assignment. In the event of unexpected SAEs or an unduly high rate of SAEs, the MSM will promptly notify the DSMB Chair.

The MSM will be automatically notified by the WebDCU™ triggered when adverse or serious adverse events (AEs or SAEs) are entered. The MSM will adjudicate the event for seriousness, relationship to the study intervention, and expectedness.

G.4 DSMB RESPONSIBILITIES

An NIA-approved Data and Safety Monitoring Board (DSMB) is responsible for monitoring safety data, data quality and subject recruitment. The DSMB will be constituted of three experts: two psychiatrists with expertise in clinical treatment of individuals with ECT and clinical trials involving administration of ECT to psychiatric populations, and a biostatistician experienced with clinical trial design and management. The DSMB will meet by teleconference prior to the first participant randomization, every 3 months thereafter, and as necessary along with the NIA program officer to discuss collected data, recruitment, accuracy of data collection, and frequency and nature of adverse events. The DSMB's role is to serve in a consultative capacity to inform the NIA regarding conduct of the trial. At any time, the DSMB may recommend to NIA to terminate the study if there is compelling evidence from it or other studies regarding adverse effects of study treatment that is sufficient to override any potential benefit of the study treatment to the target population.

The primary responsibility of the DSMB is to determine safety of ECT in the treatment of severe aggression in patients with Alzheimer's disease with particular focus on monitoring safety outcomes as described in section X.G.6.

The DSMB will review information supplied by the ECT-AD statistician and will make recommendations about whether to alter study procedures based on safety considerations. The PIs (Forester and Petrides) of ECT-AD will have the final responsibility about implementing such changes and will do so in accordance with IRB procedures, NIH policy, and full attention to the need to protect the safety and rights of research participants.

The DSMB will provide an initial review of the final protocol and consent. They will provide ongoing review of safety data, subject accrual, and outcome data. The DSMB will review and approve changes to the protocol and consent forms and provide routine oversight of both safety issues and data flow. They will receive serious adverse event (SAE) reports in real time and will review non-serious adverse events every three months. Other safety data as requested by the DSMB during the meeting will be reported in summary tables to the DSMB within 3 days of each DSMB meeting. At their request, safety data will be reported in partially blinded (A, B) or unblinded fashion.

A written report will be provided by the DSMB to the PIs and to NIA program staff. At a minimum, this will include a recommendation to continue the protocol as planned. Recommended changes or even discontinuation of the study may be considered, as well. The final decision to amend or terminate the study is made by the National Institute of Aging, based on the DSMB recommendation.

Additionally, the DSMB will provide medical monitoring of the study for protocol questions, violations and serious or problematic adverse events that site investigators report to the DSMB. The DSMB will be notified using a special SAE report form within 48 working hours by the investigative site via telephone, fax, or email if any of the following events occur: (1) death, (2) hospitalization for medical reasons, (3) life-threatening events or (4) any unexpected event. The DSMB will review SAE data and accompanying documents, and query sites for clarification or additional information. Should study participants be started on a treatment that requires laboratory monitoring or more medical monitoring than what is specified in the protocol, this will be conducted as part of usual care at the discretion of the site PIs. The DSMB will also have access to partially blinded data on treatment condition and adverse events four times annually, will review these data for evidence of adverse events associated with study treatment, and make a report to Drs. Forester and Petrides and the site PIs.

DSMB Meetings: The DSMB will hold regularly scheduled meetings every three months. In addition to these regular quarterly meetings, the DSMB will meet as needed if an *immediate* DSMB safety review is triggered, to review safety-related concerns if alerted by study PIs or the project manager.
The study PIs (Dr. Forester and Dr. Petrides) will be responsible for scheduling the meetings (in-person or by teleconference) and ensuring that all materials needed for review are provided with sufficient time prior to the meeting. Each meeting will be divided into two parts:

1. First, an open session will be held in which the study PIs and site PIs may be present, at the request of the DSMB, to review the conduct of the trial and to answer questions from members of the DSMB. The focus in the open session may be on subject accrual, protocol compliance, and general toxicity issues.
2. Following this session, a closed session involving only DSMB members will be held to allow the DSMB the opportunity to discuss the general conduct of the trial and all outcome results, including all safety data, adverse events and serious adverse events, develop recommendations, and take votes as necessary.

*DSMB Reports***:** To prepare for the regular quarterly meetings, the DSMB will receive two study reports, an open and a closed report: These reports focus on enrollment, data quality, safety and protocol violations and will include information on baseline characteristics and details of study status

1. Open report: The Open Report will show all trial data in aggregate.
2. Closed report: The Closed Report will show the same data as the Open Report but presented by treatment group code (A/B). It will also include investigator-identified stopping guidelines for the safety outcomes as defined in section X.E.

G.5 DATA CENTER SAFETY MONITORING RESPONSIBILITIES

The Data Center will supply a statistical safety monitoring report as requested by the DSMB for both, the regularly scheduled quarterly meetings and for the immediate reviews triggered by an AESI alert. This report includes likelihood-based statistical monitoring guidelines and intervention comparisons of safety outcomes based on information provided in Table 2 in section X.F above if requested by the DSMB. The information is used by the DSMB in conjunction with other relevant clinical data and careful judgment in deciding whether the risk to patients outweighs the benefit of treatment and, hence, whether to recommend pausing and amending or stopping the study prior to the planned total sample size. The final decision to amend or terminate the study is made by the National Institute of Aging, based on the DSMB recommendation.

G.6 SINGLE IRB (sIRB) ROLE

The sIRB will review events according to OHRP 2007 guidance. In particular, this guidance clarifies the small subset of adverse events occurring in human subjects participating in research that are unanticipated problems that must be reported under 45 CFR part 46. The guidance is intended to help ensure that the review and reporting of unanticipated problems and adverse events occur in a timely, meaningful way so that human subjects can be better protected from avoidable harms while reducing unnecessary burden.

G.7 FDA REPORTING

All AEs and SAEs will be reported to the FDA in accordance with guidelines in 21CFR812. The PIs (Forester and Petrides) will report an unanticipated adverse device effect to the FDA as soon as possible and **no later than 10 business days** after the PI first learns of the event.
Progress Reports and Annual Reports will be submitted to the sIRB and FDA on a semi-annual basis and will include (but is not limited to):
Names of investigators
Number of patients treated with the device

- Description of aims to evaluate safety and efficacy
- Summary of anticipated and unanticipated AEs and SAEs
- Description of protocol deviations (since the last progress report)

Final Report will be submitted to the cIRB and FDA within 6 months of study completion or termination and will include (but is not limited to):

- Names of investigators
- Number of subjects enrolled
- Summary of results
- Summary of anticipated and unanticipated adverse events
- Description of protocol deviations
- Risk analysis

XI. REFERENCES

1. Alzheimer’s Association, ed. 2020 Alzheimer’s Disease Facts and Figures. 2020;16(3):361+.
2. Lyketsos CG, Lopez O, Jones B, Fitzpatrick AL, Breitner J, DeKosky S. Prevalence of neuropsychiatric symptoms in dementia and mild cognitive impairment: results from the cardiovascular health study. *JAMA*. 2002;288(12):1475–1483.
3. Steinberg M, Sheppard JM, Tschanz JT. The incidence of mental and behavioral disturbances in dementia: the Cache County Study. *J Neuropsychiatry Clin Neurosci*. 2003;15(3):340–345.
4. van der Linde RM, Dening T, Stephan BCM, Prina AM, Evans E, Brayne C. Longitudinal course of behavioural and psychological symptoms of dementia: systematic review. *Br J Psychiatry J Ment Sci*. 2016;209(5):366-377. doi:10.1192/bjp.bp.114.148403
5. Livingston G. A systematic review of the clinical effectiveness and cost-effectiveness of sensory, psychological and behavioural interventions for managing agitation in older adults with dementia. *Health Technol Assess*. 2014;18(39):1–226, .
6. Cohen-Mansfield J. Agitated behaviors in the elderly. II. Preliminary results in the cognitively deteriorated. *J Am Geriatr Soc*. 1986;34(10):722–727.
7. Bartels SJ, Horn SD, Smout RJ, et al. Agitation and depression in frail nursing home elderly patients with dementia: treatment characteristics and service use. *Am J Geriatr Psychiatry*. 2003;11(2):231–238.
8. Steinberg M, Shao H, Zandi P, et al. Point and 5-year period prevalence of neuropsychiatric symptoms in dementia: the Cache County Study. *Int J Geriatr Psychiatry*. 2008;23(2):170-177. doi:10.1002/gps.1858
9. Ferrara M, Langiano E, Di Brango T, De Vito E, Di Cioccio L, Bauco C. Prevalence of stress, anxiety and depression in with Alzheimer caregivers. *Health Qual Life Outcomes*. 2008;6:93. doi:10.1186/1477-7525-6-93
10. Lopez OL, Becker JT, Chang Y-F, et al. The long-term effects of conventional and atypical antipsychotics in patients with probable Alzheimer’s disease. *Am J Psychiatry*. 2013;170(9):1051-1058. doi:10.1176/appi.ajp.2013.12081046
11. Hirono N, Mega MS, Dinov ID, Mishkin F, Cummings JL. Left frontotemporal hypoperfusion is associated with aggression in patients with dementia. *Arch Neurol*. 2000;57(6):861–866.
12. Kales HC, Gitlin LN, Lyketsos CG, Lyketsos CG. Management of neuropsychiatric symptoms of dementia in clinical settings: recommendations from a multidisciplinary expert panel. *J Am Geriatr Soc*. 2014;62(4):762–769.
13. Sink KM, Holden KF, Yaffe K. Pharmacological treatment of neuropsychiatric symptoms of dementia: a review of the evidence. *JAMA*. 2005;293(5):596–608.
14. Ballard C, Waite J. The effectiveness of atypical antipsychotics for the treatment of aggression and psychosis in Alzheimer’s disease. *Cochrane Database Syst Rev*. Published online 2006.
15. Schneider LS, Dagerman KS, Insel P. Risk of death with atypical antipsychotic drug treatment for dementia: meta-analysis of randomized placebo-controlled trials. *JAMA*. 2005;294(15):1934–1943.
16. Kales HC, Kim HM, Zivin K, et al. Risk of mortality among individual antipsychotics in patients with dementia. *Am J Psychiatry*. 2012;169(1):71-79. doi:10.1176/appi.ajp.2011.11030347
17. Porsteinsson AP, Drye LT, Pollock BG, et al. Effect of citalopram on agitation in Alzheimer disease: the CitAD randomized clinical trial. *JAMA*. 2014;311(7):682-691. doi:10.1001/jama.2014.93
18. Ely E. *The Remembrance Project: Louise Finocchio*.; 2016.
19. American Psychiatric Association. *Practice of Electroconvulsive Therapy: Recommendations for Treatment, Training, and Privileging: A Task Force Report of the American Psychiatric Association*. American Psychiatric Association [APA]. Committee on

Electroconvulsive Therapy; 2001. Accessed July 8, 2020. https://repository.library.georgetown.edu/handle/10822/941372
20. Wahlund B, von Rosen D. ECT of major depressed patients in relation to biological and clinical variables: a brief overview. *Neuropsychopharmacol Off Publ Am Coll Neuropsychopharmacol*. 2003;28 Suppl 1:S21-26. doi:10.1038/sj.npp.1300135
21. Guloksuz S, Rutten BP, Arts B, Os J, Kenis G. The immune system and electroconvulsive therapy for depression. *J ECT*. 2014;30(2):132–137.
22. Kranaster L, Aksay SS, Bumb JM, et al. Electroconvulsive therapy selectively enhances amyloid β 1-42 in the cerebrospinal fluid of patients with major depression: A prospective pilot study. *Eur Neuropsychopharmacol J Eur Coll Neuropsychopharmacol*. 2016;26(12):1877-1884. doi:10.1016/j.euroneuro.2016.11.004
23. Takeda S, Sato N, Morishita R. Systemic inflammation, blood-brain barrier vulnerability and cognitive/non-cognitive symptoms in Alzheimer disease: relevance to pathogenesis and therapy. *Front Aging Neurosci*. 2014;6:171.
24. Philibert RA, Richards L, Lynch CF, Winokur G. Effect of ECT on mortality and clinical outcome in geriatric unipolar depression. *J Clin Psychiatry*. 1995;56(9):390–394.
25. Kerner N, Prudic J. Current electroconvulsive therapy practice and research in the geriatric population. *Neuropsychiatry*. 2014;4(1):33-54. doi:10.2217/npy.14.3
26. Rasmussen K. The Practice of Electroconvulsive Therapy: Recommendations for Treatment, Training, and Privileging (Second Edition). *J ECT*. 2002;18(1). https://journals.lww.com/ectjournal/Fulltext/2002/03000/The_Practice_of_Electroconvulsive_Therapy_.15.aspx
27. Tomac TA, Rummans TA, Pileggi TS, Li H. Safety and efficacy of electroconvulsive therapy in patients over age 85. *Am J Geriatr Psychiatry*. 1997;5(2):126–130.
28. Tew JD, Mulsant BH, Haskett RF, et al. Acute efficacy of ECT in the treatment of major depression in the old-old. *Am J Psychiatry*. 1999;156(12):1865-1870. doi:10.1176/ajp.156.12.1865
29. O'Connor MK, Knapp R, Husain M, et al. The influence of age on the response of major depression to electroconvulsive therapy: a C.O.R.E. Report. *Am J Geriatr Psychiatry*. 2001;9(4):382–390.
30. Dombrovski AY, Mulsant BH, Haskett RF, Prudic J, Beagly AE, Sackeim HA. Predictors of remission after electroconvulsive therapy in unipolar major depression. *J Clin Psychiatry*. 2005;66(8):1043–1049.
31. Kellner CH, McClintock SM, McCall WV, et al. Brief pulse and ultrabrief pulse right unilateral electroconvulsive therapy (ECT) for major depression: efficacy, effectiveness, and cognitive effects. *J Clin Psychiatry*. 2014;75(7):777. doi:10.4088/JCP.14lr08997
32. Kellner CH, Husain MM, Knapp RG, et al. Right Unilateral Ultrabrief Pulse ECT in Geriatric Depression: Phase 1 of the PRIDE Study. *Am J Psychiatry*. 2016;173(11):1101-1109. doi:10.1176/appi.ajp.2016.15081101
33. Kellner CH, Husain MM, Knapp RG, et al. A Novel Strategy for Continuation ECT in Geriatric Depression: Phase 2 of the PRIDE Study. *Am J Psychiatry*. 2016;173(11):1110–1118.
34. Ujkaj M, Davidoff DA, Seiner SJ, Ellison JM, Harper DG, Forester BP. Safety and efficacy of electroconvulsive therapy for the treatment of agitation and aggression in patients with dementia. *Am J Geriatr Psychiatry*. 2012;20(1):61–72.
35. Acharya D, Harper DG, Achtyes ED, et al. Safety and utility of acute electroconvulsive therapy for agitation and aggression in dementia. *Int J Geriatr Psychiatry*. 2015;30(3):265-273. doi:10.1002/gps.4137
36. Deshmukh DK, Sethna KJ, Nagesh RP, Haridas RM, Patankar SV. A comparative study to evaluate the efficacy of spaced v/s daily electroconvulsive therapy in schizophrenia. *Indian J Psychiatry*. 1980;22(4):366-367.

37. Holmberg SK, Tariot PN, Challapalli R. Efficacy of ECT for agitation in dementia: a case report. *Am J Geriatr Psychiatry*. 1996;4(4):330–334.
38. Bang J, Price D, Prentice G, Campbell J. ECT treatment for two cases of dementia-related pathological yelling. *J Neuropsychiatry Clin Neurosci*. 2008;20(3):379–380.
39. Wu Q, Prentice G, Campbell JJ. ECT treatment for two cases of dementia-related aggressive behavior. *J Neuropsychiatry Clin Neurosci*. 2010;22(2):10– 11.
40. Aksay SS, Hausner L, Frolich L, Lartorius A. Severe agitation in severe early-onset Alzheimer's disease resolves with ECT. *Neuropsychiatr Dis Treat*. 2014;10:2147–2151.
41. Goswami U, Kumar U, Singh B. Efficacy of electroconvulsive therapy in treatment resistant schizophrenia: a double-blind study. *Indian J Psychiatry*. 2003;45(1):26–29.
42. Sutor B, Rasmussen KG. Electroconvulsive therapy for agitation in Alzheimer disease: a case series. *J ECT*. 2008;24(3):239–241.
43. Grant JE, Mohan SN. Treatment of agitation and aggression in four demented patients using ECT. *J ECT*. 2001;17(3):205-209. doi:10.1097/00124509-200109000-00012
44. Zhang Q-E, Sha S, Ungvari GS, et al. Demographic and Clinical Profile of Patients With Dementia Receiving Electroconvulsive Therapy: A Case-Control Study. *J ECT*. 2016;32(3):183-186. doi:10.1097/YCT.0000000000000314
45. Tang Y. ECT for the treatment of agitation in dementia. Poster Presentation presented at the: American Association of Geriatric Psychiatry Annual Meeting; March 2014; Orlando, FL.
46. Glass OM, Forester BP, Hermida AP. Electroconvulsive therapy (ECT) for treating agitation in dementia (major neurocognitive disorder) - a promising option. *Int Psychogeriatr*. 2017;29(5):717-726. doi:10.1017/S1041610216002258
47. van den Berg JF, Kruithof HC, Kok RM, Verwijk E, Spaans H-P. Electroconvulsive Therapy for Agitation and Aggression in Dementia: A Systematic Review. *Am J Geriatr Psychiatry Off J Am Assoc Geriatr Psychiatry*. 2018;26(4):419-434. doi:10.1016/j.jagp.2017.09.023
48. Vasavada MM, Leaver AM, Njau S, et al. Short- and Long-term Cognitive Outcomes in Patients With Major Depression Treated With Electroconvulsive Therapy. *J ECT*. 2017;33(4):278-285. doi:10.1097/YCT.0000000000000426
49. *Diagnostic and Statistical Manual of Mental Disorders (DSM 5)*. 5th ed. A.P. Publishing; 2013.
50. Hermida AP, Tang Y-L, Glass O, Janjua AU, McDonald WM. Efficacy and Safety of ECT for Behavioral and Psychological Symptoms of Dementia (BPSD): A Retrospective Chart Review. *Am J Geriatr Psychiatry Off J Am Assoc Geriatr Psychiatry*. 2020;28(2):157-163. doi:10.1016/j.jagp.2019.09.008
51. Schneider LS, Olin JT, Doody RS, et al. Validity and reliability of the Alzheimer“s Disease Cooperative Study-Clinical Global Impression of Change. The Alzheimer”s Disease Cooperative Study. *Alzheimer Dis Assoc Disord*. 1997;11 Suppl 2:22– 32.
52. Schneider LS, Olin JT. Clinical global impressions in Alzheimer's clinical trials. *Int Psychogeriatr*. 1996;8(2):277–88 288–90.
53. de Medeiros K, Robert P, Gauthier S, et al. The Neuropsychiatric Inventory-Clinician rating scale (NPI-C): reliability and validity of a revised assessment of neuropsychiatric symptoms in dementia. *Int Psychogeriatr*. 2010;22(6):984-994. doi:10.1017/S1041610210000876
54. Rosen J, Burgio L, Kollar M, et al. A user-friendly instrument for rating agitation in dementia patients. *Am J Geriatr Psychiatry*. 1994;2(1):52–59.
55. McKhann GM, Knopman DS, Chertkow H, et al. The diagnosis of dementia due to Alzheimer's disease: recommendations from the National Institute on Aging-Alzheimer's Association workgroups on diagnostic guidelines for Alzheimer's disease. *Alzheimers Dement*. 2011;7(3):263–269.

56. Alzheimer's Association. Research consent for cognitively impaired adults: recommendations for institutional review boards and investigators. *Alzheimer Dis Assoc Disord*. 2004;18(3):171-175. doi:10.1097/01.wad.0000137520.23370.56
57. Zhao W, Hill MD, Palesch Y. Minimal sufficient balance-a new strategy to balance baseline covariates and preserve randomness of treatment allocation. *Stat Methods Med Res*. 2015;24(6):989-1002. doi:10.1177/0962280212436447
58. Sundsted KK, Burton MC, Shah R, Lapid MI. Preanesthesia medical evaluation for electroconvulsive therapy: a review of the literature. *J ECT*. 2014;30(1):35-42. doi:10.1097/YCT.0b013e3182a3546f
59. Kellner CH, Knapp RG, Petrides G, et al. Continuation electroconvulsive therapy vs pharmacotherapy for relapse prevention in major depression: a multisite study from the Consortium for Research in Electroconvulsive Therapy (CORE. *Arch Gen Psychiatry*. 2006;63(12):1337–1344.
60. Sackeim HA, Prudic J, Devanand DP, et al. Effects of stimulus intensity and electrode placement on the efficacy and cognitive effects of electroconvulsive therapy. *N Engl J Med*. 1993;328(12):839-846. doi:10.1056/NEJM199303253281204
61. Sackeim HA, Prudic J, Fuller R, Keilp J, Lavori PW, Olfson M. The cognitive effects of electroconvulsive therapy in community settings. *Neuropsychopharmacology*. 2007;32(1):244–254.
62. Kellner CH, Knapp R, Husain MM, et al. Bifrontal, bitemporal and right unilateral electrode placement in ECT: randomised trial. *Br J Psychiatry J Ment Sci*. 2010;196(3):226-234. doi:10.1192/bjp.bp.109.066183
63. McClintock SM, Choi J, Deng Z-D, Appelbaum LG, Krystal AD, Lisanby SH. Multifactorial determinants of the neurocognitive effects of electroconvulsive therapy. *J ECT*. 2014;30(2):165-176. doi:10.1097/YCT.0000000000000137
64. Dixon JS, Saddington DG, Shiles CJ, Sreevalsan KP, Munro CA, Rosenberg PB. Clinical evaluation of brief cognitive assessment measures for patients with severe dementia. *Int Psychogeriatr*. 2017;29(7):1169-1174. doi:10.1017/S1041610217000151
65. Schmitt FA, Saxton J, Ferris SH, Mackell J, Sun Y. Evaluation of an 8-item Severe Impairment Battery (SIB-8) vs. the full SIB in moderate to severe Alzheimer's disease patients participating in a donepezil study. *Int J Clin Pract*. 2013;67(10):1050-1056. doi:10.1111/ijcp.12188
66. Inouye SK, Dyck CH, Alessi CA, Balkin S, Siegal AP, Horwitz RI. Clarifying confusion: the confusion assessment method. A new method for detection of delirium. *Ann Intern Med*. 1990;113(12):941–948.
67. Kiely DK, Jones RN, Bergmann MA, Marcantonio ER. Association between psychomotor activity delirium subtypes and mortality among newly admitted post-acute facility patients. *J Gerontol A Biol Sci Med Sci*. 2007;62(2):174-179. doi:10.1093/gerona/62.2.174
68. Duff K. Evidence-based indicators of neuropsychological change in the individual patient: relevant concepts and methods. *Arch Clin Neuropsychol Off J Natl Acad Neuropsychol*. 2012;27(3):248-261. doi:10.1093/arclin/acr120
69. Kamiya M, Sakurai T, Ogama N, Maki Y, Toba K. Factors associated with increased caregivers' burden in several cognitive stages of Alzheimer's disease. *Geriatr Gerontol Int*. 2014;14 Suppl 2:45-55. doi:10.1111/ggi.12260
70. Bradshaw J, Saling M, Hopwood M, Anderson V, Brodtmann A. Fluctuating cognition in dementia with Lewy bodies and Alzheimer's disease is qualitatively distinct. *J Neurol Neurosurg Psychiatry*. 2004;75(3):382-387. doi:10.1136/jnnp.2002.002576
71. Mahoney FI, Barthel DW. FUNCTIONAL EVALUATION: THE BARTHEL INDEX. *Md State Med J*. 1965;14:61-65.

72. Liu-Seifert H, Siemers E, Selzler K, et al. Correlation between Cognition and Function across the Spectrum of Alzheimer's Disease. *J Prev Alzheimers Dis*. 2016;3(3):138-144. doi:10.14283/jpad.2016.99
73. Cohen-Mansfield J. Conceptualization of agitation: results based on the Cohen-Mansfield Agitation Inventory and the Agitation Behavior Mapping Instrument. *Int Psychogeriatr*. 1996;8 Suppl 3:309-315; discussion 351-354. doi:10.1017/s1041610297003530
74. Cohen-Mansfield J, Billig N. Agitated behaviors in the elderly. I. A conceptual review. *J Am Geriatr Soc*. 1986;34(10):711–721.
75. Koss E, Weiner M, Ernesto C, et al. Assessing patterns of agitation in Alzheimer's disease patients with the Cohen-Mansfield Agitation Inventory. The Alzheimer's Disease Cooperative Study. *Alzheimer Dis Assoc Disord*. 1997;11 Suppl 2:S45-50. doi:10.1097/00002093-199700112-00007
76. Whall AL, Black MEA, Yankou DJ, et al. Nurse aides' identification of onset and level of agitation in late stage dementia patients. *Am J Alzheimer's Dis*. 1999;14(4):202–206.
77. Zarit SH, Reever KE, Bach-Peterson J. Relatives of the impaired elderly: correlates of feelings of burden. *Gerontologist*. 1980;20(6):649–55.
78. Weiner MF, Martin-Cook K, Svetlik DA, Saine K, Foster B, Fontaine CS. The quality of life in late-stage dementia (QUALID) scale. *J Am Med Dir Assoc*. 2000;1(3):114-116.
79. Cummings JL, Mega M, Gray K, Rosenberg-Thompson S, Carusi DA, Gornbein J. The Neuropsychiatric Inventory: comprehensive assessment of psychopathology in dementia. *Neurology*. 1994;44(12):2308–2314.
80. Alexopoulos GS, Abrams RC, Young RC, Shamoian CA. Cornell Scale for Depression in Dementia. *Biol Psychiatry*. 1988;23(3):271-284. doi:10.1016/0006-3223(88)90038-8
81. van den Elsen GAH, Ahmed AIA, Verkes R-J, et al. Tetrahydrocannabinol for neuropsychiatric symptoms in dementia. *Neurology*. 2015;84(23):2338-2346. doi:10.1212/WNL.0000000000001675
82. Panisset M, Roudier M, Saxton J, Boller F. Severe impairment battery. A neuropsychological test for severely demented patients. *Arch Neurol*. 1994;51(1):41–45.
83. Folstein MF, Folstein SE, McHugh PR. "Mini-mental state". A practical method for grading the cognitive state of patients for the clinician. *J Psychiatr Res*. 1975;12(3):189-198. doi:10.1016/0022-3956(75)90026-6
84. Sienaert P, Rooseleer J, Fruyt JD. Measuring catatonia: a systematic review of rating scales. *J Affect Disord*. 2011;135(1-3):1–9.
85. Inouye SK, Kosar CM, Tommet D, et al. The CAM-S: development and validation of a new scoring system for delirium severity in 2 cohorts. *Ann Intern Med*. 2014;160(8):526–533.
86. Lee HB, Mears SC, Rosenberg PB, Leoutsakos J-MS, Gottschalk A, Sieber FE. Predisposing factors for postoperative delirium after hip fracture repair in individuals with and without dementia. *J Am Geriatr Soc*. 2011;59(12):2306–2313.
87. Fong TG, Tulebaev SR, Inouye SK. Delirium in elderly adults: diagnosis, prevention and treatment. *Nat Rev Neurol*. 2009;5(4):210–20.
88. Wei LA, Fearing MA, Sternberg EJ, Inouye SK. The confusion assessment method: a systematic review of current usage. *JAGS*. 2008;56:823–830.
89. Steis MR, Evans L, Hirschman KB, et al. Screening for delirium using family caregivers: convergent validity of the Family Confusion Assessment Method and interviewer-rated Confusion Assessment Method. *J Am Geriatr Soc*. 2012;60(11):2121-2126. doi:10.1111/j.1532-5415.2012.04200.x
90. Fitzmaurice GM, Laird NM, Ware JH. *Applied Longitudinal Analysis*. 2nd ed.; 2011.
91. O'Kelly M, Ratitch B. *Clinical Trials With Missing Data: A Guide for Practitioners*. John Wiley & Sons; 2014.
92. Hedeker D, Gibbons RD. *Longitud Data Anal*. Published online 2006:360.
93. Little R, Rubin D. *Statistical Analysis with Missing Data*. John Wiley and Sons, Inc; 1987.

94. McCulloch CE, Searle SR. *Generalized, Linear, and Mixed Models*. 1st Edition. John Wiley & Sons, Inc.; 2001.
95. Petrides G, Fink M. The “half-age” stimulation strategy for ECT dosing. *Convuls Ther*. 1996;12(3):138–46.
96. Worthington H. Methods for pooling results from multi-center studies. *J Dent Res*. Published online 2004:119–21.
97. Fleiss JL. Analysis of data from multiclinic trials. *Control Clin Trials*. 1986;7(4):267–75.
98. ICH Harmonised Tripartite Guideline. Statistical principles for clinical trials. *Int Conf Harmon E9 Expert Work Group Stat Med*. 1999;18(15):1905–42.
99. MedDRA Best Practices: Maintenance and Support Services Organization (MSSO) Reccomendations for Implementation and Use of MedDRA. Published online 2018. https://admin.new.meddra.org/sites/default/files/guidance/file/000026_meddra_best_practices_2018_0.pdf
100. Augoustides JG, Greenblatt E, Abbas MA, O’Reardon JP, Datto CJ. Clinical approach to agitation after electroconvulsive therapy: a case report and literature review. *J ECT*. 2002;18(4):213-217. doi:10.1097/00124509-200212000-00009
101. Zielinski RJ, Roose SP, Devanand DP, Woodring S, Sackeim HA. Cardiovascular complications of ECT in depressed patients with cardiac disease. *Am J Psychiatry*. 1993;150(6):904-909. doi:10.1176/ajp.150.6.904
102. Voyer P, Cole MG, McCusker J, Belzile E. Prevalence and symptoms of delirium superimposed on dementia. *Clin Nurs Res*. 2006;15(1):46-66. doi:10.1177/1054773805282299
103. Sandberg O, Gustafson Y, Brännström B, Bucht G. Prevalence of dementia, delirium and psychiatric symptoms in various care settings for the elderly. *Scand J Soc Med*. 1998;26(1):56-62. doi:10.1177/14034948980260011201
104. Mendez M, Lim G. Seizures in elderly patients with dementia: epidemiology and management. *Drugs Aging*. 2003;20(11):791-803. doi:10.2165/00002512-200320110-00001
105. Romanelli MF, Morris JC, Ashkin K, Coben LA. Advanced Alzheimer’s disease is a risk factor for late-onset seizures. *Arch Neurol*. 1990;47(8):847-850. doi:10.1001/archneur.1990.00530080029006
106. Kalia M. Dysphagia and aspiration pneumonia in patients with Alzheimer’s disease. *Metabolism*. 2003;52(10 Suppl 2):36-38. doi:10.1016/s0026-0495(03)00300-7
107. Bosch X, Formiga F, Cuerpo S, Torres B, Rosón B, López-Soto A. Aspiration pneumonia in old patients with dementia. Prognostic factors of mortality. *Eur J Intern Med*. 2012;23(8):720-726. doi:10.1016/j.ejim.2012.08.006
108. van Doorn C, Gruber-Baldini AL, Zimmerman S, et al. Dementia as a risk factor for falls and fall injuries among nursing home residents. *J Am Geriatr Soc*. 2003;51(9):1213-1218. doi:10.1046/j.1532-5415.2003.51404.x
109. Katz IR, Rupnow M, Kozma C, Schneider L. Risperidone and falls in ambulatory nursing home residents with dementia and psychosis or agitation: secondary analysis of a double-blind, placebo-controlled trial. *Am J Geriatr Psychiatry Off J Am Assoc Geriatr Psychiatry*. 2004;12(5):499-508. doi:10.1176/appi.ajgp.12.5.499
110. Cook M, Baker N, Lanes S, Bullock R, Wentworth C, Arrighi HM. Incidence of stroke and seizure in Alzheimer’s disease dementia. *Age Ageing*. 2015;44(4):695-699. doi:10.1093/ageing/afv061
111. Tolppanen A-M, Lavikainen P, Solomon A, Kivipelto M, Soininen H, Hartikainen S. Incidence of stroke in people with Alzheimer disease: a national register-based approach. *Neurology*. 2013;80(4):353-358. doi:10.1212/WNL.0b013e31827f08c5

112. Wu C-S, Wang S-C, Gau SS-F, Tsai H-J, Cheng Y-C. Association of stroke with the receptor-binding profiles of antipsychotics-a case-crossover study. *Biol Psychiatry*. 2013;73(5):414-421. doi:10.1016/j.biopsych.2012.07.006
113. Subic A, Zupanic E, von Euler M, et al. Stroke as a Cause of Death in Death Certificates of Patients with Dementia: A Cohort Study from the Swedish Dementia Registry. *Curr Alzheimer Res*. 2018;15(14):1322-1330. doi:10.2174/1567205015666181002134155
114. CDC. Deaths from Alzheimer's Disease. Centers for Disease Control and Prevention. Published May 26, 2017. Accessed July 10, 2020. https://www.cdc.gov/features/alzheimers-disease-deaths/index.html
115. Hui TS, Wong A, Wijesinghe R. A review on mortality risks associated with antipsychotic use in behavioral and psychologic symptoms of dementia (BPSD). *Ment Health Clin*. 2016;6(5):215-221. doi:10.9740/mhc.2016.09.215
116. Maust DT, Kim HM, Seyfried LS, et al. Antipsychotics, other psychotropics, and the risk of death in patients with dementia: number needed to harm. *JAMA Psychiatry*. 2015;72(5):438-445. doi:10.1001/jamapsychiatry.2014.3018
