# Supplementary material for: Electroconvulsive therapy for the acute management of severe agitation in dementia (ECT-AD): A modified study protocol
Source: PLoS One. 2024 Jun 28;19(6):e0303894. doi: 10.1371/journal.pone.0303894 (PMC11213353; doi:10.1371/journal.pone.0303894)
Supplement: S4 File — (PDF) [file pone.0303894.s004.pdf]

Mass General Brigham IRB
Mass General Brigham
399 Revolution Drive, Suite 710
Somerville, MA 02145
Tel: 857-282-1900
Fax: 857-282-5693

Notification of IRB Review

Protocol #: 2020P002276

Date:	August 11, 2021
To:	Forester, Brent, MD, MSC<br>McLean<br>Mass General Brigham > McLean > Division of Geriatric Psychiatry
From:	Mass General Brigham IRB<br>399 Revolution Drive, Suite 710<br>Somerville, MA 02145
Title of Protocol:	A Randomized Controlled Trial of Electroconvulsive Therapy Plus Usual Care versus Simulated-ECT Plus Usual Care for the Acute Management of Severe Agitation in Alzheimer's Dementia
Version/Number:	Version 3
Version Date:	04/13/2021
Sponsor/Funding/Support:	Proposal Title: A Randomized Controlled Trial of Electroconvulsive Therapy plus Usual Care versus Simulated-ECT plus Usual Care for the Acute Management of Severe Agitation in Alzheimer's Dementia (ECT-AD)<br>Principal Investigator: Forester, Brent<br>Immediate Sponsor: NIH<br>Award Number: 5R01AG061100-04<br>Fund #: 401474
IRB Continuing Review/Amendment #:	1/14
IRB Review Type:	Full
IRB Approval Date:	08/11/2021
Approval Effective Date:	08/11/2021
Approval/Activation Date:	08/11/2021
**Next Review:**	Continuing Review
**IRB Expiration Date:**	**08/04/2022**

Mass General Brigham IRB
Mass General Brigham
399 Revolution Drive, Suite 710
Somerville, MA 02145
Tel: 857-282-1900
Fax: 857-282-5693

This project has been reviewed and approved by the **Mass General Brigham IRB**. During the review of this project, the IRB specifically considered (i) the risks and anticipated benefits, if any, to subjects; (ii) the selection of subjects; (iii) the procedures for obtaining and documenting informed consent; (iv) the safety of subjects; and (v) the privacy of subjects and confidentiality of the data.

Please note that if an IRB member had a conflict of interest with regard to the review of this project, consistent with IRB policies and procedures, the member was required to recuse him/herself and, if applicable, leave the room during the discussion and vote on this project except to provide information requested by the IRB.

The following documents were reviewed and approved by the IRB. A PDF document listing all documents reviewed and approved by the IRB is available via the "Download" button in Insight.

**Protocol Summary**	**04/13/2021**
**Detailed Protocol**	**04/13/2021, Version 3**
**Device Brochure**	**07/13/2020**
**Device Brochure**	**07/13/2020**
**Consent Form**	**11/20/2020**
**Consent Form**	**12/07/2020**
**Consent Form**	**03/02/2021**
**Consent Form**	**04/13/2021**
**Consent Form**	**02/12/2021**
**Consent Form**	**02/04/2021**

As Principal Investigator, you are responsible for ensuring that this project is conducted in compliance with all applicable federal, state and local laws and regulations, institutional policies, and requirements of the IRB, which include, but are not limited to, the following:

1. Submission of any and all proposed changes to this project (e.g., protocol, recruitment materials, consent form, status of the study, etc.) to the IRB for review and approval prior to initiation of the change(s), except where necessary to eliminate apparent immediate hazards to the subject(s). Changes made to eliminate apparent immediate hazards to subjects must be reported to the IRB as an unanticipated problem.
2. Submission of a continuing review submission or institutional status report as required by the IRB and/or institution to continue the research, and submission of a final report when the project has been closed or completed.
3. Submission of any and all unanticipated problems, including adverse event(s) in accordance with the IRB's policy on reporting unanticipated problems including adverse events.
4. Obtaining informed consent from subjects or their legally authorized representative prior to initiation of research procedures when and as required by the IRB and, when applicable, documenting informed consent current IRB approved consent form(s) with the IRB-approval stamp in the document footer.
5. Informing all investigators and study staff listed on the project of changes and unanticipated problems, including adverse events, involving risks to subjects or others.

Mass General Brigham IRB
Mass General Brigham
399 Revolution Drive, Suite 710
Somerville, MA 02145
Tel: 857-282-1900
Fax: 857-282-5693

6. When investigator financial disclosure forms are required, submitting updated financial disclosure forms for yourself and for informing all site responsible investigators, co-investigators and any other members of the study staff identified by you as being responsible for the design, conduct, or reporting of this research study of their obligation to submit updated Investigator Financial Disclosure Forms for this protocol to the IRB if (a) they have acquired new financial interests related to the study and/or (b) any of their previously reported financial interests related to the study have changed.

IMPORTANT REMINDER: THE IRB HAS THE AUTHORITY TO TERMINATE PROJECTS THAT ARE NOT IN COMPLIANCE WITH THESE REQUIREMENTS.

Questions related to this project may be directed to IRB@partners.org

cc:

Brent Forester, MD, MSC, Principal Investigator, Division of Geriatric Psychiatry
