# Supplementary material for: Electroconvulsive therapy for the acute management of severe agitation in dementia (ECT-AD): A modified study protocol
Source: PLoS One. 2024 Jun 28;19(6):e0303894. doi: 10.1371/journal.pone.0303894 (PMC11213353; doi:10.1371/journal.pone.0303894)
Supplement: S7 File — (PDF) [file pone.0303894.s007.pdf]

The U.S. government does not review or approve the safety and science of all studies listed on this website.

Read our full disclaimer (https://clinicaltrials.gov/about-site/disclaimer) for details.

RECRUITING

Electroconvulsive Therapy (ECT) for Agitation in Dementia (AD) (ECT-AD)

ClinicalTrials.gov ID NCT03926520

Sponsor Brent Forester

Information provided by Brent Forester, Mclean Hospital (Responsible Party)

Last Update Posted 2023-10-23

Study Details Tab

Study Overview

Brief Summary

This study will explore the effect of ECT treatments plus usual care (ECT+UC) in reducing severe agitation in patients with moderate to severe dementia including Alzheimer's Disease, Vascular dementia, Frontotemporal dementia, and Dementia with Lewy Bodies. The study will also determine the tolerability/safety outcomes of ECT+UC.

Detailed Description

This study will be a single-arm, unblinded, non-randomized trial to determine the effect and safety of ECT for severe agitation in moderate to severe stage dementia, while also examining the durability of the acute treatment effect in an exploratory maintenance naturalistic design. We plan to enroll 50 participants with an estimated dropout rate of 20%. We expect 50 participants to complete at least 1 ECT treatment before moving into the 12-month naturalistic follow-up phase.

Feedback

Official Title

Effect and Safety of **Electroconvulsive Therapy** Plus Usual Care for the Acute Management of Severe Agitation in Dementia

Conditions

Alzheimer Dementia   Agitation,Psychomotor

Intervention / Treatment

- Device: Electroconvulsive Therapy (ECT)

Other Study ID Numbers

- 2020P002276

Study Start (Actual)

2021-01-28

Primary Completion (Estimated)

2024-05

Study Completion (Estimated)

2024-05

Enrollment (Estimated)

50

Study Type

Interventional

Phase

Not Applicable

Resource links provided by the National Library of Medicine

MedlinePlus Genetics (https://medlineplus.gov/genetics/) related topics: Alzheimer disease (https://medlineplus.gov/genetics/condition/alzheimer-disease)

MedlinePlus (https://medlineplus.gov/) related topics: Dementia (https://medlineplus.gov/dementia.html)

Genetic and Rare Diseases Information Center (https://rarediseases.info.nih.gov/gard) resources: Acute Graft Versus Host Disease (https://rarediseases.info.nih.gov/diseases/6544/acute-graft-versus-host-disease) Familial Alzheimer Disease (https://rarediseases.info.nih.gov/diseases/632/familial-alzheimer-disease)

FDA Drug and Device Resources (https://clinicaltrials.gov/fda-links)

Contacts and Locations

This section provides the contact details for those conducting the study, and information on where this study is being conducted.

Study Contact

Name: Jefferson Mattingly, BA
Phone Number:
617-855-3168
Email: jmattingly@mclean.harvard.edu

Study Contact Backup

Name: Maria DelPico, BS
Phone Number:
617-855-3168
Email: mdelpico@mclean.harvard.edu

United States

Georgia Locations

Atlanta, Georgia, United States, 30308
Recruiting
Emory Healthcare
Contact: Valeriya Tsygankova
valeriya.tsygankova@emory.edu
Principal Investigator: Adriana Hermida, MD

Massachusetts Locations

Click to view interactive map

Participation Criteria

Researchers look for people who fit a certain description, called eligibility criteria. Some examples of these criteria are a person's general health condition or prior treatments.

For general information about clinical research, read Learn About Studies (https://clinicaltrials.gov/study-basics/learn-about-studies).

Eligibility Criteria

Description

Inclusion Criteria

1. Diagnosis of Dementia, of the following subtypes,
    1. Alzheimer's dementia, according to NIA-AA Criteria for dementia
    2. Vascular dementia based on:

   i. History consistent with insidious onset of illness and gradual clinical decline ii. MRI evidence of microvascular ischemic disease (microinfarcts) iii. Physical and neurological examination do not indicate current or prior stroke c. Frontotemporal dementia d. Dementia with Lewy Bodies
2. Mini Mental Status Exam (MMSE) less than or equal to 15
3. Cohen-Mansfield Agitation Inventory Nursing Home Version (CMAI) score of 5 or more on at least one item or score of 4 on two items of aggression or physical nonaggression that holds potentially dangerous consequences including hitting (including self), kicking, grabbing onto people, pushing, throwing things, biting, scratching, spitting, hurting self or other, tearing things or destroying property, making physical sexual advances, trying to get to a different place, or intentional falling (items 1-11, 14, 15) OR one score of 5 or more in items of verbal aggression including screaming, making verbal sexual advances, and cursing or verbal aggression (items 22-24).
4. At least one failed pharmacological intervention to manage behavioral symptoms
5. Medically stable for safe administration of ECT verified by standard physical examination, urinalysis and serum chemistries and brain imaging when clinically indicated
6. Comprehension of English language
7. Authorized legal representative able and willing to give informed consent
8. Age 40 and above

Exclusion Criteria:

1. Current diagnosis of co-morbid delirium, measured by the Confusion Assessment Measure (CAM) and by clinical diagnosis
2. Diagnosis of vascular dementia due to stroke, based on:
    - History consistent with abrupt onset and step-wise progression of cognitive and functional decline
    - MRI scan within the past 12 months demonstrating evidence of hemorrhagic and embolic stroke
    - Physical and neurologic examination consistent with current or prior stroke
3. Lifetime or current diagnosis of Schizophrenia, Bipolar Disorder or Schizoaffective Disorder
4. Active substance use disorder within past 6 months
5. Treatment with ECT or other neurostimulation therapies (e.g., TMS or vagal nerve stimulation) within the past 3 months

Ages Eligible for Study

40 Years to 130 Years (Adult, Older Adult)

Sexes Eligible for Study

All

Accepts Healthy Volunteers

No

Study Plan

This section provides details of the study plan, including how the study is designed and what the study is measuring.

How is the study designed?

Design Details

Primary Purpose : Treatment
Allocation : N/A
Interventional Model : Single Group Assignment
Masking : None (Open Label)

Arms and Interventions

Participant Group/Arm	Intervention/Treatment
Experimental: ECT+UC group	Device: Electroconvulsive Therapy (ECT)<br>• Stimulus method of delivery will be RUL electrode placement, and ultra-brief (UB) pulse width (0.25-0.37ms). At the first ECT session, seizure threshold (ST) will be determined by titration with the empirical dose titration method and subsequent treatments will be approximately 6 times the ST. Following other NIMH sponsored multicenter ECT studies (PRIDE, U01 MH055495), stimulus settings will be adjusted as needed during the ECT course based on seizure quality and treatment efficacy. Participants will be administered anesthesia.

What is the study measuring?

Primary Outcome Measures

Outcome Measure	Measure Description	Time Frame
CMAI total score	The CMAI measures the efficacy of ECT+UC in reducing severe agitation in AD subjects. The CMAI is a 29-item scale with each item ranging from 1-7 in frequency with 7 being the highest and therefore worst outcome.	The CMAI will be collected through study completion,

		about 13 months

Secondary Outcome Measures

Outcome Measure	Measure Description	Time Frame
Alzheimer's Disease Cooperative Study-Clinical Global Impression of Change Scale (ADCS-CGIC)	The ADCS-CGIC gives a discrete score that ranges from 1-7 with 7 being the worst outcome.	The ADCS-CGIC will be collected for one month
Neuropsychiatric Inventory, Clinician Version (NPI-C)	The NPI-C is an improved version of the NPI composed of several domains of which we will use Agitation and Aggression, as well as their sum. The higher the frequency and/or severity within each domain, the worse the condition of the patient.	The NPI-C will be collected for one month
Pittsburgh Agitation Scale (PAS)	The PAS assesses four behavioral domains. Each domain has an intensity score ranging from 0-4 with 4 being the worst outcome.	The PAS will be collected for one month

Collaborators and Investigators

This is where you will find people and organizations involved with this study.

Sponsor

Brent Forester

Collaborators

- Mayo Clinic
- Pine Rest Christian Mental Health Services
- Emory University
- The Zucker Hillside Hospital
- Medical University of South Carolina

Investigators

- Principal Investigator: Brent P Forester, MD, MSc, Mclean Hospital
- Principal Investigator: George Petrides, MD, Northwell Health

Publications

The person responsible for entering information about the study voluntarily provides these publications. These may be about anything related to the study.

General Publications

No publications available

*** Find Publications about Study Results and related Pubmed Publications in the "Results" section of the study record.**

Study Record Dates

These dates track the progress of study record and summary results submissions to ClinicalTrials.gov. Study records and reported results are reviewed by the National Library of Medicine (NLM) to make sure they meet specific quality control standards before being posted on the public website.

Study Registration Dates

First Submitted

2019-04-19

First Submitted that Met QC Criteria

2019-04-23

First Posted

2019-04-24

Study Record Updates

Last Update Submitted that met QC Criteria

2023-10-19

Last Update Posted

2023-10-23

Last Verified

2023-10

More Information

Terms related to this study

Keywords Provided by Brent Forester, Mclean Hospital

ECT
Agitation
Alzheimer's
Dementia

Additional Relevant MeSH Terms

Brain Diseases
Central Nervous System Diseases
Nervous System Diseases
Neurocognitive Disorders
Mental Disorders
Tauopathies
Neurodegenerative Diseases
Dyskinesias
Neurologic Manifestations

Psychomotor Disorders
Neurobehavioral Manifestations
Behavioral Symptoms
Dementia
Alzheimer Disease
Psychomotor Agitation
Aberrant Motor Behavior in Dementia

Drug and device information, study documents, and helpful links

Studies a U.S. FDA-Regulated Drug Product

No

Studies a U.S. FDA-Regulated Device Product

Yes

Study Documents

No study documents available
