# Supplementary material for: Electroconvulsive therapy for the acute management of severe agitation in dementia (ECT-AD): A modified study protocol
Source: PLoS One. 2024 Jun 28;19(6):e0303894. doi: 10.1371/journal.pone.0303894 (PMC11213353; doi:10.1371/journal.pone.0303894)
Supplement: Attachment — Submitted filename: ECT AD Protocol_Response letter.docx [file pone.0303894.s009.docx]

**Maria I. Lapid, MD**

Department of Psychiatry and Psychology

April 9, 2024

Emily Chenette, PhD

Editor-in-Chief

PLOS ONE

Dear Dr. Chenette,

Thank you for reviewing our manuscript entitled “Electroconvulsive Therapy for the Acute Management of Severe Agitation in Dementia (ECT-AD): A Modified Study Protocol" (PONE-D-24-10130) for publication under Study Protocols in PLOS ONE journal.

In response to the comments sent to us, we have addressed the issues and have made appropriate revisions. Below is a point-by-point reply to comments followed by a brief description of the changes that have been made in the manuscript. We highlighted all the changes made to the manuscript by using the track changes mode, and also submit a clean version.

We we hope that you find it acceptable in its present form.

Respectfully yours,

Maria I. Lapid, M.D.

Professor of Psychiatry

Mayo Clinic Department of Psychiatry and Psychology

200 First St. SW

Rochester, MN 55905

<http://www.mayoclinic.org/biographies/lapid-maria-i-m-d/bio-20054523>

Phone: (507) 284-4159

Email: lapid.maria@mayo.edu

**Editor’s comments**:

Please send the manuscript back to the authors for:

**Comment**: CT registration

**Response**: We are registered on clinicaltrials.gov. Our ClinicalTrials.gov ID: NCT03926520. We attached a copy of our NCT registration as Supporting Information file S2.

**Comment**: SPIRIT checklist

**Response**: We uploaded the SPIRIT checklist as Supporting Information file S1.

**Comment**: SPIRIT schedule of enrolment, interventions, and assessments as the manuscript’s Figure 1

**Response**: We re-labeled the SPIRIT Figure as Figure 1 and re-numbered other figures accordingly.

**Comment**: protocol

**Response**: We uploaded the latest version of our protocol as Supporting Information file S3.

**Comment**: proof of peer review by external funder

**Response**: We uploaded the original National Institutes of Health Notice of Award dated 11/10/2018 as a proof of peer review by external funder as Supporting Information file S4.

**Comment**: “During our evaluation of the documents provided, we noted that your ethics approval letter did not cover the entire date range for participant recruitment. Before we can proceed further with the submission, please provide the ethics approval extension document(s)] for the study. If the document is in another language, please also provide an English translation. Please note that if this document is not included when your manuscript is resubmitted, it may be rejected."

**Response**: We relabeled the original IRB approval letter as Supporting Information file S5 and uploaded 3 more IRB approval letters that cover the entire date range for participant recruitment as Supporting Information files S6, S7, and S8.
